# Novel Potent and Selective DPP-4 Inhibitors: Design, Synthesis and Molecular Docking Study of Dihydropyrimidine Phthalimide Hybrids

**DOI:** 10.3390/ph14020144

**Published:** 2021-02-11

**Authors:** Ahmed A. E. Mourad, Ahmed E. Khodir, Sameh Saber, Mai A. E. Mourad

**Affiliations:** 1 Pharmacology and Toxicology Department, Faculty of Pharmacy, Port-Said University, Port-Said 42511, Egypt; ahmed.mourad@yahoo.com; 2 Pharmacology Department, Faculty of Pharmacy, Horus University, New Damietta 34518, Egypt; akhodir@horus.edu.eg; 3 Pharmacology Department, Faculty of Pharmacy, Delta University for Science and Technology, Gamasa City, Mansoura, Dakahlia 11152, Egypt; sameh.mohamed@deltauniv.edu.eg; 4 Medicinal Chemistry Department, Faculty of Pharmacy, Port-Said University, Port-Said 42511, Egypt

**Keywords:** DPP-4 inhibitors, T2DM, OGTT, DPPH, dihydropyrimidine, phthalimide

## Abstract

Background: Dipeptidyl peptidase-4 (DPP-4) inhibitors have emerged as anti-hyperglycemic agents that improve glycemic control in type 2 diabetic patients, either as monotherapy or in combination with other antidiabetic drugs. Methods: A novel series of dihydropyrimidine phthalimide hybrids was synthesized and evaluated for their in vitro and in vivo DPP-4 inhibition activity and selectivity using alogliptin as reference. Oral glucose tolerance test was assessed in type 2 diabetic rats after chronic treatment with the synthesized hybrids ± metformin. Cytotoxicity and antioxidant assays were performed. Additionally, molecular docking study with DPP-4 and structure activity relationship of the novel hybrids were also studied. Results: Among the synthesized hybrids, **10g**, **10i**, **10e**, **10d** and **10b** had stronger in vitro DPP-4 inhibitory activity than alogliptin. Moreover, an in vivo DPP-4 inhibition assay revealed that **10g** and **10i** have the strongest and the most extended blood DPP-4 inhibitory activity compared to alogliptin. In type 2 diabetic rats, hybrids **10g**, **10i** and **10e** exhibited better glycemic control than alogliptin, an effect that further supported by metformin combination. Finally, **10j**, **10e**, **10h** and **10d** had the highest radical scavenging activity in DPPH assay. Conclusions: Hybrids **10g**, **10i** and **10e** are potent DPP-4 inhibitors which may be beneficial for T2DM treatment.

## 1. Introduction

Diabetes mellitus is a rapidly growing chronic disease that threatens human health worldwide. T2DM is characterized by insulin resistance and impaired glucose homeostasis, resulting in hyperglycemia [1]. Currently, there are a number of antidiabetic drugs including sulfonylureas, meglitinides, thiazolidinediones, biguanides, α-glucosidase inhibitors aimed at suppressing hepatic glucose output, stimulating insulin release, mitigating glucose absorption, and increasing peripheral glucose utilization [2,3]. Unfortunately, the use of these drugs is associated with various side effects including weight gain and hypoglycaemia which deteriorate the optimum glycemic control over a longer time [4,5]. Therefore, many newer approaches with novel mechanism of action have been emerged for better T2DM management. Among these newer approaches, dipeptidyl peptidase-4 (DPP-4) inhibitors that gained extensive interest in T2DM treatment with proved long term efficacy and better glycemic control. Additionally, DPP-4 inhibitors play an important role in regeneration and differentiation of pancreatic β-cells and also proved to be well tolerated as well as decreasing the risk of hypoglycemia and cardiovascular side effects [6].

DPP-4 is widely expressed in most tissues and body fluids including the biliary tract, kidney, gastrointestinal tract, uterus and liver [7]. It is the key regulatory enzyme and a signaling factor of incretin hormones (insulin-stimulating hormones, glucagon-like peptide (GLP-1), and glucose-dependent insulinotropic polypeptide (GIP)) [8]. These hormones play a crucial role in promoting β-cell proliferation, increasing insulin biosynthesis, reducing β-cell apoptosis [9,10]. As a consequence of rapid degradation by DPP-4, both GLP-1 and GIP have very short half-life which is 1–2 minutes for the former and 7 minutes for the latter [11,12]. Currently, there are several structurally diverse DPP-4 inhibitors some of them have been approved on the market such as sitagliptin, alogliptin, linagliptin, gemigliptin, anagliptin and teneligliptin [13,14].

On the other hand, pyrimidines represented one of the most important versatile scaffolds in medicinal chemistry [15] that possess many activities including antidiabetic [16], anti-inflammatory [17], anticancer [18], antibacterial [19], antihypertensive [20], antifungal [21] and antitubercular [22]. Moreover, some of the synthesized N-methylated and N-benzylated pyrimidinediones have shown in vitro DPP-4 inhibitory activity in significant range [23]. In addition, excellent IC_50_ values were recorded for novel pyrimidinedione derivatives when evaluated for their DPP-4 inhibitory activity and in vivo anti-hyperglycemic efficacy [24].

On the other hand, phthalimide-containing compounds are precious synthetic scaffolds that showed different biological activities such as: antihyperglycemic [25], anti-inflammatory [26], antimicrobial [27], antihyperlipidimic [28], anticonvulsant [29], antiviral [30] and anticancer effects [31]. Additionally, a novel series of potent DPP-4 inhibitors bearing an N-phenylphthalimide skeleton has been synthesized and displayed higher potency compared to a well-known DPP-4 specific inhibitor [32,33]. Recently, some non-competitive DPP-4 inhibitors have been synthesized from a series of benzo[4,5]thieno[2,3-d]pyrimidine phthalimide derivatives and have shown better activity compared to DPP-4 inhibitors synthesized from amine precursors [34].

**Figure 1:**
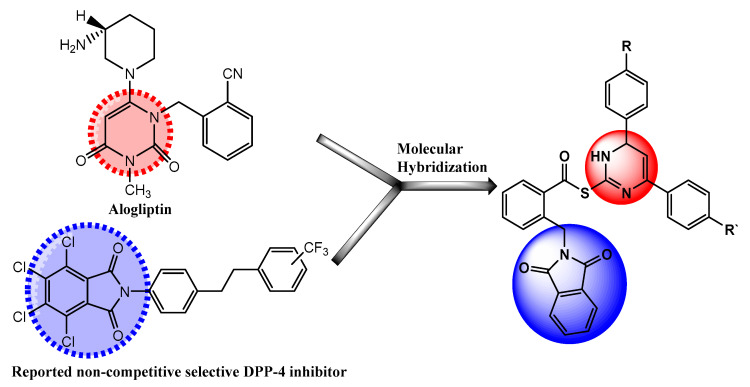
Diagram represents design of novel DPP-4 inhibitors.

Based on the aforementioned information, we aimed at combining pyrimidine and phthalimide moieties into a single candidate template for the purpose of synergistic activity (Figure 1). To better elucidate their DPP-4 inhibitory activity, our synthesized hybrids were subjected to in vitro DPP inhibition assay utilizing human recombinant DPP-4, DPP-8 and DPP-9; alogliptin used as a reference standard. Moreover, the effect of our compounds on in vivo DPP-4 activity was assessed in SD rats, where DPP-4 activity was evaluated in the blood over 2 days using alogliptin as a reference standard. Additionally, to gain better understanding of the role of our compounds on T2DM, we studied the effect of chronic treatment of the synthesized hybrids **10a–j** in presence and absence of metformin (MET) on HFD-induced T2DM in rats. Also, the target hybrids were tested for any possible antioxidant activity using DPPH method. Finally, the effect of synthesized hybrids **10a–j** on cell viability was examined on normal hepatic LO2 cells using MTT assay, to check for any cytotoxic effect. Furthermore, structure activity relationship and molecular docking analysis of the titled hybrids were performed to get a distinct insight about the binding mode and interactions of the compounds with DPP-4 active site.

## 2. Result and Discussion

### 2.1. Chemistry

**Scheme 1:**
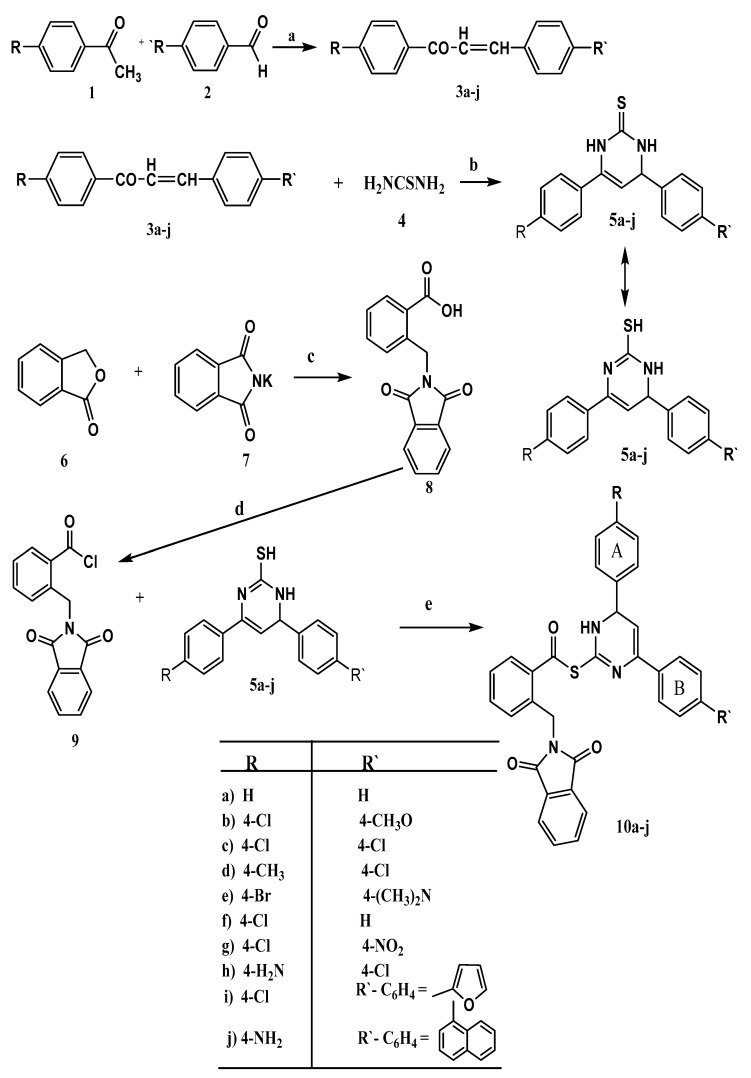
Synthesis of the target compounds **10a–j**. Reagents and conditions: (a) NaOH, dil. EtOH, rt; (b) NaOH, abs. EtOH; (c) DMF, reflux; (d) SOCl_2_, reflux, dry benzene, distillation; (e) abs. dichloromethane, TEA, rt.

The synthetic protocols applied to synthesize the target compounds **10a–j** are illustrated in Scheme 1. Substituted acetophenone **1** reacted with various aromatic aldehydes **2** in 10% sodium hydroxide solution following Claisen-Schmidt condensation conditions to afford exclusively the E-chalcone isomers **3a–j** in high yields [35]. These chalcones **3a–j** were further reacted with thiourea (**4**) in the presence of sodium hydroxide in abs. ethanol to give 4,6-diaryl-3,4-dihydropyrimidine-2(1H)-thiones **5a–j**. The structures of compounds **5e**, **g**, **i** and **j** were confirmed on the basis of their spectral data (FT-IR, ^1^H-NMR, ^13^C-NMR, MS) and elemental analyses (see Section 4).

Reduction of phthalimide gave phthalide (**6**) which on refluxing with potassium phthalimide in DMF afforded α-phthalimido-*o*-toluic acid (**8**). Reaction of **8** with thionyl chloride brought about α-phthalimido-*o*-toluyl chloride (**9**) [36]. Treatment of 4,6-diaryl-3,4-dihydropyrimidine-2(1H)-thiones **5a–j** with **9** in dichloromethane, and in the presence of triethyl amine (TEA) as a base brought about the target compounds (4,6-diaryl-1,6-dihydropyrimidin-2-yl)-2-((1,3-dioxoisoindolin-2-yl)methyl) benzothioates **10a–j**. Notably, the S-substituted product was isolated as nucleophilicity of sulphur is greater than nitrogen and no N-substituted product was isolated. The structure of **10a–j** hybrids were elucidated on the bases of both spectral data (IR, ^1^H-NMR, ^13^C-NMR, MS) and elemental analyses.

In the IR spectra the following peaks were observed: 3476–3384 cm^−1^ (NH), 3071–2918 cm^−1^ (Ar-CH), 1774–1770 cm^−1^, 1725–1714 cm^−1^ (CO), 1677–1603 cm^−1^ (C=N), 1595–1502 cm^−1^, 1491–1467 cm^−1^ (C=C). Beside the δ values of OCH_3_, CH_3_, Nme_2_, NH_2_ side chains in the ^1^H-NMR spectra of the hybrids **10b**, **10d**, **10e**, **10h** and **10j** which appear at 3.76, 2.34, 2.52, 6.19 and 6.17, respectively, the δ values of the other functional groups fall in the following ranges: NH:11.52–11.19 ppm; Ar-H: 8.51–6.75 ppm; HC=CH-Ar: 6.68–6.35 ppm; CH-Ar: 5.29–5.27 ppm; CH_2_: 5.08–5.01 ppm. Also, the ^13^C-NMR spectra of some selected compounds were resolved in appropriate regions confirming the assigned structures. Both mass spectra and elemental analyses of **10a–j** hybrids are a further support of their structures by giving correct molecular formula and molecular ion peaks.

### 2.2. Biological Evaluation

#### 2.2.1. In Vitro DPP Inhibition Assay

All the target hybrids listed in Table 1 were evaluated for the inhibition of human recombinant DPP-4 and alogliptin was chosen as a reference compound. Additionally, all the synthesized hybrids were tested for the inhibition of DPP-8 and DPP-9 which is closely related to DPP-4 and whose inhibition is associated with great toxicity.

Interstingly, hybrids **10g** and **10i** identified as the most potent DPP-4 inhibitors with picomolar potency, exhibiting IC_50_ values of 0.51 and 0.66 nM, respectively; which represented 11.2, 8.6 fold superior activity than alogliptin. The potent DPP-4 inhibitory activity of these compounds may be attributed to electronic behavior of the nitro group in ring B of hybrid **10g** and the small size of the furanyl group in ring B of hybrid **10i**, which facilitated the formation of hydrogen bond with DPP-4 active site.

Of considerable interest, the mesomeric effect raised from 4-Me_2_N group in ring B as well as 4-bromo substitutions in ring A of hybrid **10e** markedly boosted DPP-4 inhibition resulted in low nanomolar potency activity (1.42 nM, 4 fold increased activity than that of alogliptin). Moreover, introducing an electron donating groups (methyl group in ring A and methoxy group in ring B) retained the potent DPP-4 inhibitory activity of hybrids **10b** and **10d**, whose activities were 1.5 fold and 2.6 fold the activity of alogliptin with IC_50_ values of 3.78, 2.19 nM, respectively. According to the basis of electron donating properties, hybrid **10j** expected to be the most potent DPP-4 inhibitor but on the contrary, the DPP-4 inhibitory potency is eroded (IC_50_ = 7.35 nM). The probable reason could be the worse fit of the naphthyl group in ring B with more steric inhibition into the receptor pocket compared to the phenyl ring in the other synthesized compounds. On the other hand, the enzyme inhibition activity was dramatically decreased in the unsubstituted phenyl group and upon introducing the electron withdrawing chlorine atom in **10h**, **10c** and hybrids **10f** which was confirmed by their high IC_50_ values compared to alogliptin. Out of these, it might be concluded that the substituent electronic character and the steric volume of the phenyl ring considerably influenced the DPP-4 inhibition activity.

On the other hand, all the synthesized hybrids targeted DPP-8 and DPP-9 at IC_50_ exceeding 100 µM which indicates that our compounds have superior selectivity for DPP-4 and neglected affinity toward DPP-8 and DPP-9.

#### 2.2.2. Effects of Synthesized Hybrids (**10a–j**) on Cell Viability

**Figure 2:**
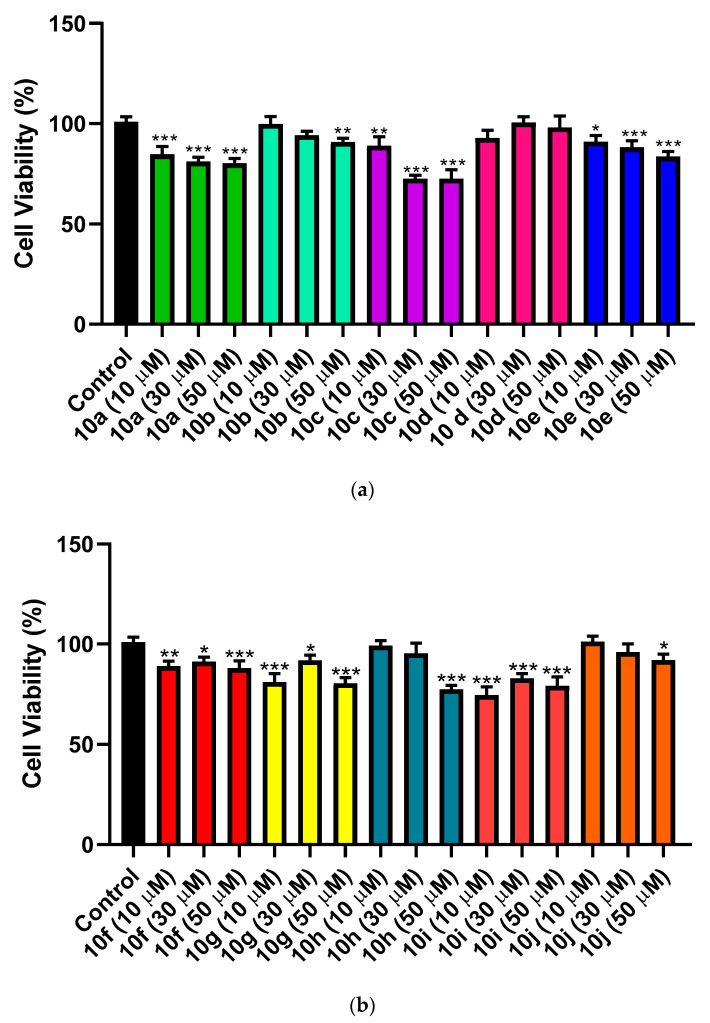
(**a**) Effect of hybrids **10a–e** on viability of normal hepatic LO2 cells. (*n* = 5). (**b**) Effect of hybrids **10f–j** on viability of normal hepatic LO2 cells (*n* = 5). *** Significant from control group at *p* < 0.001, ** Significant from control group at *p* < 0.01, * Significant from control group at *p* < 0.05.

The cytotoxic effect of the synthesized hybrids **10a–j** was examined on normal hepatic LO2 cells using MTT assay. The test was done using 3 different concentrations (10 µM, 30 µM and 50 µM) for each compound. As shown in (Figure 2a), hybrid **10c** had the most cytotoxic effect with 72.57% and 72.50% cell viability at 30 µM and 50 µM concentration, respectively. However, hybrid **10i** came in the second place with cell viability percent of 74.60 at 10 µM concentration, followed by hybrid **10h** with cell viability percent of 77.47 at 50 µM concentration, as presented in (Figure 2b). While, the cell viability percent for other synthesized hybrids ranged from 80.13 to 101.2. In conclusion, our compounds showed weak cytotoxic effect against hepatic LO2 cells.

#### 2.2.3. Effect of Synthesized Hybrids (**10a–j**) on In Vivo DPP-4 Activity

**Figure 3:**
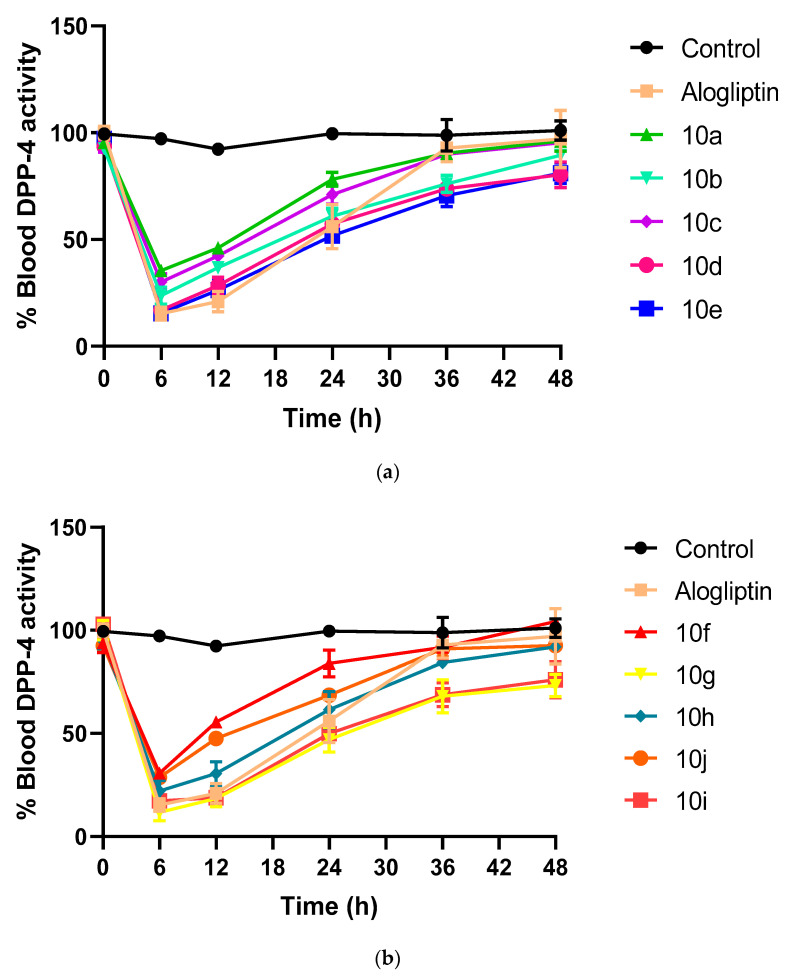
(**a**). The in vivo DPP-4 activity of **10a–e** hybrids and alogliptin within 48 h. (*n* = 3). (**b**) The in vivo DPP-4 activity of **10f–j** hybrids and alogliptin within 48 h. (*n* = 3).

The effect of the synthesized hybrids **10a–j** on blood DPP-4 activity was investigated in SD rats, as shown in (Figure 3a,b). The hybrids were administrated in a single oral dose of 10 mg/kg and in vivo DPP-4 activity was evaluated over 2 days, using alogliptin as reference compound. Notably, among all tested hybrids and aloglipin, hybrid **10g** has both the strongest and longest DPP-4 inhibitory action, with 18.45% and 47% DPP-4 blood activity at 12 h and 24 h, respectively, followed by hybrid **10i** with DPP-4 blood activity of 18.8% and 49.9% at 12 h and 24 h, respectively. While, alogliptin achieved DPP-4 blood activity of 20.95% and 56.1% at 12 h and 24 h, respectively. Importantly, hybrids **10g** and **10i** also showed extended DPP-4 inhibitory activity at 48 h with blood DPP-4 activity of 73.3% and 76%, respectively, while, alogliptin DPP-4 blood activity was 97.05%. Worthily, hybrids **10g**, **10i** and **10e** had the strongest in vitro and in vivo DPP-4 inhibiting activity.

#### 2.2.4. Effect of Chronic Treatment of Compounds **10a–j** with or without MET on HFD-Induced Type 2 Diabetic rats

**Figure 4:**
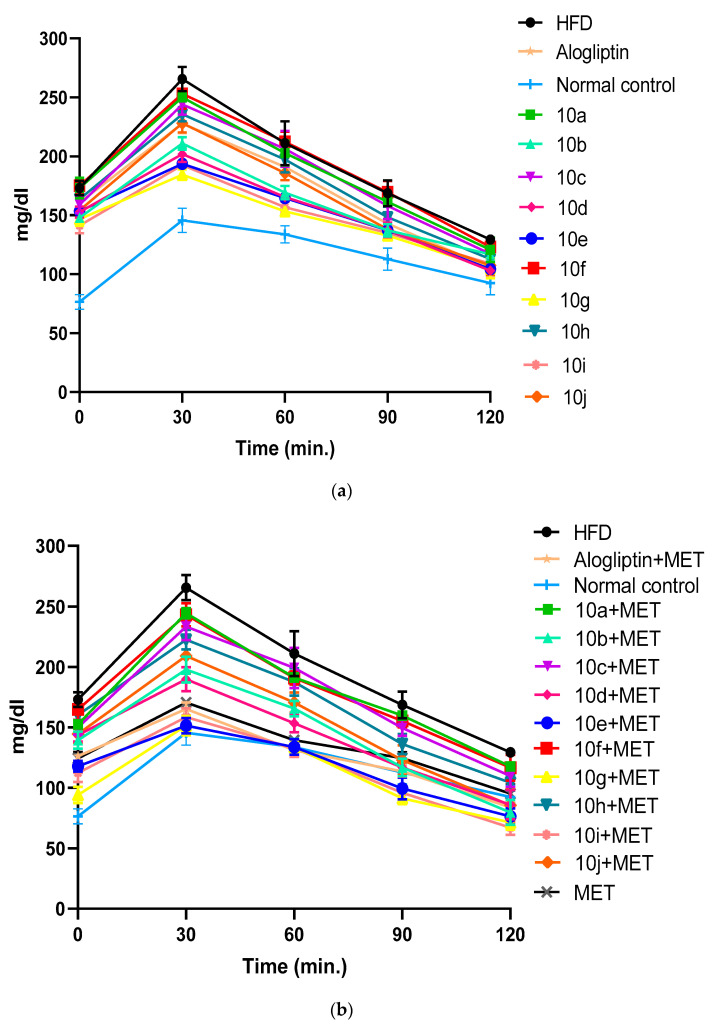
(**a**) Chronic effect of hybrids **10a–j** and alogliptin administration on blood glucose levels during an OGTT in type 2 diabetic rats. Data are presented as mean ± SEM (*n* = 7). (**b**) Chronic effect of combined administration of **10a–j**/MET, alogliptin/MET on blood glucose levels during an OGTT in type 2 diabetic rats. Data are presented as mean ± SEM (*n* = 7).

**Figure 5:**
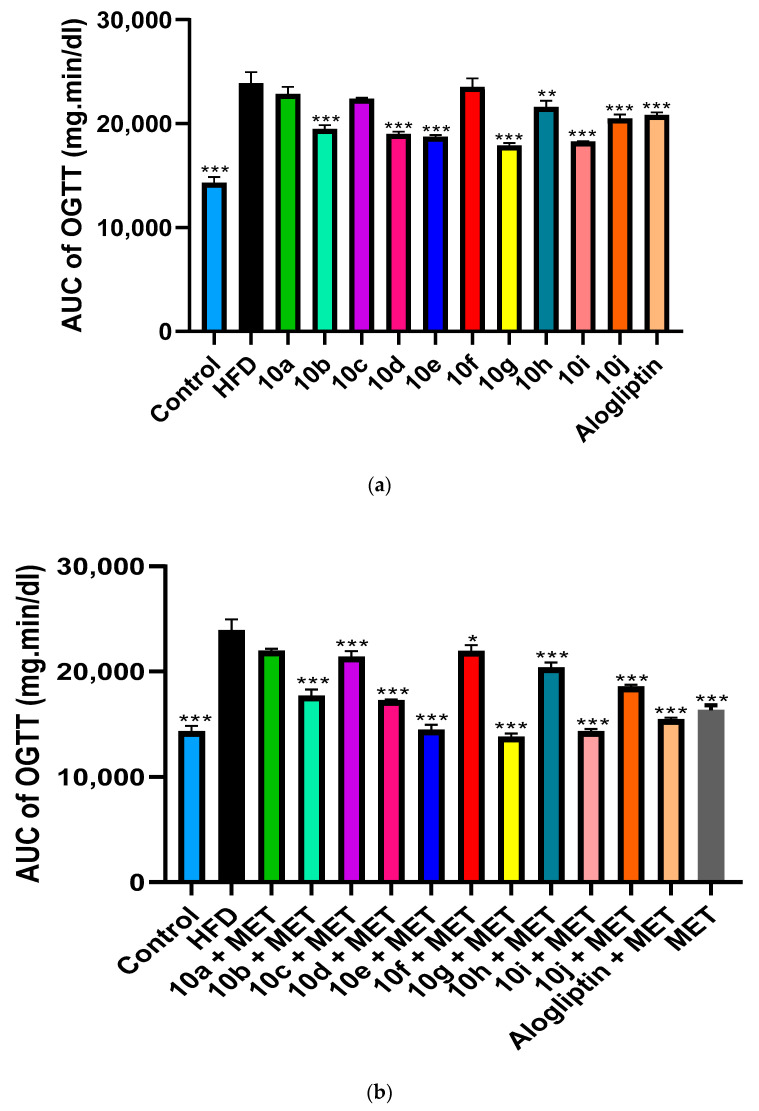
(**a**) Chronic effect of hybrids **10a–j** and alogliptin administration on area under the curve (AUC) of OGTT in type 2 diabetic rats. Data are presented as mean ± SEM (*n* = 7). *** Significant from diabetic control group at *p* < 0.001, ** Significant from diabetic control group at *p* < 0.01. (**b**) Chronic effect of combined administration of **10a–j**/MET, alogliptin/MET on area under the curve (AUC) of OGTT in type 2 diabetic rats. Data are presented as mean ± SEM (*n* = 7). *** Significant from diabetic control group at *p* < 0.001, * Significant from diabetic control group at *p* < 0.05.

HFD significantly induced insulin resistance in SD rats as evident by extremely significant increase in the AUC of OGTT in non-treated diabetic group compared to control group, as shown in (Figure 4a,b). We studied the chronic effect of oral administration of hybrids **10a–j** at a dose of 10 mg/kg/day in absence and presence of MET, on insulin resistance in type 2 diabetic rats. In absence of MET, hybrids **10g, 10i** and **10e** significantly improved glucose tolerance above alogliptin, as evident by the reduction in the AUC of OGTT when compared to non-treated diabetic group, (Figure 5a,b). Moreover, oral administration of MET (150 mg/kg/day) together with hybrids **10a–j**, further enhanced insulin sensitivity with a profound reductions in AUC of OGTT almost in all treated groups. Interestingly, MET addition to **10g**, **10i** and **10e**-treated groups reduced AUC of OGTT by 22.93%, 21.7% and 22.82%, respectively. Accordingly, glucose tolerance in **10g**/MET treated group reached a normal level with AUC equals 13787 ± 201 mg.min/dL compared to 14305 ± 318 mg.min/dL for normal control group. Similarly, addition of MET to alogliptin treated group reduced AUC of OGTT from 20835 ± 146 mg.min/dL in alogliptin treated group to 15451 ± 110 mg.min/dL in MET/alogliptin treated group.

#### 2.2.5. Antioxidant Activity

The in vitro antioxidant activity of the synthesized hybrids **10a–j** was tested depending on DPPH method using ascorbic acid as standard. The results in Table 2 revealed that all the synthesized compounds showed good antioxidant activity, wherein hybrids **10j**, **10e**, **10h** and **10d** have the highest radical scavenging activity in DPPH assay at 100 µM concentration.

#### 2.2.6. Molecular Docking Study: Docking at the DPP-4 active site

**Figure 6:**
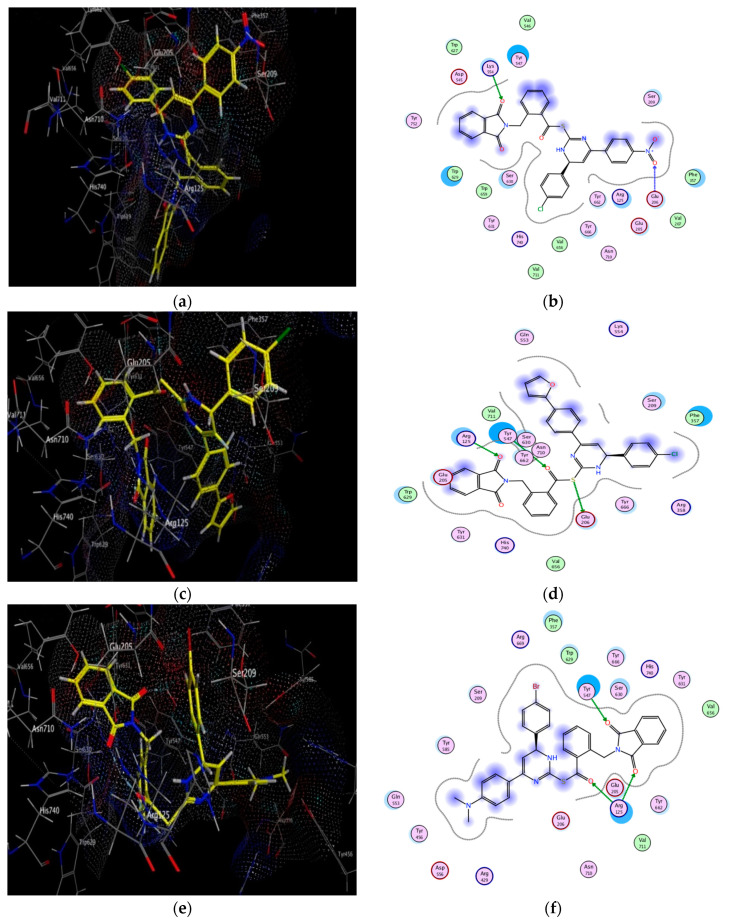

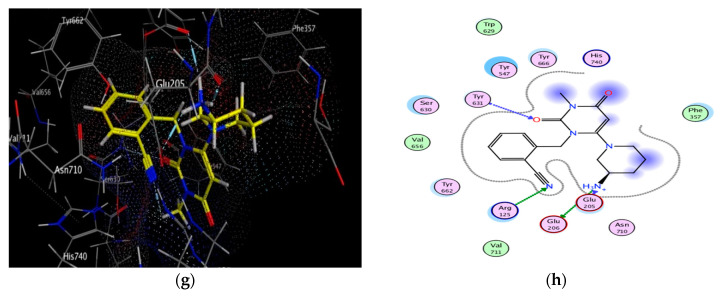
Docking and binding pattern of compounds **10g** (**a**,**b**), **10i** (**c**,**d**), **10e** (**e**,**f**) and alogliptin (**g**,**h**) showing interactions with different amino acid residues found in the active site of DPP-4 (PDB code: 3G0B).

To verify the protein-ligand binding properties and the key structural features required for the inhibitory activity of the novel synthesized hybrids **10a–j** with the protein DPP-4, a molecular docking study was performed using the molecular docking program MOE. The results of energy binding scores and binding interactions were depicted in Table 3 and Figure 6. The 3D crystal structure of DPP-4 was obtained from the Protein Data Bank [PDB code 3G0B, resolution 2.25 Å (www.rcsb.org, accessed on 10 February 2021)] and alogliptin was used as a reference DPP-4 inhibitor. DPP-4 active site is formed of two main binding pockets Figures S1 and S2 in addition to Figure S2 extensive subsite pocket. Figure S1 pocket is formed of highly hydrophobic residues Ser 630, Tyr 631, Tyr 547, Val 656, Trp 659, Tyr 662, Tyr 666, Asn 710, Val 711 and His 740), Figure S2 pocket (formed by Glu 205 and Glu 206 dyad and Arg 125) [37]. The Figure S2 pocket is surrounded by residues of Val 207, Ser 209, Arg 358, Phe 357, Lys 544, Trp 627, Trp 629 and Asp 708 which constitute the Figure S2 extensive subsite [38].

The SiteMap analysis highlighted the importance of the Tyr 547, Lys 554, and Trp 629 residues as a top-ranked binding pocket of DPP-4 in the formation of the enzyme–inhibitor complex [39]. Moreover, the hydroxyl group of Tyr 547 plays an oxyanion-stabilizing role in the DPP-4 catalytic mechanism as well as Trp 629 residue forms catalytic Figure S2 pocket in this protease [40]. The DPP-4 inhibitory activity is markedly increased when the inhibitor can interact to Figures S1 and S2 pockets. Interestingly, studies suggested that only few inhibitors can bind into Figure S2 extensive subsite and enhancing the bioactivity of DPP-4 inhibitors through the interactions with Lys 554 so it is the target of DPP-4 inhibitor selectivity [41].

It was obvious from the results depicted in Table 3 that nearly all of the novel hybrids achieved energy binding scores higher than that of alogliptin. Additionally, hybrids **10b**, **10d**, **10e**, **10g**, **10i** bind efficiently with the key amino acids located in the receptor pockets which explained the potent DPP-4 inhibitory activity of these hybrids. Of considerable interest, hybrid **10g** recorded the highest energy binding score and the lowest in vitro DPP-4 IC_50_ inhibitory activity among the other synthesized hybrids *via* binding mainly to the Figure S2 pocket and Figure S2 extensive subsite through formation of two strong hydrogen bonds between both oxygen of the free nitro group and phthalimido carbonyl group with Glu 206 and Lys 554 which have a vital role in the enhancement of DPP-4 affinity. However, the binding mode showed that the phthalimido carbonyl oxygen and S atom of hybrid **10i** participated in a hydrogen-bonding network with Arg 125 and Glu 206 residues in Figure S2 pocket together with another strong hydrogen bond formed with Tyr 547 in Figure S1 pocket. Similarly, compound **10d** was buried in Figures S1 and S2 pockets *via* formation of four hydrogen bonds as well as π-H bond with Arg 125 and Tyr 547.

Notably, one hydrogen bond is formed between one of the phthalimido carbonyl oxygen of compound **10e** and free OH group of Tyr 547 residue in Figure S1 pocket together with another three hydrogen bonds between the another carbonyl oxygen and the free NH_2_ of Arg 125 in Figure S2 pocket. However, hybrid **10b** stacked with the side chain of Lys 554 and Trp 629 in Figure S2 extensive subsite *via* weak π-cation and π-hydrogen interactions which explained its higher IC_50_ value comparable to the previously mentioned hybrids but its DPP-4 inhibitory activity still higher than that of alogliptin. On the other hand, in Figure S2 pocket, the nitrogen of the free amino group of hybrid **10j** is responsible for binding with Glu 205 and Glu 206 residues through two hydrogen bonds together with another hydrogen bond formed with Tyr 631 in Figure S1 pocket. Moreover, the phenyl ring of that compound provided additional binding affinity with Figure S2 extensive subsite *via* H-π interaction with Trp 629 residue while the naphthyl ring forms another π-H stacking with Tyr 456 side chain.

However, the electron withdrawing effect of chlorine atom in hybrid **10c** negatively affects its binding with the receptor site and hence its DPP-4 inhibitory activity. In addition hybrid **10a** binds weakly with the receptor site *via* only one hydrogen bonding with Arg 125. Meanwhile, **10f** and **10h** exhibited weak DPP-4 inhibitory activity as they could not be able to bind to the crucial amino acids responsible for the activity. Finally, it can be concluded that most of the titled synthesized hybrids interact with DPP-4 protein in distinct manner and thus are able to inhibit its activity efficiently.

## 3. Structure Activity Relationship

**Figure 7:**
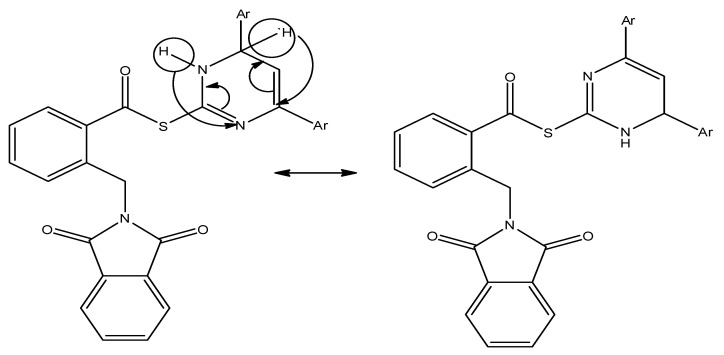
Equivalence of positions 2 and 4 in dihydropyrimidine.

Based on the aforementioned results, it becomes clear that the electronic nature of the substituents and the steric factor greatly affect the biological activity of the synthesized hybrids, so the present SAR study focused on the effect of substituents on position 4 in the aryl groups that located in positions 2 and 4 of dihydropyrimidine as in hybrids **10a–h**, as well as the furanyl and 1-naphthyl groups in hybrids **10i** and **10j**. It is worth noting that positions 2 and 4 in dihydropyrimidine are equivalent due to the migration of hydrogens and electron transition which is cleared in (Figure 7).

Moreover, the structures of most of the title compounds **10a–j** contain a 4-chlorophenyl group. Consequently, the compounds under investigation are related structurally to the substituents attached to the other phenyl group. On the basis of the electron donating effect of both the 1-naphthyl and 4-aminophenyl groups and the lipophilic character of the latter, hybrid **10j** should be the top ranked electron donor and consequently expected to be the most potent DPP-4 inhibitor *via* formation of more strong H-bonds with the receptor binding sites but on the contrary, the steric factor of the 1-naphthyl and 4-aminophenyl groups hinder the efficient binding with the receptor binding site, indicating that the steric factor is more effective, but it is still potent enough for inhibiting the enzyme activity as its IC_50_ was slightly less than that of alogliptin. Interestingly, hybrid **10g** achieved the most potent DPP-4 inhibitory activity owing to the electron donating properties and the unique electronic ionic behavior of the nitro group leading to higher ability to form strong H-bond with Figure S2 extensive subsite as evidenced by its higher energy binding score. 

Additionally, the DPP-4 inhibitory activity is markedly increased thanks to the lipophilic character and the small size of the furanyl group in hybrid **10i** which enhance its fit to the receptor site, and accordingly its ability to form strong H- bond with the receptor site as evidenced by its molecular docking results. On the other hand, the lipophilic electron donating character of the methyl group, *via* hyperconjugative effect, in hybrid **10d** resulted in an increase of its ability to form more H-bonds which, in turn, reflected on the marked difference in DPP-4 inhibitory IC_50_ values compared to that of the corresponding electron-withdrawing chlorine-atom in hybrid **10c**. Moreover, the mesomeric effect raised from 4-Me_2_N group as well as 4-bromo atom in **10e** hybrid seemed to be the most effective factor than hyperconjugative effect of 4-methyl and mesomeric effect of 4-chloro in hybrid **10d**, that led to stabilization of H-bonding and consequently better DPP-4 inhibiting activity. Meanwhile, the lipophilic electron donating methoxy group of hybrid **10b** experienced much higher DPP-4 inhibiting activity than the hydrophilic electron-withdrawing chlorine-atom hybrid **10f** which exhibited much weaker binding interaction with the key amino acid residue in the receptor site as evidenced by its high IC_50_ value. Finally, it is concluded from the aforementioned SAR results that the electronic nature, the steric factor and lipophilicity of the substituents greatly affected the biological activity of the system incorporated.

## 4. Materials and Methods

### 4.1. Animals

Male Sprague Dawley (SD) rats (6–7 weeks old and 100–120g body weight) were purchased from Abu-rawash Animal House (Giza, Egypt). Rats were allowed to acclimatize for one week with free access to food and water and kept under controlled laboratory conditions of humidity and 12 h light-dark cycle. All experimental protocols were approved by the Research Ethics Committee of the Faculty of Pharmacy, Port-said University (R 20.5.1527).

### 4.2. Experimental Design

After acclimatization period, rats were randomly divided into 25 groups, each of seven rats. The first group was normal control group which maintained on normal rat chow for the entire experiment period (16 weeks). The second group served as diabetic control group in which T2DM was induced by HFD that consisted of casein (30%), raw beef fat (Suet, 40%), wheat flour (7%), glucose (10%), salt mixture (6%), vitamin mixture (3%), and bran (4%), for 16 weeks. The remaining groups consumed HFD for 16 weeks, however, in the last four weeks, HFD was administrated together with each compound from our synthesized hybrids **10a–j** at a dose of 10 mg/kg/day, orally, or with alogliptin (Takeda Pharmaceuticals, Deerfield, IL, USA) at a dose of 20 mg/kg/day, orally, with and without MET (Alfa Aesar, ThermoFisher Inc., Haverhill, MA, USA) at a dose of 150 mg/kg/day, orally.

### 4.3. In Vitro DPP-4, DPP-8 and DPP-9 Inhibition Assay

DPP-4 inhibition was assessed, as previously described [42,43], based on a continuous fluorometric assay method in which the substrate Gly-Pro-AMC (Gly-Pro-7-amido-4-methylcoumarin hydrobromide) was split by DPP-4 to release fluorescent aminomethyl coumarin (AMC). The liberated AMC was continuously monitored using an excitation wavelength of 360 nm and an emission wavelength of 460 nm using an EnVision microplate reader (PerkinElmer, Waltham, MA, USA). The reaction mixture consisted of different concentrations of the synthesized hybrids **10a–j**, 20 µU/µL of recombinant human DPP-4 (Sigma Aldrich, St. Louis, MO, USA), 10 µM Gly-Pro-AMC (Sigma Aldrich, St. Louis, MO, USA) and assay buffer (150 mM NaCl, 25 mM 4-(2-hydroxyethyl)-1-piperazineethanesulfonic acid (HEPES), 0.12 mg/ml bovine serum albumin (BSA)) and pH of 7.5. The assay was carried out in quadruplicate. IC_50_ was calculated using GraphPad Prism 8 software (San Diego, CA, USA). Similar to the DPP-4 assay, DPP-8 and DPP-9 inhibition assay was performed and the pH of the assay buffer was 8.

### 4.4. Cytotoxicity Test

We assessed any possible cytotoxic effect of our synthesized hybrids **10a–j** on hepatic LO2 cells using MTT assay. The cells, in DMEM (Gibco, Invitrogen), were seeded in 96 well plates at density of 1 × 104/well. The synthesized compounds were added to the cells and incubated for 48 h at 37 °C and 5% CO_2_. Then, MTT reagent was added and the cells were re-incubated for further 4 h. After discarding the supernatants, the formed formazan crystals were dissolved in DMSO. The intensity of color was assessed using microplate reader (Bio-Rad Laboratories, Hercules, CA, USA) at 490 nm wavelength. 

### 4.5. In Vivo DPP-4 Inhibition Assay

The assay was performed using male SD rats (*n* = 3 per group), as previously described [44]. The synthesized hybrids **10a–j** were suspended in 0.5 carboxymethyl cellulose (CMC) and administrated orally at a single dose of 10 mg/kg.

Blood samples were collected at time points 0, 6, 12, 24, 36 and 48 h. Plasma samples were extracted and kept at −20 °C until analysis time. Similar to the in vitro DPP-4 assay principle, plasma samples were mixed with equal volumes of Gly-Pro-AMC in assay buffer (100 mM HEPES, 0.1 mg/ml BSA) and pH of 7.5. The liberated AMC was continuously monitored using an excitation wavelength of 360 nm and an emission wavelength of 460 nm using EnVision microplate reader (PerkinElmer, Waltham, MA, USA). The data were expressed as% blood DPP-4 activity.

### 4.6. In Vivo Oral Glucose Tolerance Test (OGTT) in SD Rats

At the end of treatment regimen that lasted for four weeks; using our synthesized hybrids **10a–j** with and without MET, rats were fasted overnight (12 h) with free access to water. Oral glucose tolerance test (OGTT) was performed as previously described [45]. Briefly, blood samples were collected from retro-orbital sinuses veins using glass capillaries at 0 time, 30, 60, 90 and 120 min after oral glucose loading (2 g/kg) and glucose concentration was determined with an automatic blood glucose meter (Accu-Chek; Roche Diagnostics, Rotkreuz, Switzerland).

### 4.7. Antioxidant Activity (DPPH Method)

DPPH (1,1-diphenyl-2-picrylhydrazyl) free radical is stable at rt and has a purple colour with a maximum absorption at 517 nm. This free radical is reduced by antioxidant molecules into 1,1-diphenyl-2-picrylhydrazine with resultant discolouration of free radical; this discolouration is stoichiometric with regard to the captured electrons. Accordingly, the fluctuation in the absorbance is an indicator for the antioxidant activity. Our synthesized hybrids **10a–j** (100 µM) were mixed with DPPH (100 µM) and left for 20 min at rt, the absorbance was measured at 517 nm.

### 4.8. Statistical Analysis

Results were expressed as means ± standard error of the mean (SEM) and were analyzed for statistically significant differences using one-way analysis of variance (ANOVA). P values less than 0.05, 0.01 and 0.001 were considered significant. GraphPad Prism^®^ 8 was used for statistical calculations (GraphPad Software, St. Louis, MO, USA).

### 4.9. Molecular Docking

Molecular docking studies of the synthesized hybrids **10a–j** and alogliptin were performed using MOE software. Ligands were built into the builder interface of the MOE program and their energies were minimized until a root mean square deviation (RMSD) gradient of 0.01 kcal/mol and root mean square (RMS) distance of 0.1 Å with MMFF94× (Merck molecular force field 94×) force-field and the partial charges were automatically calculated. The X-ray crystallographic structure of DPP-4 (PDB code: 3G0B) was downloaded from protein data bank (www.rcsb.org, accessed on 10 February 2021). The enzymes were prepared, the hydrogens were added then the atoms connection and type were checked with automatic correction. The obtained poses were studied and the poses showed best ligand-enzyme interactions were selected and stored for energy calculations.

### 4.10. Chemistry

Melting points were determined by open tube capillary on a Mel-Temp hot stage apparatus (Stuart, Staffordshire, UK) on the Celsius scale and are uncorrected. Reactions were monitored by thin-layer chromatography on silica gel PF254 plates (Merck, Darmstadt, Germany) using dichloromethane/ethyl acetate (9:1) as mobile phase, and spots were detected with UV analyses lamp at 254 and 366 nm. IR spectra were recorded as KBr disks on a Fourier Transform (FT-IR) spectrophotometer (Bruker, Billerica, MA, USA). ^1^H-NMR and ^13^C-NMR spectra were recordedout on a Bruker NMR spectrometer operating at 400 MHz and 100 MHz, respectively, using TMS as internal reference. Chemical shifts (δ values) are given in parts per million (ppm) using CDCl_3_ (7.26) or DMSO-_d6_ (2.49) as solvents and coupling constants (J) in Hz. Splitting patterns are designated as follows: s, singlet; bs, broad singlet; d, doublet; t, triplet; m, multiplet. GCMS: Mass spectra was recorded on a LCMS-QQQ instrument (Agilent, Santa Clara, CA, USA). Elemental analyses were performed on a 2400 CHN elemental analyzer (Perkin–Elmer, Waltham, MA, USA) and were within ± 0.4% of theoretical values unless otherwise specified. 

### 4.11. Materials

Chemicals and solvents are of commercial grade, and purchased from Alfa Aesar (Haverhill, MA, USA), Cambrian Chemicals (Ontario, CA), Sigma-Aldrich (St. Louis, MO, USA), Acros Organics (Haverhill, MA, USA), Fluka (Gillingham, Dorset, UK), Merck (Ontario, CA), and El-Nasr Pharmaceutical Chemical Company (Cairo, Egypt). Chemicals were used without purification. Solvents were purified following the reported methods.

Substituted 1,3-diaryl-2-propene-1-ones **3** were prepared according to a procedure described elsewhere in the literature [46,47,48,49,50,51]. α-Phthalimido-*o*-toluic acid (**8**) and α-phthalimido-*o*-toluyl chloride (**9**) were prepared according to the literature [36]. 4,6-Diaryl-3,4-dihydropyrimidine-2(*1H*)-thiones: **5a**, **5b**, **5c**, **5f** and **5h** were synthesized following the reported procedure in the literatures [52,53,54,55].

### 4.12. Synthesis 

#### 4.12.1. General Procedure for Synthesis of 4,6-diaryl-3,4-dihydropyrimidine-2(1H)-thiones **5d–g**, **i**, **j** [53]

A mixture of 1,3-diaryl-2-propen-1-one derivatives **3** (2.404 mmol), prepared by Claisen-Schmidt condensation between substituted acetophenones **1**, benzaldehydes **2**, and thiourea **4** (2.404 mmol) and NaOH (2.404 mmol) in absolute ethanol (25 mL) was heated under reflux for 4–6 h. The reaction progress was monitored by TLC using dichloromethane/ethyl acetate (9:1). The reaction mixture was cooled to rt and poured onto crushed ice. The solid product was filtered off, washed with water and crystallized from ethanol to yield the following pure titled compounds.

##### 4-(4-Chlorophenyl)-6-(p-tolyl)-3,4-dihydropyrimidine-2(1H)-thione (**5d**)

Yield: 0.574 g, 76% (pale yellow solid); m.p. 138–9 °C; IR (KBr, cm^−1^) υ: 3183, 2967 (Ar-CH), 1672 (C=N), 1551, 1462 (C=C), 1178 (C=S). ^1^H-NMR (CDCl_3_, 400 MHz): δ 10.12 (s, 1H, 1NH), 9.54 (s, 1H, 1NH), 7.39 (d, 2H, *J* = 8.1 Hz, ArH), 7.31 (d, 4H, *J* = 8.1 Hz, ArH), 7.21(d, 2H, *J* = 8.1 Hz, ArH), 6.38 (d, 1H, *J* = 4.8 Hz, H5), 5.17 (d, 1H, *J* = 4.8 Hz, H4), 2.39 (s, 3H, OCH_3_). ^13^C-NMR (CDCl_3_, 100 MHz): δ 175.5 (CS), 141.5, 140.0, 134.5, 133.7, 129,128.0, 127.7, 125.7, 124.9 (ArC), 99.3 (NH—C=C), 55.5 (CHAr), 21.2 (CH_3_). GCMS *m/z* (rel. abundance): 314 (M.+, 100), 297 (13), 281 (34), 268 (26), 241 (54), 239 (43), 223 (63), 203 (30), 160 (19), 113 (19),100 (11). Anal. Calcd. for C_17_H_15_ClN_2_S (314.83); C, 64.85; H, 4.80; N, 8.90; S, 10.18. Found: C, 64.77; H, 5.01; N, 8.72; S, 10.33.

##### 6-(4-Bromophenyl)-4-(4-(dimethylamino)phenyl)-3,4-dihydropyrimidine-2(1H)-thione (**5e**)

Yield: 0.633 g, 68% (orange solid); m.p.112–3 °C; IR (KBr, cm^−1^) υ: 3195 (NH), 3084, 2925 (Ar-CH), 1679 (C=N), 1523, 1482 (C=C), 1174 (C=S). ^1^H-NMR (CDCl_3_, 400 MHz): δ 10.52 (s, 1H, 1NH), 9.76 (s, 1H, 1NH), 7.63 (d, 2H, *J* = 8.1 Hz, ArH), 7.57 (d, 2H, *J* = 8,1 Hz, ArH), 7.32 (d, 2H, *J* = 8.1 Hz, ArH), 7.22 (d, 2H, *J* = 8.1 Hz, ArH), 6.34 (d, 1H, *J* = 4.8 Hz, H5), 5.08 (d, 1H, *J* = 4.8 Hz, H4), 2.98 (s, 6H, NMe_2_). GCMS *m/z* (rel. abundance): 388 (M.+, 100), 372 (21), 355 (60), 332 (17), 301 (16), 268 (65), 232 (31), 186 (21), 157 (23), 120 (17). Anal. Calcd. for C_18_H_18_BrN_3_S (388.32); C, 55.67; H, 4.67; N, 10.32; S, 8.26. Found: C, 55.93; H, 4.72; N, 10.11; S, 8.25.

##### 6-(4-Chlorophenyl)-4-phenyl-3,4-dihydropyrimidine-2(1H)-thione (**5f**)

Yield: 0.384 g, 66% (white solid); m.p. 162–3 °C; IR (KBr, cm^−1^) υ: 3188 (NH), 3028, 2979 (Ar-CH), 1671 (C=N), 1569,1486 (C=C), 1190 (C=S). ^1^H-NMR (CDCl_3_, 400 MHz): δ 10.00 (s, 1H, 1NH), 9.67 (s, 1H, 1NH), 7.70–7.27 (m, 17H, ArH), 6.72 (d, 1H, *J* = 4.8 Hz, H5), 5.24 (d, 1H, *J* = 4.8 Hz, H4). GCMS *m/z* (rel. abundance): 300 (M.+, 67), 285 (40), 254 (48), 225 (100), 219 (55), 189 (32), 161 (49), 137 (31), 103 (14). Anal. Calcd. for C_16_H_13_ClN_2_S (300.81); C, 63.88; H, 4.36; N, 9.31; 10.66. Found: C, 64.06; H, 4.22; N, 10.41; S, 8.14.

##### 6-(4-Chlorophenyl)-4-(4-nitrophenyl)-3,4-dihydropyrimidine-2(1H)-thione (**5g**)

Yield: 0.589 g, 71% (grey solid); m.p. 210–12 °C; IR (KBr, cm^−1^) υ: 3155 (NH), 3070 (Ar-CH), 1678 (C=N), 1552,1493 (C=C), 1177 (C=S). ^1^H-NMR (CDCl_3_, 400 MHz): δ 10.28 (s, 1H, 1NH), 9.72 (s, 1H, 1NH), 8.30 (d, 2H, *J* = 8.1 Hz, ArH), 8.10 (d, 2H, J = 8.1 Hz, ArH), 7.59 (d, 2H, J = 8.1 Hz, ArH), 7.42 (d, 2H, *J* = 8.1 Hz, ArH), 6.43 (d, 1H, J = 4.8 Hz, H5), 5.26 (d, 1H, J = 4.8 Hz, H4). ^13^C-NMR (CDCl_3_, 100 MHz): δ 176.6 (CS), 144.5, 140.0, 135.0, 132.4, 1312.0, 130.0, 128.4, 126.2, 123.8 (ArC), 101.9 (NH-C=C), 52.0 (CHAr). GCMS *m/z* (rel. abundance): 345 (M.+, 100), 312 (50), 310 (6), 270 (26), 255 (9), 223 (16), 190 (9), 176 (16), 134 (16), 122 (28). Anal. Calcd. for C_16_H_12_ClN_3_O_2_S (345.80); C, 55.57; H, 3.50; N, 12.15; S, 9.27. Found: C, 55.33; H, 3.62; N, 12.23; S, 9.15.

##### 6-(4-Chlorophenyl)-4-(furan-2-yl)-3,4-dihydropyrimidine-2(1H)-thione (**5i**)

Yield: 0.509 g, 73% (yellow solid); m.p. 165–7 °C; IR (KBr, cm^−1^) υ: 3187 (NH), 3104, 2984 (Ar-CH), 1676 (C=N), 1571,1477 (C=C), 1186 (C=S). ^1^H-NMR (CDCl_3_, 400 MHz) δ: 10.08 (s, 1H, 1NH), 9.86 (s, 1H, 1NH), 7.69–7.39 (m, 7H, 4 ArH & 3 furyl-H), 6.23 (d, 1H, J = 4.8 Hz, H5), 5.35 (d, 1H, J = 4.8 Hz, H4). GCMS *m/z* (rel. abundance): 290 (M.+, 100), 257 (32), 235 (15), 215 (32), 190 (16), 179 (42), 159 (23), 131 (13), 115 (5). Anal. Calcd. for C_14_H_11_ClN_2_OS (290.77); C, 57.83; H, 3.81; N, 9.63; S, 11.03. Found: C, 58.14; H, 3.69; N, 9.48; S, 10.83. 

##### 6-(4-Aminophenyl)-4-(naphthalen-1-yl)-3,4-dihydropyrimidine-2(1H)-thione (**5j**)

Yield: 0.555 g, 73% (pale yellow solid); m.p. 155–6 °C; IR(KBr, cm^−1^) υ: 3217 (NH), 3051,2924 (Ar-CH), 1669 (C=N), 1559,1464 (C=C), 1177 (C=S). ^1^H-NMR (CDCl_3_, 400 MHz): δ 9.28 (s, 1H, 1NH), 9.23 (s, 1H, 1NH), 8.16 (d, 1H, *J* = 8.1 Hz, ArH), 7.88–7.53 (m, 6H, ArH), 7.43 (d, 2H, *J* = 8.1 Hz, ArH), 6.75 (d, 2H, *J* = 8.1 Hz, ArH), 6.68 (bs, 2H, NH_2_), 6.30 (d, 1H, *J* = 4.8 Hz, H5), 5.30 (d, 1H, *J* = 4.8 Hz, H4). GCMS *m/z* (rel. abundance): 331 (M.+, 100), 298 (50), 274 (43), 256 (23), 239 (15), 204 (23), 160 (17), 136 (16), 119 (8), 112 (10). Anal. Calcd. for C_20_H_17_N_3_S (331.43); C, 72.48; H, 5.17; N, 12.68; S, 9.67. Found: C, 72.22; H, 4.93; N, 12.53; S, 9.9.82.

#### 4.12.2. General Procedure for Synthesis of (4,6-diaryl-1,6-dihydropyrimidin-2-yl)-2-((1,3-dioxoisoindolin-2-yl)methyl)benzothioates **10a–j**

A solution of equimolar amounts of α-phthlimido-*o*-toluyl chloride (**9**, 0.668 mmol) and an appropriate 4,6-diaryl-3,4-dihydropyrimidine-2(*1H*)-thione **5a–j** (0.668 mmol) in 10 mL absolute dichloromethane was stirred at rt for 4–6 h. The progress of the reaction was monitored periodically by TLC using dichloromethane/ethyl acetate (9:1) as mobile phase. The reaction mixture was then treated with brine, extracted with ethyl acetate, treated with dil. hydrochloric acid, sod. bicarbonate and finally washed with water. The organic solvent was dried with anhydrous sodium sulfate and evaporated under vacuum. The solid product was purified by crystallization from the appropriate solvent to give the title hybrids **10a–j.**

##### S-(4,6-Diphenyl-1,6-dihydropyrimidin-2-yl)2-((1,3-dioxoisoindolin-2-yl)methyl)benzothioate (**10a**)

Yield: 0.233 g, 66% (white solid, ethanol); m.p. 147–8 °C; IR (KBr, cm^−1^) υ: 3399 (NH), 3071,3011 (Ar-CH), 1772,1716 (CO), 1603 (C=N), 1547,1491 (C=C). ^1^H-NMR (DMSO-d_6_, 400 MHz): δ 11.46 (s, 1H, NH), 7.90–721 (m, 18 H, ArH), 6.42 (d, 1H, *J* = 4.8 Hz, CH=C-Ar), 5.12 (d, 1H, *J* = 4.8 Hz, CHAr), 5.06 (s, 2H, CH_2_). ^13^C-NMR (DMSO-d_6_, 100 MHz): δ 189.7 (CO), 168.3 (2CO), 156.4, 144.0, 140.5, 136.0, 136.7, 135.5, 135.0, 132.0, 132.6, 131.4, 129.3, 128.4, 127.6, 127.0, 126.7, 126.3, 124.6, 124.0 (ArC), 107.8 (C=CH-Ar), 55.1 (CHAr). (GCMS *m/z* (rel. abundance): 529 (M.+, 36), 452 (29), 392 (43), 338 (20), 280 (100), 265 (60), 236 (20), 223 (10), 173 (35), 146 (28). Anal. Calcd for C_32_H_23_N_3_O_3_S (529.61); C, 72.57; H, 4.38; N, 7.93; S, 6.05. Found: C, 72.34; H, 4.30; N, 8.07; S, 6.23.

##### S-(4-(4-Chlorophenyl)-6-(4-methoxyphenyl)-1,6-dihydropyrimidin-2-yl)2-((1,3-dioxoisoindolin-2-yl)methyl)benzothioate (**10b**)

Yield: 0.278 g, 70% (pale yellow solid, ethanol); m.p. 142–3 °C; IR (KBr, cm^−1^) υ: 3450 (NH), 3064,2918 (Ar-CH), 1772, 1725 (CO), 1676 (C=N), 1594,1489 (C=C). ^1^H-NMR (DMSO-d_6_, 400 MHz): δ 11.49 (s, 1H, NH), 7.99–6.92 (m, 16 H, ArH), 6.28 (d, 1H, *J*= 4.8 Hz, CH=C-Ar), 5.20 (d, 1H, *J* = 4.8 Hz, CHAr), 5.08 (s, 2H, CH_2_), 3.76 (s, 3H, OCH_3_). ^13^C-NMR (DMSO-d_6_, 100 MHz): δ 188.5 (CO), 168.3 (2CO), 159.4, 145.0, 138.3, 137.8, 136.1, 135.6, 135.9, 134.3, 133.7, 132.8, 132.2, 131.5, 129.3, 129.0, 128.5, 128.3, 127.3, 124.5, 120.6, 114.9 (ArC), 111.4 (C=CH-CHAr), 55.5 (CHAr). GCMS *m/z* (rel. abundance): 593 (M.+, 100), 562 (50), 462 (34), 382 (43), 355 (61), 329 (23), 264 (32), 186 (26), 160 (35), 132 (20). Anal. Calcd. for C_33_H_24_ClN_3_O_4_S (594.08); C, 66.72; H, 4.07; N, 7.07; S, 5.40. Found: C, 66.39; H, 4.22; N, 8.11; S, 5.91.

##### S-(4,6-Bis(4-chlorophenyl)-1,6-dihydropyrimidin-2-yl)2-((1,3-dioxoisoindolin-2-yl)methyl)benzothioate (**10c**)

Yield: 0.258 g, 65% (pale yellow solid, ethanol/chloroform); m.p. 118–19 °C; IR (KBr, cm^−1^) υ: 3432 (NH), 3064, 2964 (Ar-CH), 1772,1616 (CO), 1672 (C=N), 1592, 1489 (C=C). ^1^H NMR (DMSO-d_6_, 400 MHz): δ 11.43 (s, 1H, NH), 7.92–7.19 (m, 16H, ArH), 6.42 (d, 1H, *J* = 4.8 Hz, CH=C-Ar), 5.19 (d,1H, *J* = 4.8 Hz, CHAr), 5.06 (s, 2H, CH_2_). GCMS *m/z* (rel. abundance): 598 (M.+, 100), 487 (86), 466 (20), 568 (37)521 (17), 398 (28), 358 (18), 302 (42), 266 (59), 275 (27), 190 (19), 160 (30), 147 (46), 132 (23). Anal. Calcd. for C_32_H_21_Cl_2_N_3_O_3_S (598.50); C, 64.22; H, 3.54; N, 7.02; S, 5.36. Found: C, 64.00; H, 3.3.78; N, 7.21; S, 5.30.

##### S-(6-(4-Chlorophenyl)-4-(p-tolyl)-1,6-dihydropyrimidin-2-yl)2-((1,3-dioxoisoindolin-2-yl)methyl)benzothioate (**10d**)

Yield: 0.282 g, 73% (yellow solid, ethanol); m.p. 144–6 °C; IR(KBr, cm^−1^) υ: 3413 (NH), 3061,3041, 2922 (Ar-CH), 1771,1715 (CO), 1677 (C=N), 1545, 1490 (C=C). ^1^H-NMR (DMSO-d_6_, 400 MHz): δ 11.45 (s, 1H, 1NH), 7.89–7.18 (m, 16H, ArH), 6.34 (d,1H, *J* = 4.8 Hz, CH=C-Ar), 5.20 (d,1H, *J* = 4.8 Hz, CH-Ar), 5.08 (s, 2H, CH_2_), 2.34 (s, 3H, CH_3_). GCMS *m/z* (rel. abundance): 577.5 (M.+, 31), 487 (70), 466 (100), 457 (20), 385 (30), 375 (21), 314 (39), 297 (81), 264 (57), 217 (10), 160 (26), 146 (22), 132 (16). Anal. Calcd. for C_33_H_24_ClN_3_O_3_S (578.08); C, 68.56; H, 4.18; N, 7.27; S, 5.55. Found: C, 68.84; H, 4.01; N, 7.37; S, 5.23.

##### S-(4-(4-Bromophenyl)-6-(4-(dimethylamino)phenyl)-1,6-dihydropyrimidin-2-yl) 2-((1,3-dioxoisoindolin-2-yl)methyl)benzothioate (**10e**)

Yield: 0.304 g, 70% (brown solid, ethanol/chloroform); m.p. 135–6 °C; IR (KBr, cm^−1^) υ: 3476 (NH), 2920 (Ar-CH), 1770,1715 (CO), 1674 (C=N), 1578,1485(C=C). ^1^H-NMR (DMSO-d_6_, 400 MHz): δ 11.45 (s, 1H, NH), 8.08–6.75 (m, 16H, ArH), 6.34 (d, 1H, *J* = 4.8 Hz, CH=C-Ar), 5.18 (d, 1H, *J* = 4.8 Hz, CHAr), 5.05 (s,2H, CH_2_), 2.52 (s, 6H, NMe_2_). GCMS *m/z* (rel. abundance): 651 (M.+, 41), 569 (19), 546 (100), 531 (13), 519 (18), 415 (21), 362 (49), 302 (36), 264 (30), 236 (18), 202 (12), 146 (16), 132 (24). Anal. Calcd. for C_34_H_27_BrN_4_O_3_S (651.57); C, 62.67; H, 4.18; N, 8.60; S, 4.92. Found: C, 62.93; H, 4.28; N, 8.44; S, 4.88.

##### S-(4-(4-Chlorophenyl)-6-phenyl-1,6-dihydropyrimidin-2-yl)2-((1,3-dioxoisoindolin-2-yl)methyl)benzothioate (**10f**)

Yield: 0.282 g, 75% (pale yellow solid, ethanol); m.p. 143–4 °C; IR (KBr, cm^−1^) υ: 3464 (NH), 3061, 2970 (Ar-CH), 1772,1716 (CO), 1677 (C=N), 1557,1490 (C=C). ^1^H-NMR (DMSO-d_6_, 400 MHz): δ 11.42 {s, 1H, NH), 8.18–7.15 (m, 17H, ArH), 6.45 (d, 1H, *J* = 4.8 Hz, CH=C-Ar), 5.16 (d, 1H, *J* = 4.8 Hz, CH-Ar), 5.01 (s,2H, CH_2_). GCMS *m/z* (rel. abundance); 563.5 (M.+, 100), 543 (48), 443 (43), 414 (38), 389 (18), 375 (36), 300 (28), 265 (31), 227 (24), 161 (36), 146 (27), 132 (17). Anal. Calcd. for C_32_H_22_ClN_3_O_3_S (564.05); C, 68.14; H, 3.93; N, 7.45; S, 5.68. Found: C, 67.83; H, 4.17; N, 7.27; S, 5.55.

##### S-(4-(4-Chlorophenyl)-6-(4-nitrophenyl)-1,6-dihydropyrimidin-2-yl)2-((1,3-dioxoisoindolin-2-yl)methyl)benzothioate (**10g**)

Yield: 0.280 g, 69% (brown solid, ethanol/chloroform); m.p.161–3 °C; IR (KBr, cm^−1^) υ: 3418 (NH), 3030,2926 (Ar-CH), 1774,1715 (CO), 1623 (C=N), 1521 (C=C). ^1^H-NMR (DMSO-d_6_, 400 MHz): δ 11.52 (s, 1H, NH), 8.51–7.15 (m, 16H, ArH), 6.39 (d, 1H, *J* = 4.8 Hz, CH=C-Ar), 5.21 (d, 1H, *J* = 4.8 Hz, CHAr), 5.02 (s, 2H, CH_2_). ^13^C-NMR (DMSO-d_6_, 100 MHz): δ 188.4 (CO), 168.3 (2CO), 157.5, 148.0, 146.1, 142.0, 137.9, 137.7, 135.3, 135.0, 133.7, 132.5, 131.8, 129.8, 129.0, 128.1, 127.4, 126.1, 125.1, 124.6, 124.5 (ArC), 112.0 (C=CH—CHAr), 45.2 (CHAr). GCMS *m/z* (rel. abundance): 608.5 (M.+, 50), 551 (17), 532 (25), 504 (55), 446 (31), 373 (100), 346 (22), 311 (25), 306 (9), 264 (39), 232 (21), 188 (31), 163 (16). Anal. Calcd. for C_32_H_21_ClN_4_O_5_S (609.05); C, 63.11; H, 3.48; N, 9.20; S, 5.26. Found: C, 62.88; H, 3.59; N, 9.18; S, 5.17.

##### S-(4-(4-Aminophenyl)-6-(4-chlorophenyl)-1,6-dihydropyrimidin-2-yl)2-((1,3-dioxoisoindolin-2-yl)methyl)benzothioate (**10h**)

Yield: 0.285 g, 74% (orange solid, ethanol/chloroform); m.p. 185–7 °C; IR(KBr, cm^−1^) υ: 3430 (NH), 3060,2942 (Ar-CH), 1772,1716 (CO), 1677 (C=N), 1545,1490 (C=C). ^1^H-NMR (DMSO-d_6_, 400 MHz): δ 11.48 (s, 1H, NH), 8.10–6.98 (m, 16H, ArH), 6.68 (d,1H, *J* = 4.8 Hz, CH=C-Ar), 6.19 (bs, 2H, NH_2_), 5.23 (d, 1H, *J* = 4.8 Hz, CHAr), 5.03 (s, 2H, CH_2_). GCMS *m/z* (rel. abundance): 578.5 (M.+, 47), 514 (9), 432 (22), 394 (11), 342 (100), 312 (49), 282 (46), 264 (34), 238 (27), 196 (17), 140 (14), 132 (11). Anal. Calcd. for C_32_H_23_ClN_4_O_4_S (579.07); C, 66.37; H, 4.00; N, 9.68; S, 5.54. Found: C, 66.20; H, 3.83; N, 9.83; S, 5.50. 

##### S-(4-(4-Chlorophenyl)-6-(furan-2-yl)-1,6-dihydropyrimidin-2-yl)2-((1,3-dioxoisoindolin-2-yl)methyl)benzothioate (**10i**)

Yield: 0.274 g, 74% (grey solid, ethanol); m.p. 146–8 °C; IR (KBr, cm^−1^) υ: 3464 (NH), 3064,2928 (Ar-CH), 1770,1716 (CO), 1666 (C=N), 1502 (C=C). ^1^H-NMR (DMSO-d_6_, 400 MHz): δ 11.19 (s,1H, NH), 7.92–7.09 (m, 12 ArH and 3 furanyl-H), 6.25 (d, 1H, *J* = 4.8 Hz, CH=C-Ar), 5.21 (d, 1H, *J* = 4.8 Hz, CHAr), 5.02 (s, 2H, CH_2_). GCMS *m/z* (rel. abundance): 553.5 (M.+, 100), 511 (11), 449 (41), 393 (20), 315 (44), 287 (18), 254 (23), 238 (26), 264 (20), 160 (21), 132 (16). Anal. Calcd. for C_30_H_20_ClN_3_O_4_S (554.02); C, 65.04; H, 3.64; N, 7.58; S, 5.79. Found: C, 65.33; H, 3.78; N, 7.42; S, 5.51.

##### S-(4-(4-Aminophenyl)-6-(naphthalen-1-yl)-1,6-dihydropyrimidin-2-yl)2-((1,3-dioxoisoindolin-2-yl)methyl)benzothioate (**10j**)

Yield: 0.287 g, 74% (orange solid, ethanol); m.p. 176–8 °C; IR (KBr, cm^−1^) υ: 3384 (NH), 3062,2975 (Ar-CH), 1771,1714 (CO), 1667 (C=N), 1598,1467 (C=C). ^1^H-NMR (DMSO-d_6_, 400 MHz): δ 11.52 (s, 1H, NH), 8.50–7.12 (m, 18H, ArH), 6.58 (d, 1H, J = 4.8 Hz, CH=C-Ar), 6.17 (bs, 2H, NH_2_), 5.23 (d, 1H, J = 4.8 Hz, CHAr), 5.05 (s, 2H, CH_2_). ^13^C-NMR (DMSO-d_6_, 100 MHz): δ 188.1 (CO), 167.5 (2CO), 157.5, 145.3, 143.1, 137, 136.6, 135.8, 135.5, 135.3, 134.3, 134.0, 133.5, 132.5,132.3, 130.8, 129.7, 129.1, 128.9, 128.6, 127.8, 126.0, 125.5, 125.0, 124.0, 123.5 (ArC),113.3 (C=CH-Ar), 55.0 (CHAr). GCMS *m/z* (rel. abundance): 594 (M.+, 42), 475 (28), 434 (16), 358 (100), 330 (29), 317 (10), 264 (28), 232 (11), 127 (15), 133 (27), 104 (9). Anal. Calcd. for C_36_H_26_N_4_O_3_S (594.68); C, 72.71; H, 4.41; N, 9.42; S, 5.39. Found: C, 72.63; H, 4.62; N, 9.65; S, 5.23.

## 5. Conclusions

A novel series of dihydropyrimidine phthalimide hybrids was synthesized, characterized and evaluated for their DPP-4 inhibitory activity, as well as, antioxidant activity. Hybrids **10g, 10i, 10e, 10d** and **10b** showed superior DPP-4 inhibitory activity compared to alogliptin, in the in vitro DPP-4 inhibition assay. Additionally, hybrids **10g** and **10i** have the most potent and longest DPP-4 inhibitory activity among the synthesized hybrids and alogliptin, in the in vivo study that carried on SD rats. Furthermore, investigation of antihyperglycemic effect of our synthesized hybrids in HFD-induced type 2 diabetic rats, revealed that hybrids **10g**, **10i** and **10e** exhibited better glycemic control than alogliptin; this effect was further potentiated by addition of MET. Notably, glucose tolerance reached a normal level in 10g/MET treated group. Finally, hybrids **10j**, **10e**, **10h** and **10d** showed potent radical scavenging activity in DPPH assay. 

## Figures and Tables

**Table 1:** DPP-4, DPP-8 and DPP-9 inhibitory activities of hybrids **10a–j** and alogliptin.

Compound No.	DPP-4 IC_50_ (nM) *	DPP-8 IC_50_ (µM) *	DPP-9 IC_50_ (µM) *
**10a**	19.74 ± 1.27	>100	>100
**10b**	3.78 ± 0.23	>100	>100
**10c**	14.71 ± 0.82	>100	>100
**10d**	2.19 ± 0.14	>100	>100
**10e**	1.42 ± 0.11	>100	>100
**10f**	31.52 ± 2.41	>100	>100
**10g**	0.51 ± 0.03	>100	>100
**10h**	10.37 ± 0.54	>100	>100
**10i**	0.66 ± 0.04	>100	>100
**10j**	7.35 ± 0.36	>100	>100
**Alogliptin**	5.71 ± 0.21	>100	>100

* Data are means of four replicates with standard error (Mean ± SE).

**Table 2:** Antioxidant activity of hybrids **10a–j** using the DPPH method.

Compound No. (100 µM)	% DPPH Scavenging *
**10a**	73.85 ± 2.46
**10b**	74.28 ± 1.69
**10c**	52.39 ± 0.93
**10d**	80.16 ± 1.38
**10e**	87.21 ± 2.58
**10f**	57.63 ± 2.14
**10g**	49.47 ± 1.08
**10h**	85.74 ± 2.76
**10i**	69.11 ± 1.44
**10j**	93.52 ± 1.65

* Data are means of four replicates with standard error (Mean ± SE).

**Table 3:** Types of binding interactions and energy scores (kcal/mol) for hybrids **10a–j** and alogliptin at the DPP-4 active site.

Compound No.	Types of Interactions	Energy Scores
**10a**	- One hydrogen bonding interaction with Arg 125 (3.14 Å).	−8.68
**10b**	- One π-cation interaction with Lys 554 (4.06 Å) - One π-hydrogen bonding interaction with Trp 629 (4.23 Å).	−9.12
**10c**	- Two hydrogen bonding interaction with Arg 125 and Tyr 547 (3.18 Å, 2.77 Å). - One π-hydrogen bonding interaction with His 126 (4.03 Å).	−7.74
**10d**	- Three hydrogen bonding interaction with Arg 125 (2.85 Å, 3.01 Å, 3.22 Å). - One hydrogen bonding interaction with Tyr 457 (2.71 Å). - One π-hydrogen bonding interaction with Tyr 547 (4.20 Å).	−8.14
**10e**	-Three hydrogen bonding interaction with Arg 125 (2.83 Å, 3.01 Å, 3.24 Å). - One hydrogen bonding interaction with Tyr 547 (2.73 Å).	−8.15
**10f**	- One hydrogen bonding interaction with Asp 545 (3.14 Å). - One π-cation interaction with Lys 554 (4.87 Å).	−7.74
**10g**	- Two hydrogen bonding interaction with Lys 554 and Glu 206 (2.97 Å and 3.09 Å).	−9.45
**10h**	- Two hydrogen bonding interaction with ASP 545 and VAL 546 (3.07 Å and 2.83 Å).	−9.32
**10i**	- Two hydrogen bonding interaction with Tyr 547 and Glu 206 (2.89 Å and 3.63 Å). - Two hydrogen bonding interaction with Arg 125 (3.34 Å and 2.91 Å).	−8.50
**10j**	- Three hydrogen bonding interaction with Glu 205, Glu 206 and Tyr 631 (3.16 Å, 3.12 Å and 3.53 Å). - One H-π interaction with Trp 629 (4.33 Å). - One π-H interaction with Tyr 456 (4.21 Å).	−7.72
**Alogliptin**	- Five hydrogen bonding interaction with Glu 205, Glu 206, Tyr 631, Arg 125 (2.78 Å and 2.96 Å, 2.90 Å, 3.35 Å and 2.97 Å). - Three ionic bond with Glu 206 and Glu 205 (2.96 Å, 2.90 Å and 3.95 Å).	−7.92

## Data Availability

The data presented in this study are openly available at doi.

## Supplementary Materials

IR spectroscopy, ^1^H-NMR, ^13^C^-^NMR and mass spectroscopy of all the synthesized compounds are available online at https://www.mdpi.com/1424-8247/14/2/144/s1, Figure S1: IR spectrum of compound **5a**, Figure S2: IR Spectroscopy of compound **5b**, Figure S3: IR Spectroscopy of compound **5c**, Figure S4: IR Spectroscopy of compound **5d**, Figure S5: IR Spectroscopy of compound **5e**, Figure S6: IR Spectroscopy of compound **5f**, Figure S7: IR Spectroscopy of compound **5g**, Figure S8: IR Spectroscopy of compound **5h**, Figure S9: IR Spectroscopy of compound **5i**, Figure S10: IR Spectroscopy of compound **5j**, Figure S11: IR Spectroscopy of compound **10a**, Figure S12: IR Spectroscopy of compound **10b**, Figure S13: IR Spectroscopy of compound **10c**, Figure S14: IR Spectroscopy of compound **10d**, Figure S15: IR Spectroscopy of compound **10e**, Figure S16: IR Spectroscopy of compound **10f**, Figure S17: IR Spectroscopy of compound **10g**, Figure S18: IR Spectroscopy of compound **10h**, Figure S19: IR Spectroscopy of compound **10i**, Figure S20: IR Spectroscopy of compound **10j**, Figure S21: ^1^H NMR Spectroscopy of Compound **5b**, Figure S22: ^1^H-NMR spectrum of compound **5c**, Figure S23: ^1^H NMR Spectroscopy of Compound **5d**, Figure S24: ^1^H NMR Spectroscopy of Compound **5e**, Figure S25: ^1^H NMR Spectroscopy of Compound **5f**, Figure S26: ^1^H NMR Spectroscopy of Compound **5g**, Figure S27: ^1^H NMR Spectroscopy of Compound **5h**, Figure S28: ^1^H NMR Spectroscopy of Compound **5i**, Figure S29: ^1^H NMR Spectroscopy of Compound **5j**, Figure S30: ^1^H NMR Spectroscopy of Compound **10a**, Figure S31: ^1^H NMR Spectroscopy of Compound **10b**, Figure S32: ^1^H NMR Spectroscopy of Compound **10c**, Figure S33: ^1^H NMR Spectroscopy of Compound **10d**, Figure S34: ^1^H NMR Spectroscopy of Compound **10e**, Figure S35: ^1^H NMR Spectroscopy of Compound **10f**, Figure S36: ^1^H NMR Spectroscopy of Compound **10g**, Figure S37: ^1^H NMR Spectroscopy of Compound **10h**, Figure S38: ^1^H NMR Spectroscopy of Compound **10i**, Figure S39: ^1^H NMR Spectroscopy of Compound **10j**, Figure S40: ^13^C NMR Spectroscopy of Compound **5a**, Figure S41: ^13^C NMR Spectroscopy of Compound **5d**, Figure S42: ^13^C NMR Spectroscopy of Compound **5g**, Figure S43: ^13^C NMR Spectroscopy of Compound **5h**, Figure S44: ^13^C NMR Spectroscopy of Compound **10a**, Figure S45: ^13^C NMR Spectroscopy of Compound **10b**, Figure S46: ^13^C NMR Spectroscopy of Compound **10d**, Figure S47: ^13^C NMR Spectroscopy of Compound **10g**, Figure S48: ^13^C NMR Spectroscopy of Compound **10j**, Figure S49: Mass Spectroscopy of Compound **5b**, Figure S50: Mass Spectroscopy of Compound **5c**, Figure S51: Mass Spectroscopy of Compound **5d**, Figure S52: Mass Spectroscopy of Compound **5e**, Figure S53: Mass Spectroscopy of Compound **5f**, Figure S54: Mass Spectroscopy of Compound **5g**, Figure S55: Mass Spectroscopy of Compound **5h**, Figure S56: Mass Spectroscopy of Compound **5i**, Figure S57: Mass Spectroscopy of Compound **5j**, Figure S58: Mass Spectroscopy of Compound **10a**, Figure S59: Mass Spectroscopy of Compound **10b**, Figure S60: Mass Spectroscopy of Compound **10c**, Figure S61: Mass Spectroscopy of Compound **10d**, Figure S62: Mass Spectroscopy of Compound **10e**, Figure S63: Mass Spectroscopy of Compound **10f**, Figure S64: Mass Spectroscopy of Compound **10g**, Figure S65: Mass Spectroscopy of Compound **10h**, Figure S66: Mass Spectroscopy of Compound **10i**, Figure S67: Mass Spectroscopy of Compound **10j**.
