# Supplementary material for: Novel Potent and Selective DPP-4 Inhibitors: Design, Synthesis and Molecular Docking Study of Dihydropyrimidine Phthalimide Hybrids
Source: Pharmaceuticals (Basel). 2021 Feb 11;14(2):144. doi: 10.3390/ph14020144 (PMC7918823; doi:10.3390/ph14020144)

IR Spectroscopy:

Figure S1: IR Spectroscopy of Compound **5a**

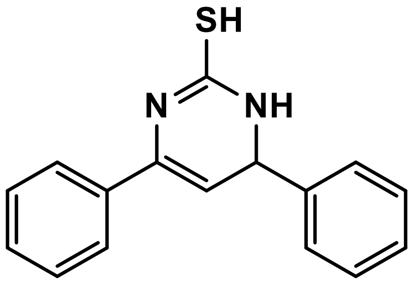

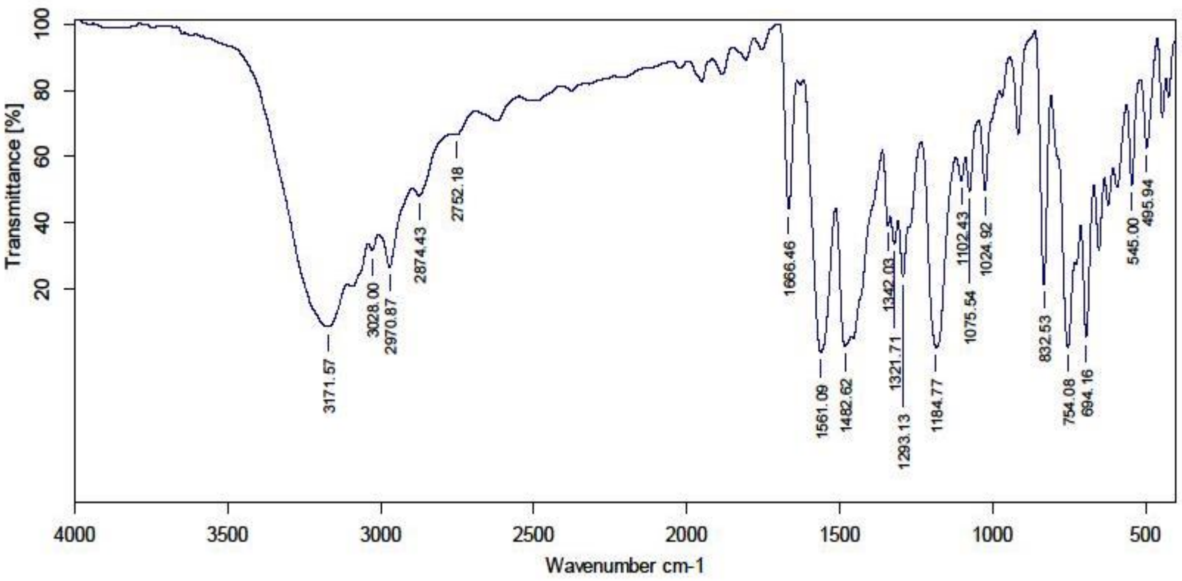

212-TRANSt.0 **01:33:42 ä** **11/04/2020**

Figure S2: IR Spectroscopy of Compound **5b**

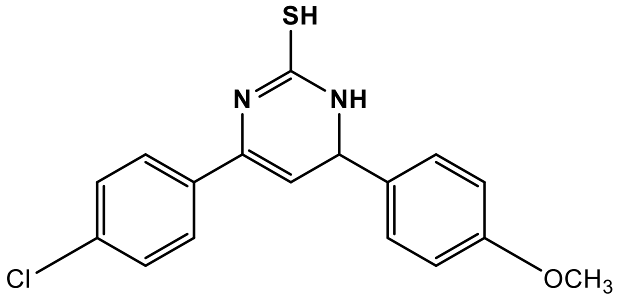

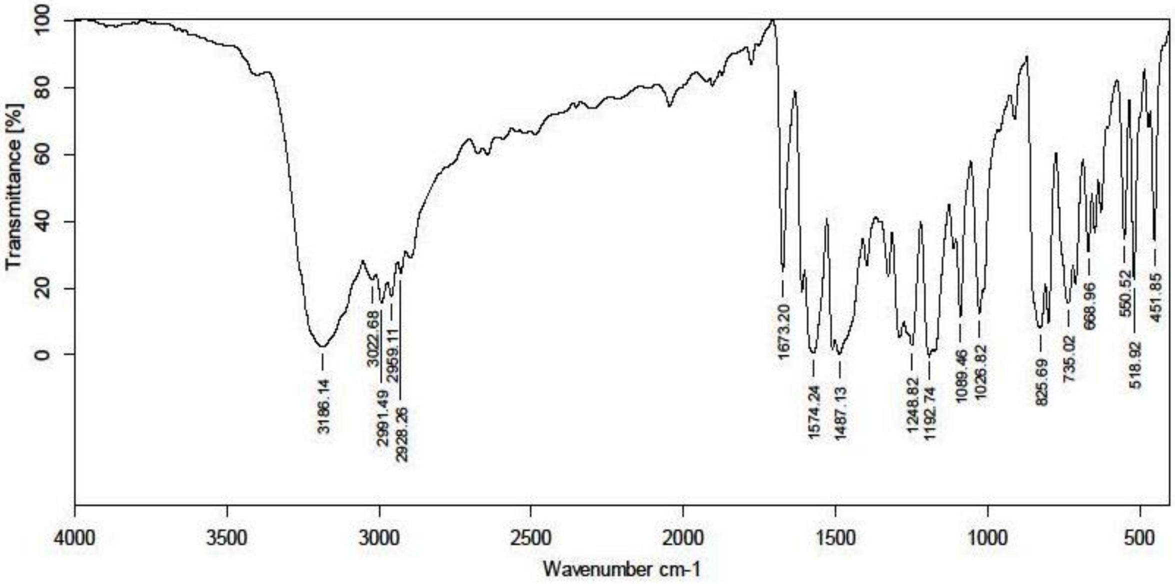

222-TRANSt.0 **01:38:59 ä** **11/04/2020**

Figure S3: IR Spectroscopy of Compound **5c**

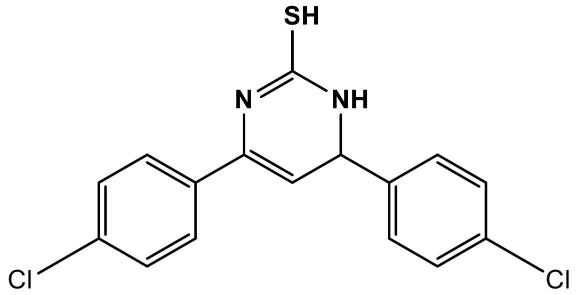

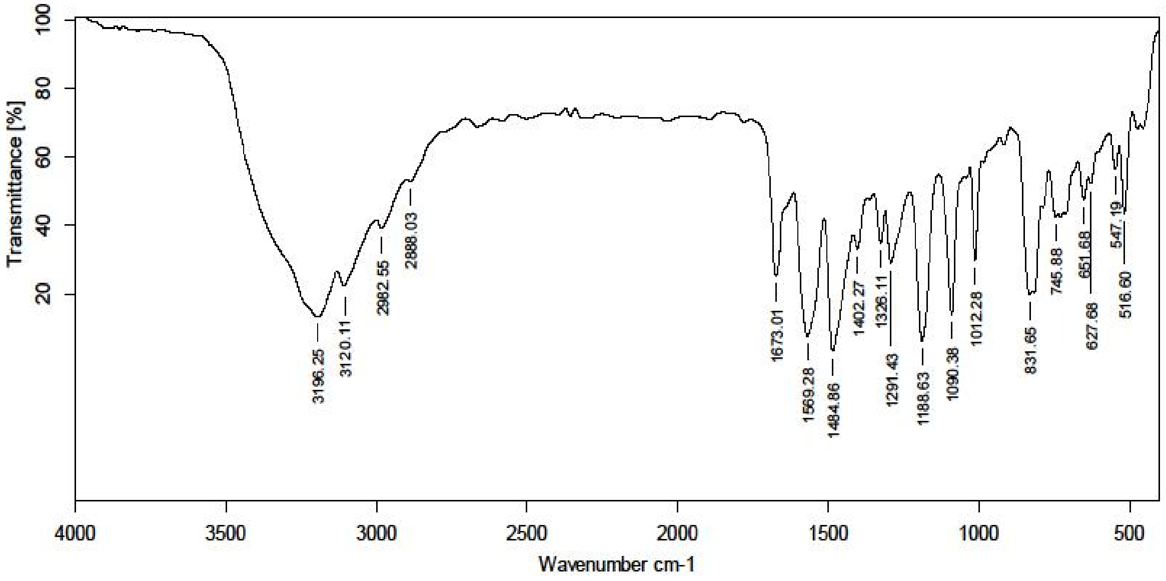

24-TRANSt.0 12:30:28 ä 11/04/2020

Figure S4: IR Spectroscopy of Compound **5d**

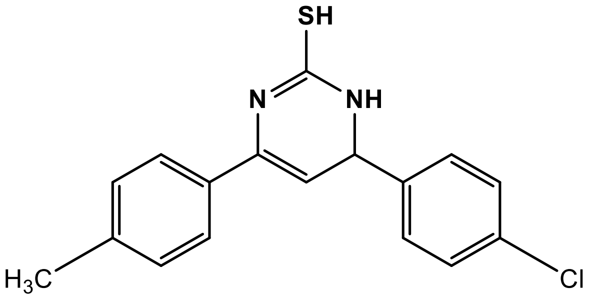

BRUKER

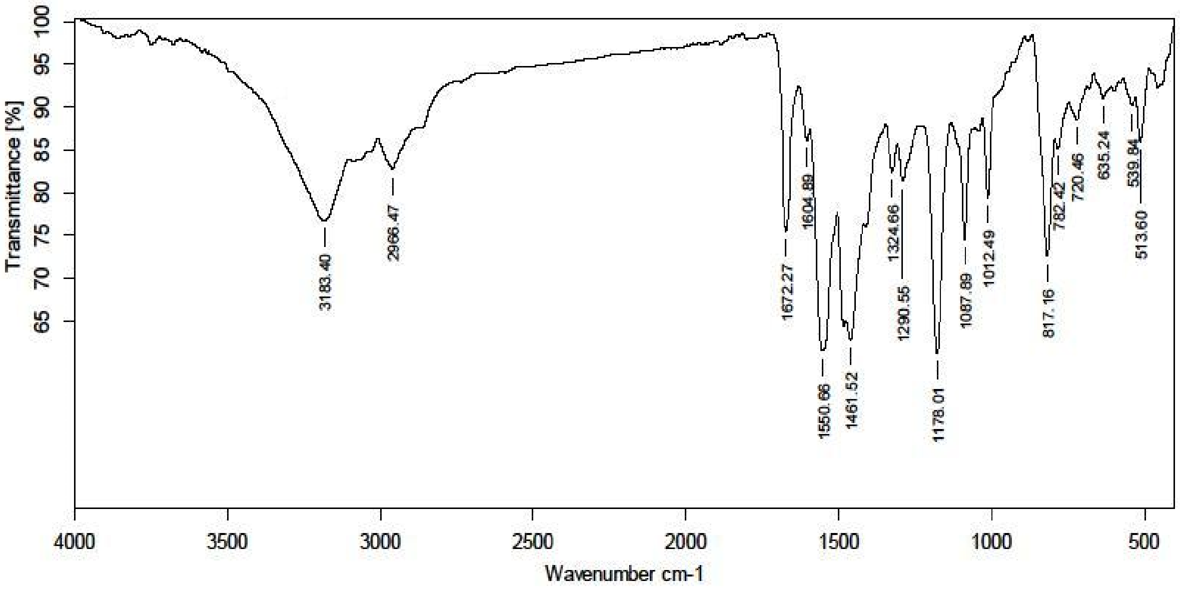

26-TRANSt.0 01:10:33 ä 11/04/2020

Figure S5: IR Spectroscopy of Compound **5e**

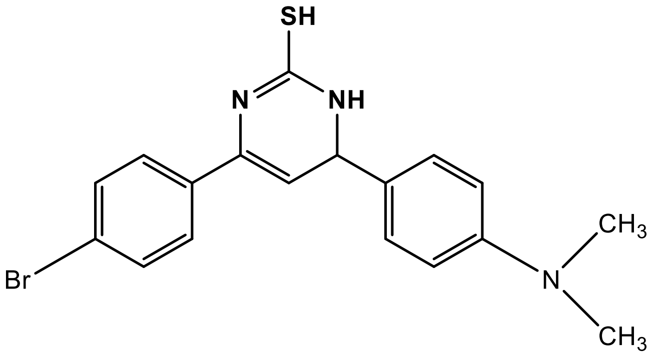

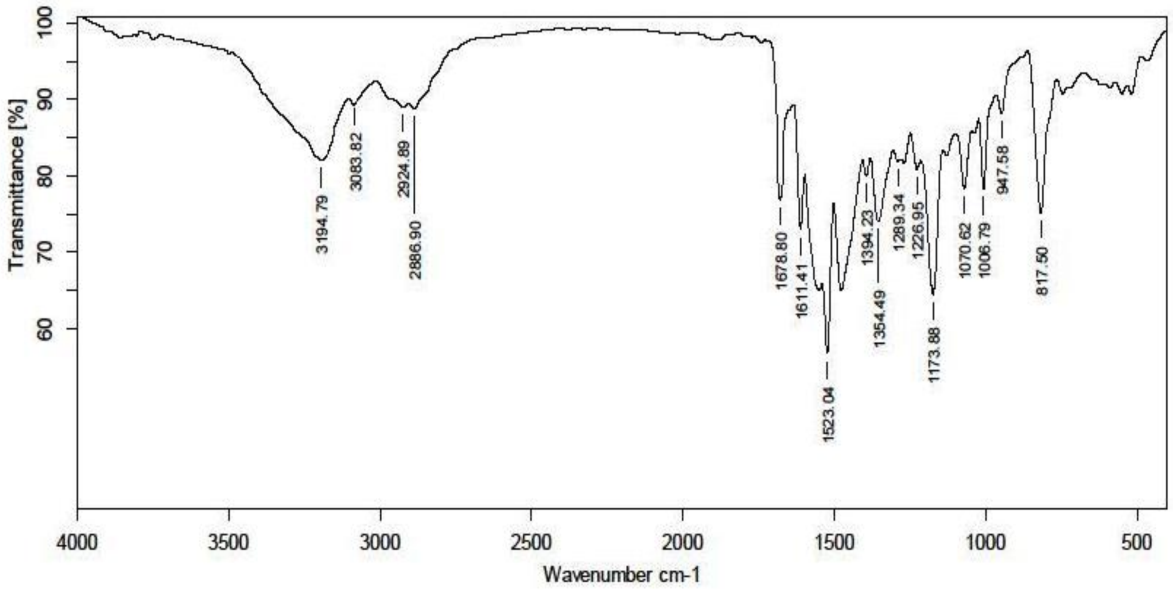

28-TRANSt.0 01:05:56 ã 11/04/2020

Figure S6: IR Spectroscopy of Compound **5f**

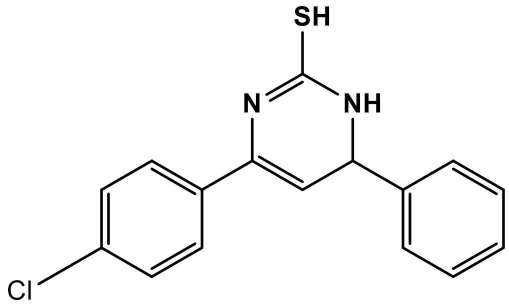

BRUKER

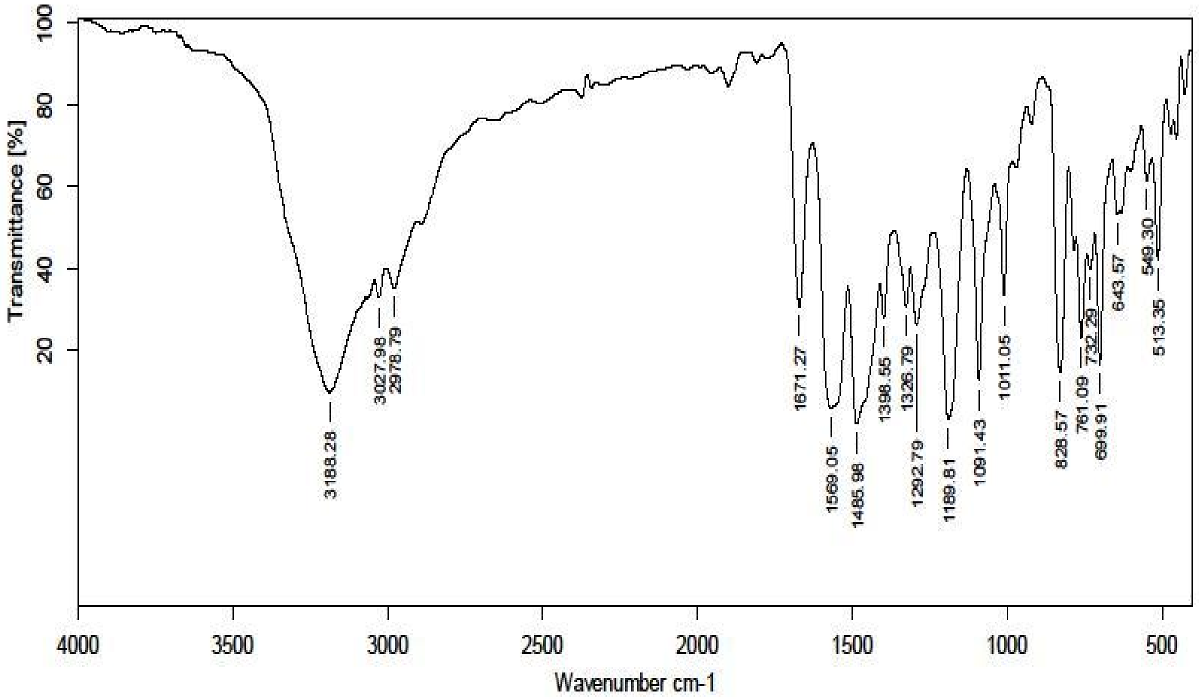

31-TRANSt.0 12:44:25 ä 11/04/2020

Figure S7: IR Spectroscopy of Compound **5g**

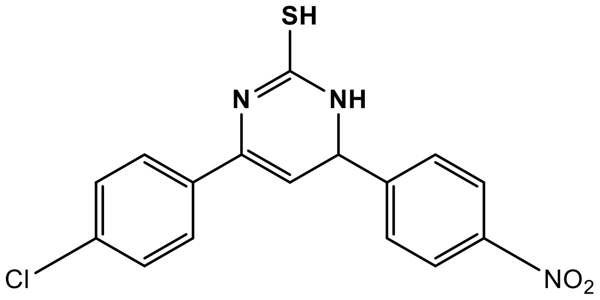

BRUKER

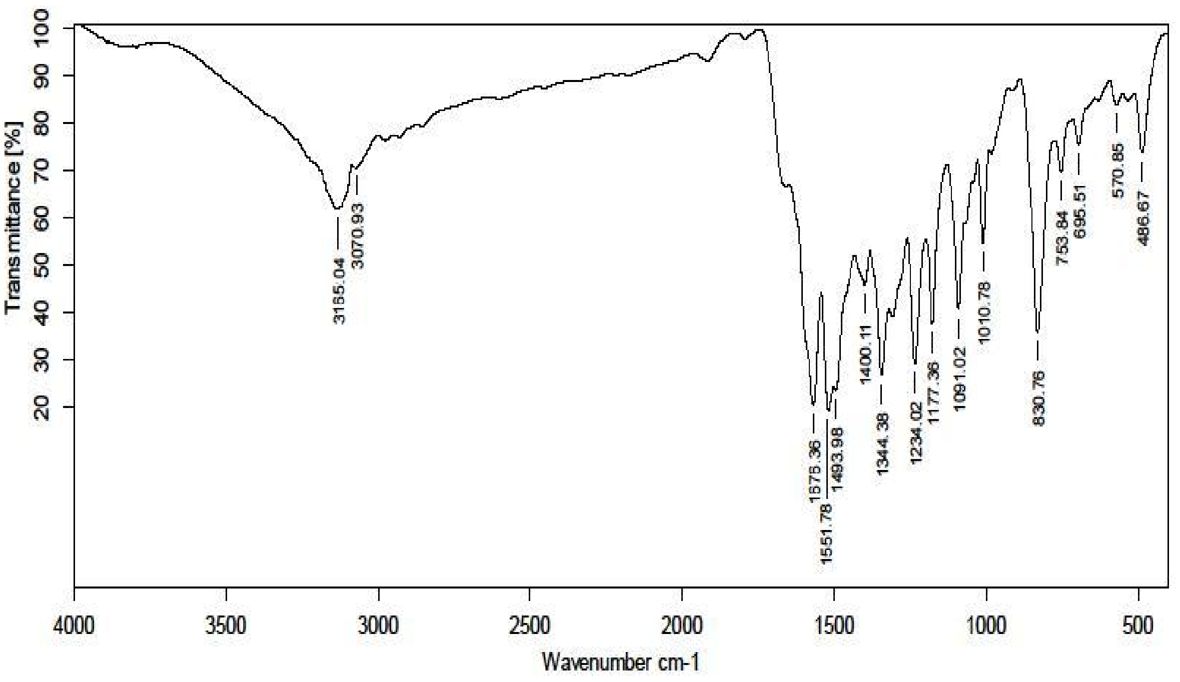

Figure S8: IR Spectroscopy of Compound **5h**

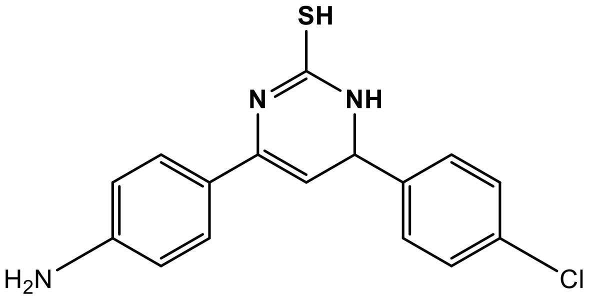

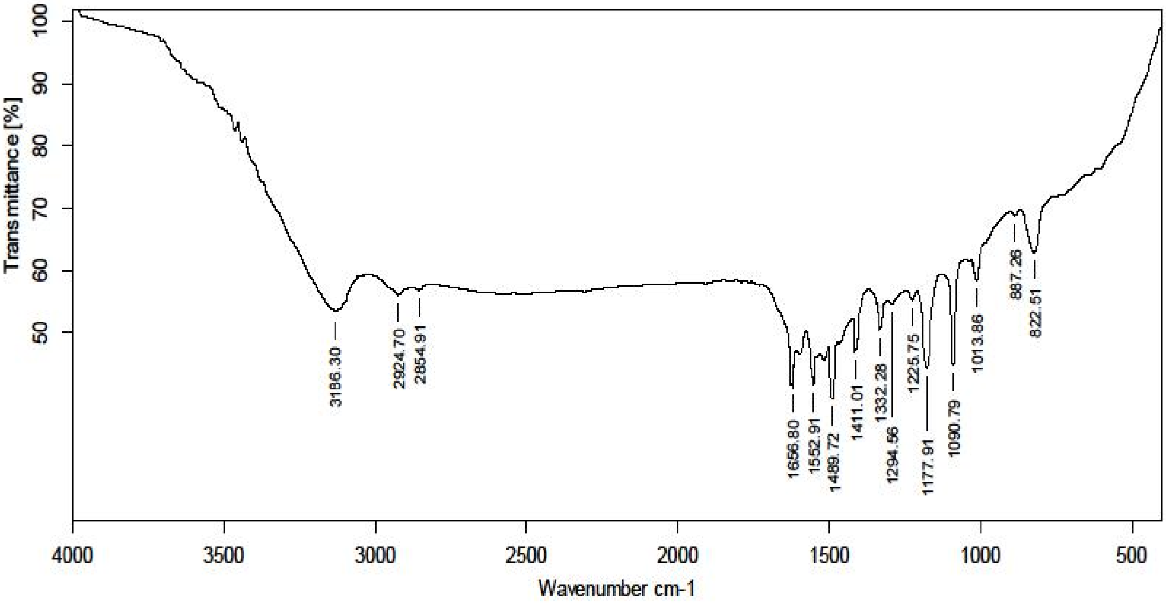

103-TRANSt.0 01:17:11 ä 11/04/2020

Figure S9: IR Spectroscopy of Compound **5i**

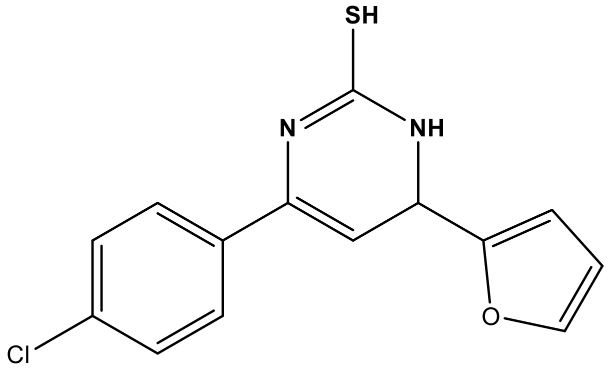

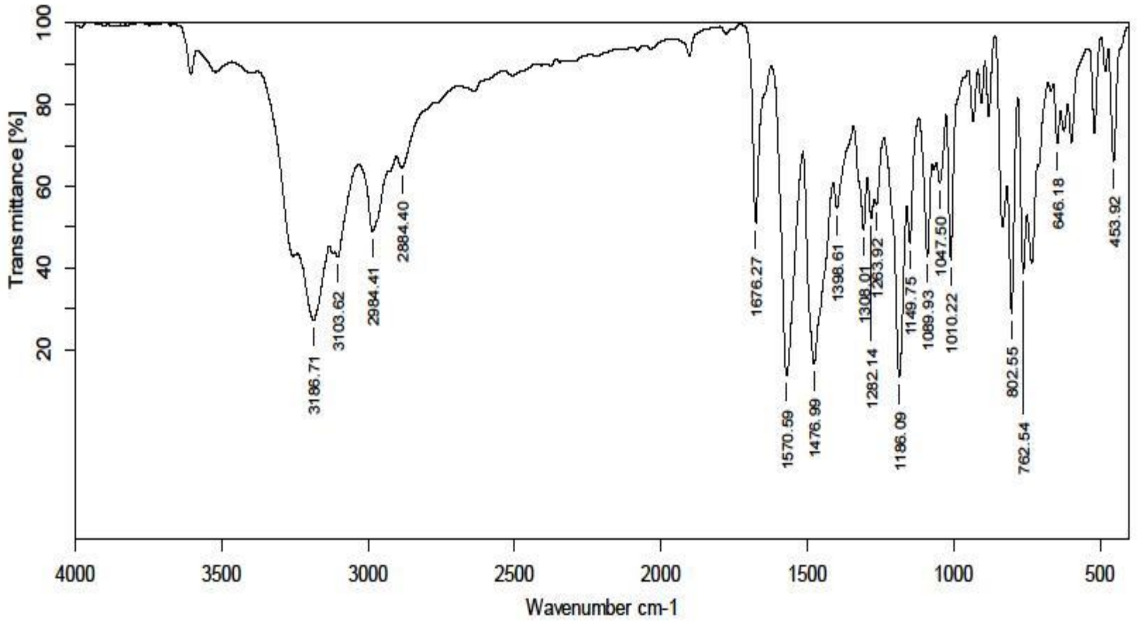

25-TRANSt.0 12:56:39 ä 11/04/2020

Figure S10: IR Spectroscopy of Compound **5j**

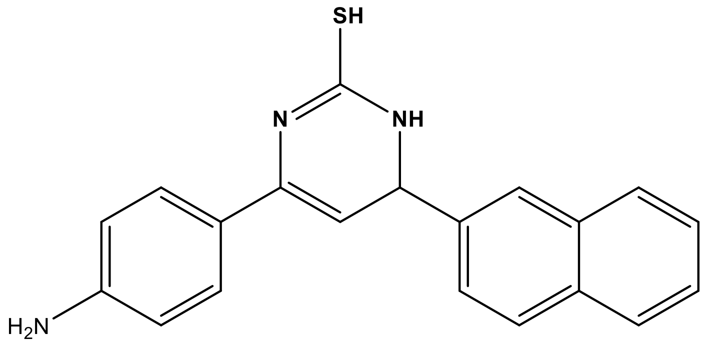

BRUKER

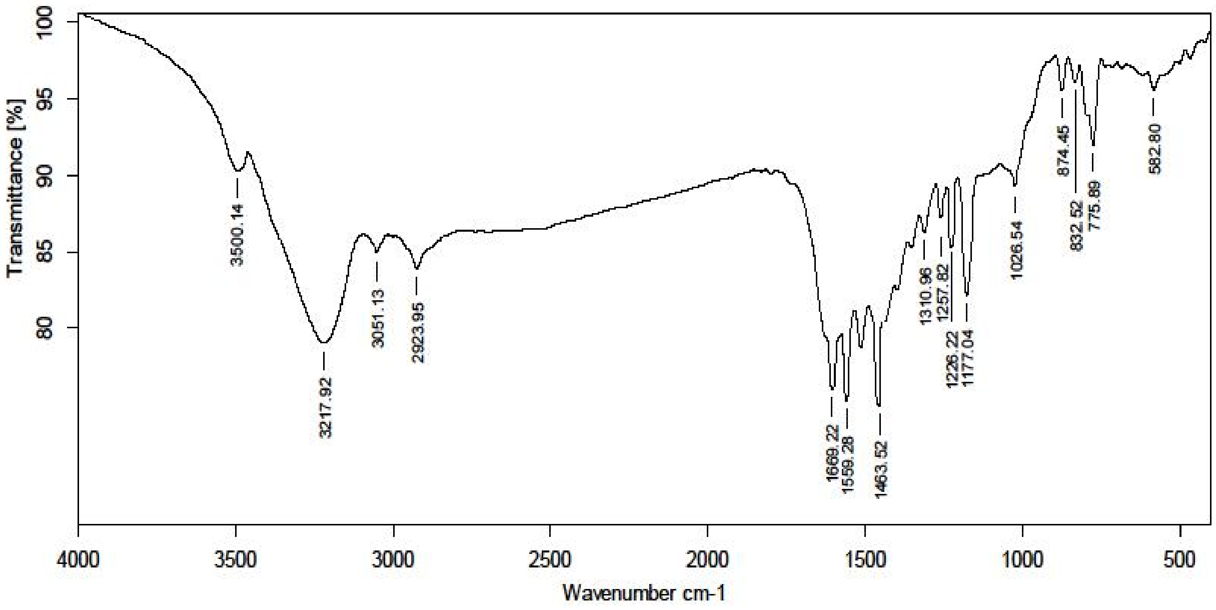

105-TRANSt.0 01:26:22 ã 11/04/2020

Figure S11: IR Spectroscopy of Compound **10a**

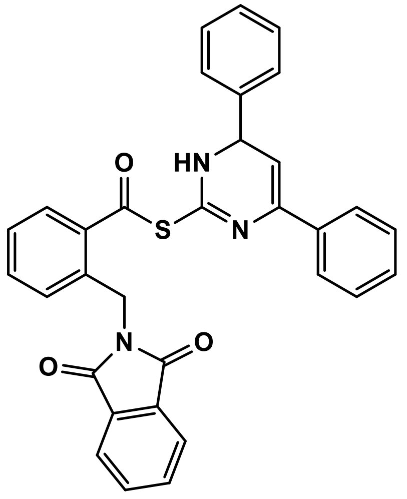

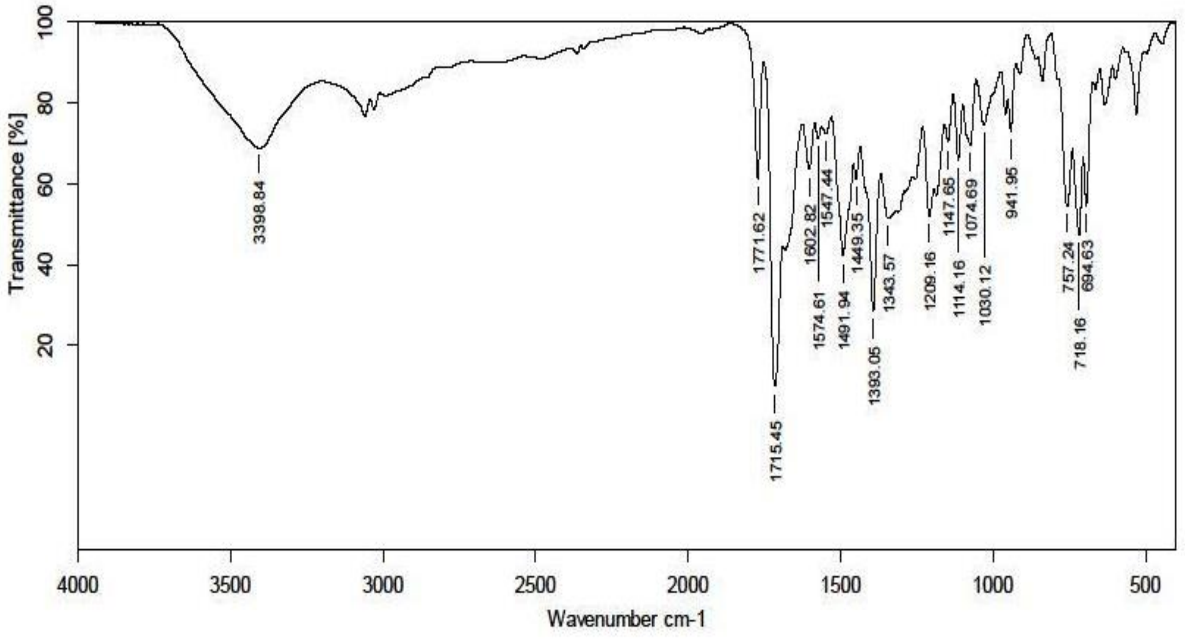

98-TRANS.0 **12:26:37 ä** **13/04/2020**

Figure S12: IR Spectroscopy of Compound **10b**

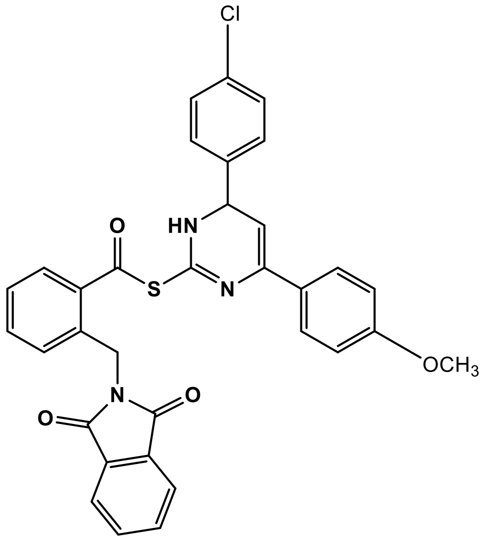

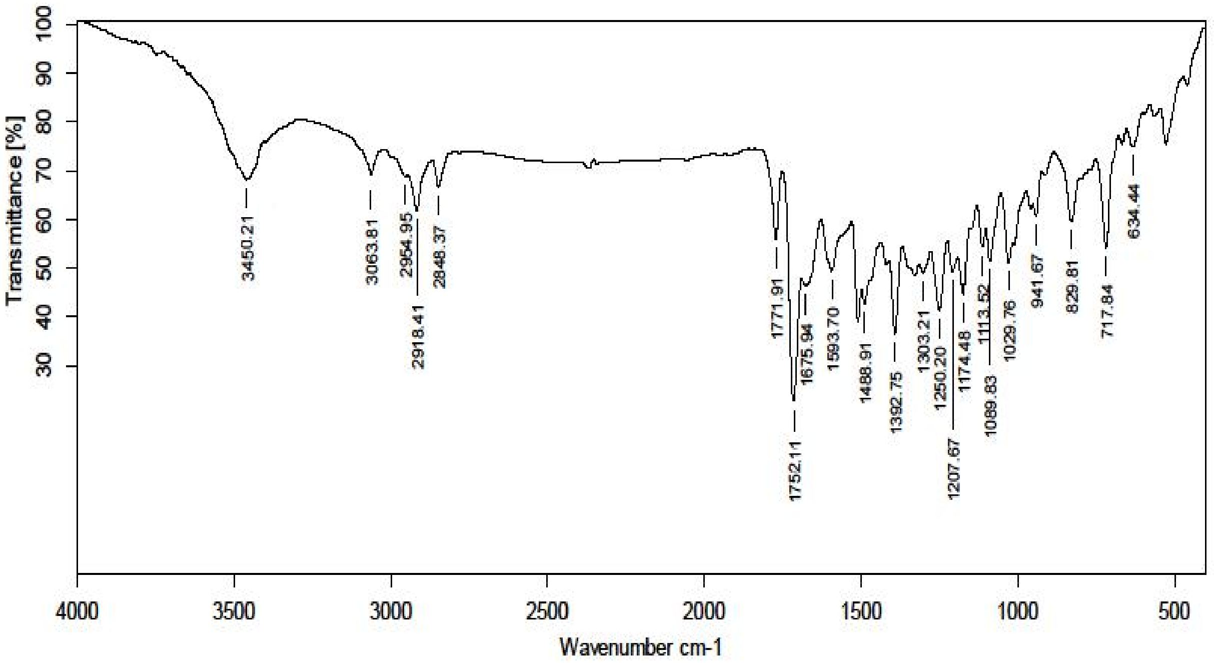

97-TRANSt.0 02:26:11 ä 11/04/2020

Figure S13: IR Spectroscopy of Compound **10c**

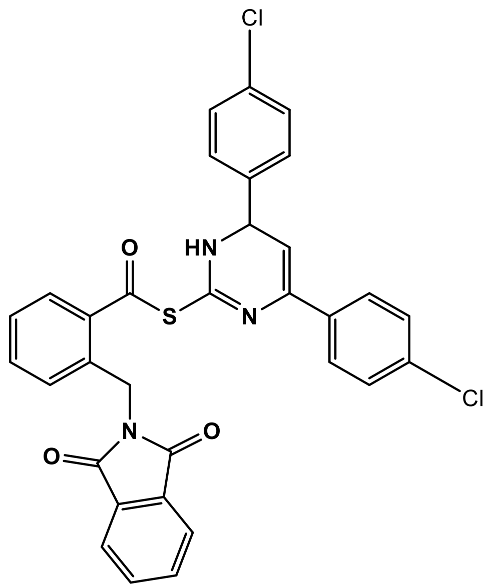

BRUKER

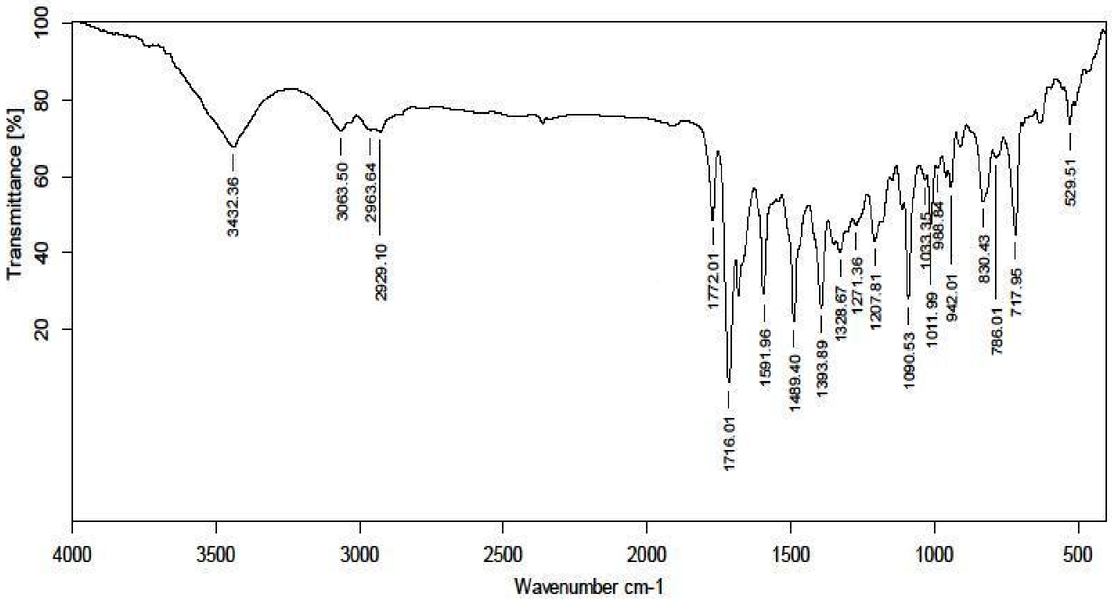

49-TRANSt.0 02:05:51 ä 11/04/2020

Figure S14: IR Spectroscopy of Compound **10d**

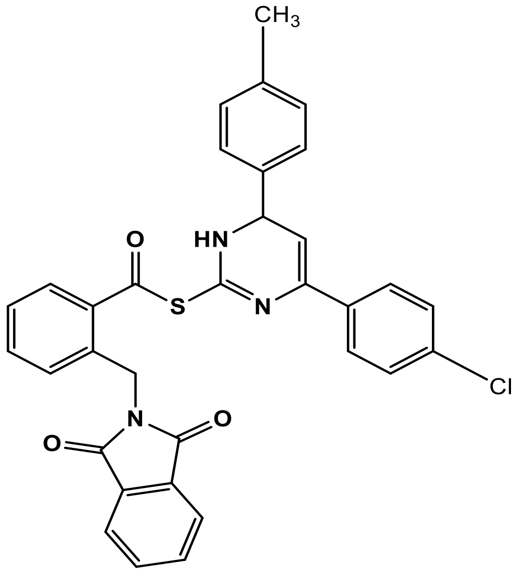

BRUKER

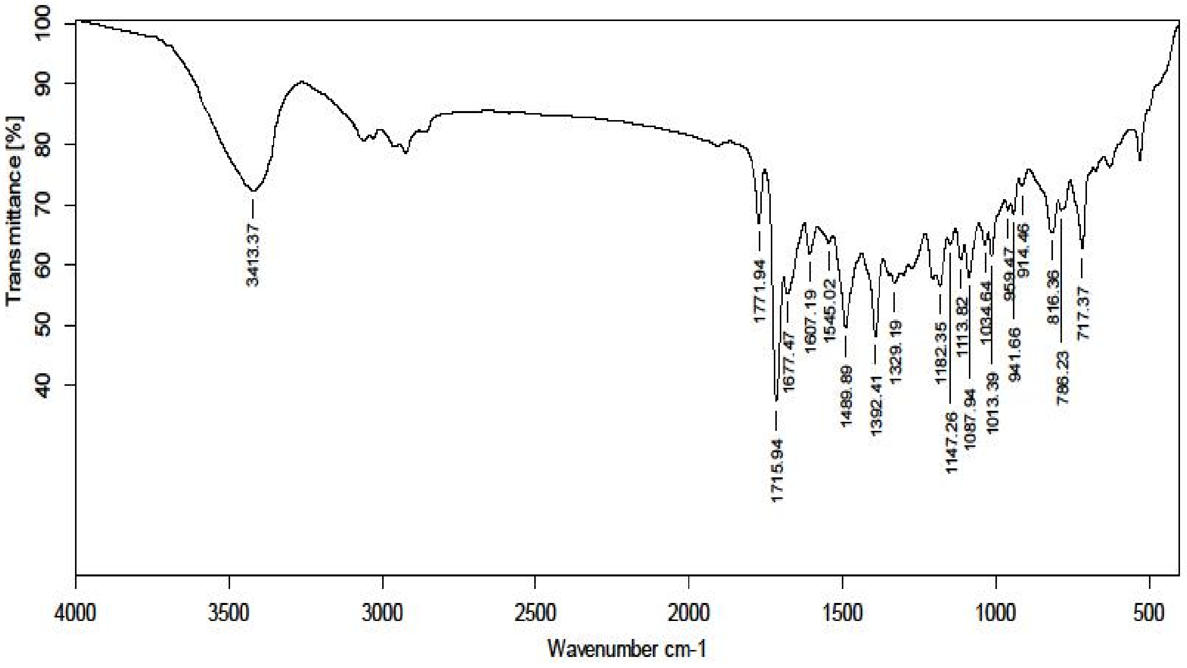

69-TRANS.1 12:27:01 ä 13/04/2020

Figure S15: IR Spectroscopy of Compound **10e**

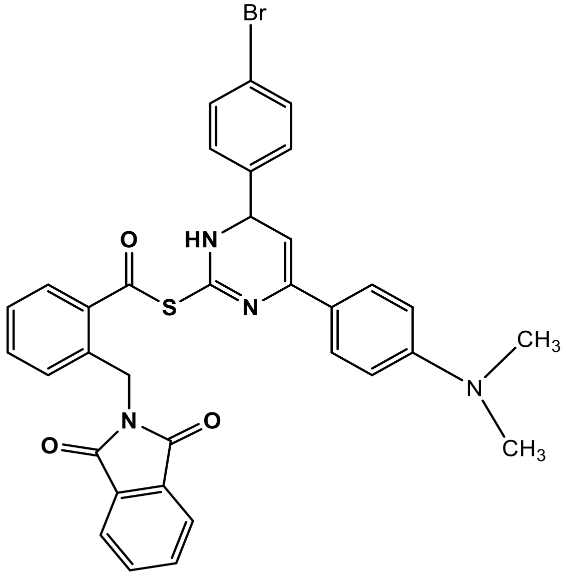

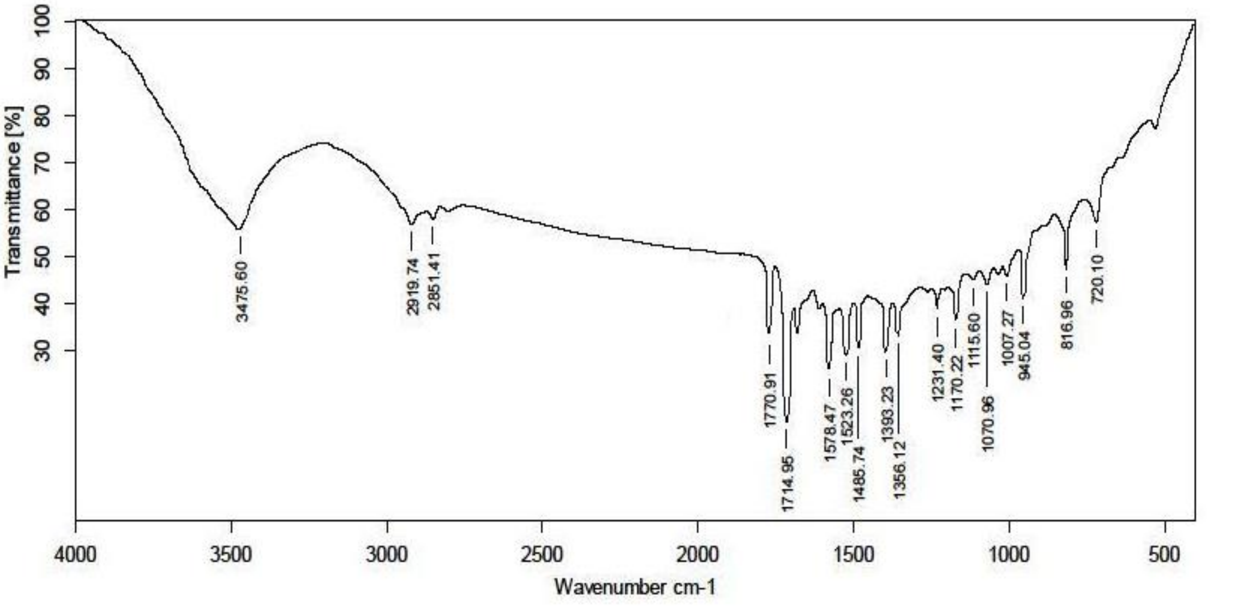

89-TRANSt.0 02:12:27 ã 11/04/2020

Figure S16: IR Spectroscopy of Compound **10f**

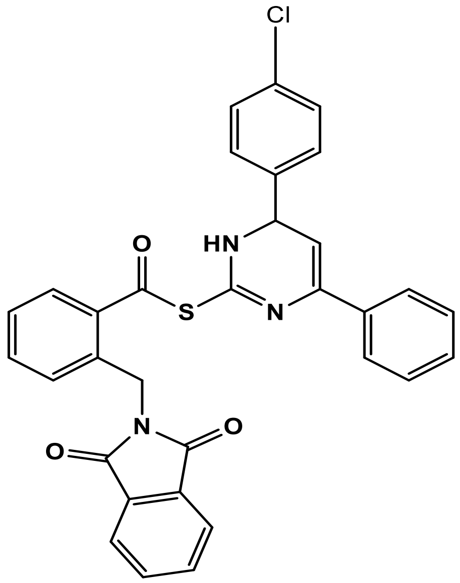

BRUKER

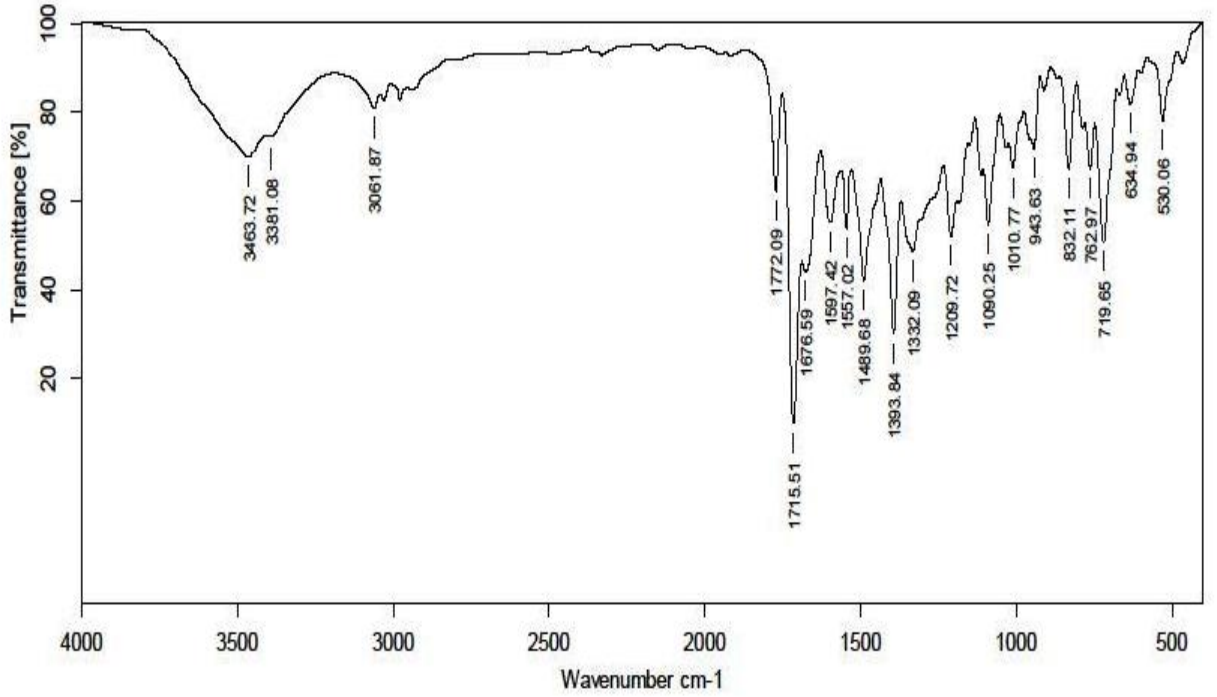

101-TRANSt.0 02:17:19 ä 11/04/2020

Figure S17: IR Spectroscopy of Compound **10g**

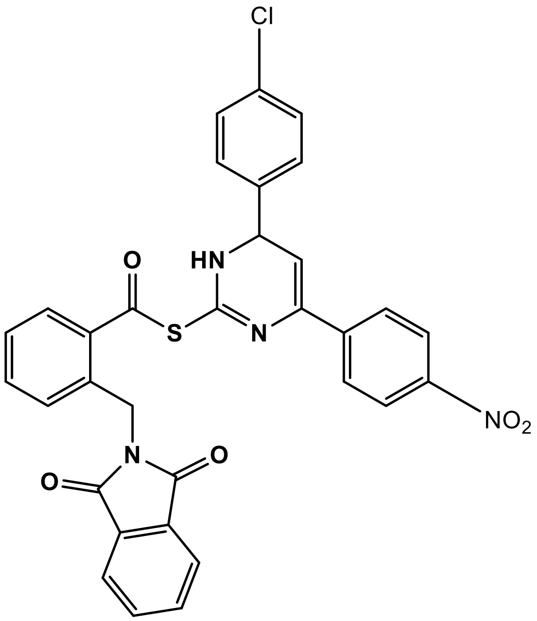

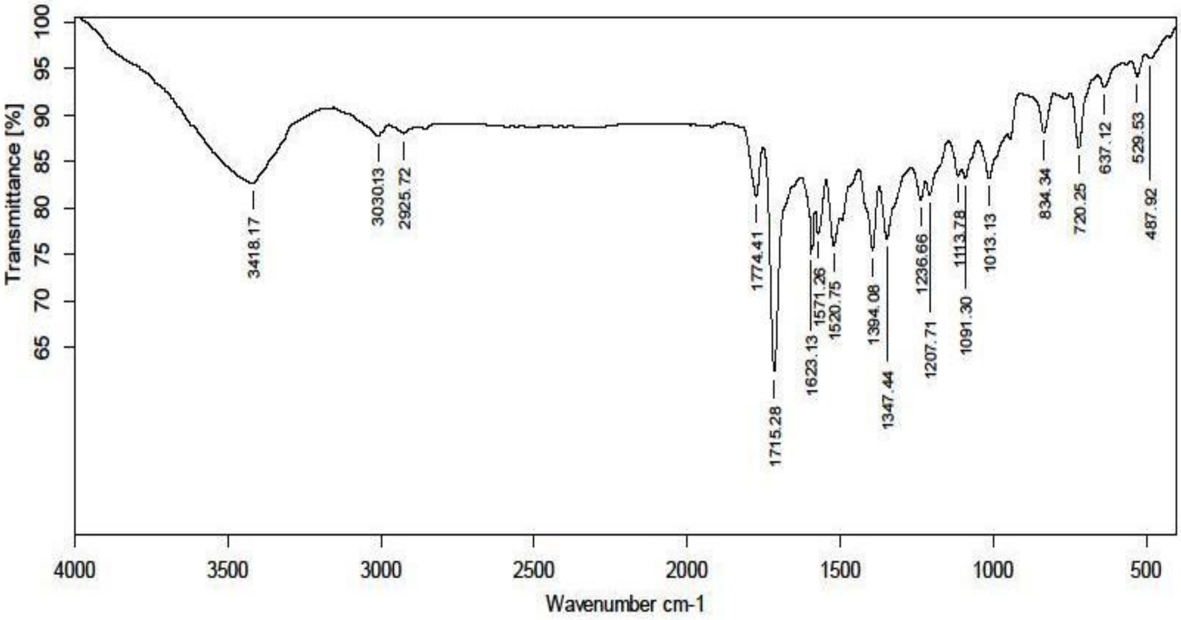

102-TRANSt.0 02:21:32 ä 11/04/2020

Figure S18: IR Spectroscopy of Compound **10h**

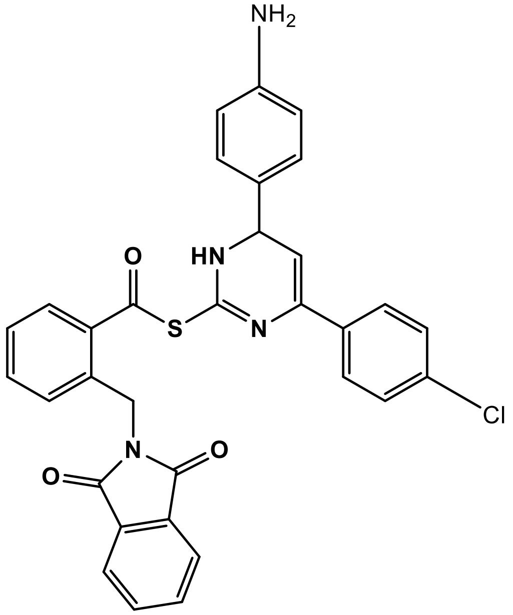

BRUKER

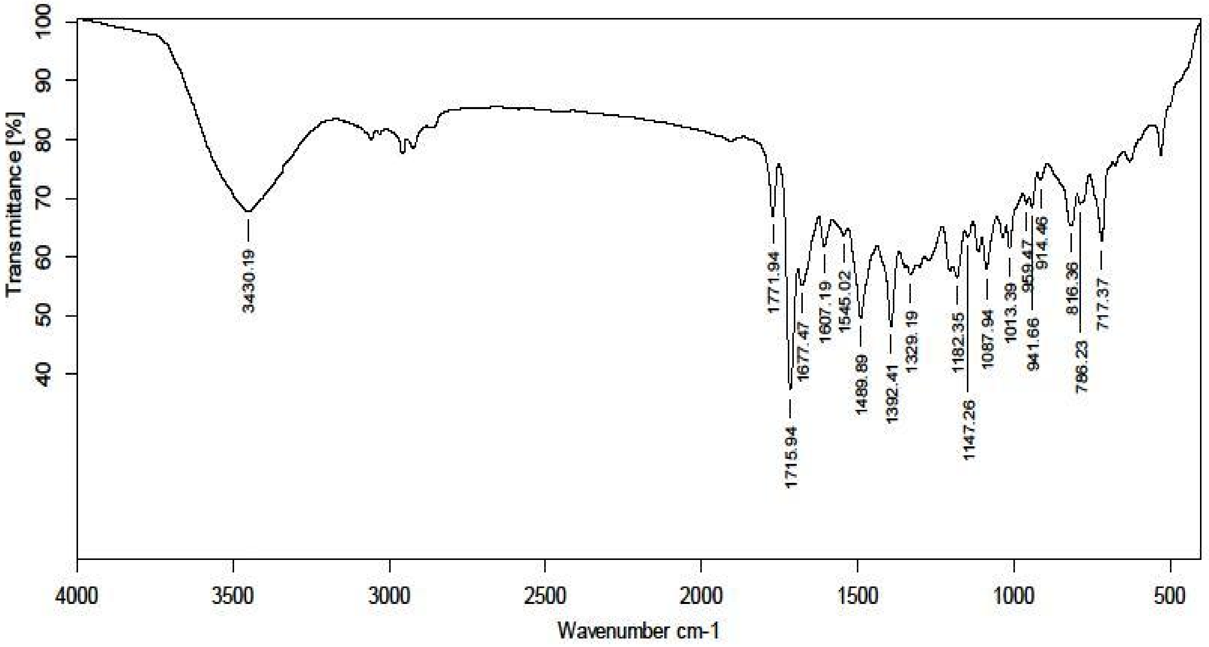

69-TRANS.1 12:27:01 ã 13/04/2020

Figure S19: IR Spectroscopy of Compound **10i**

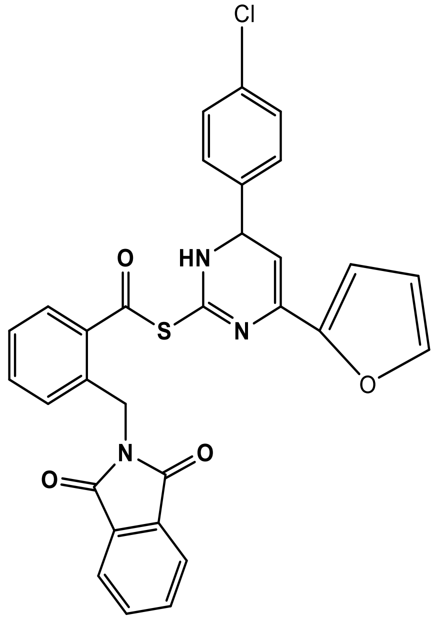

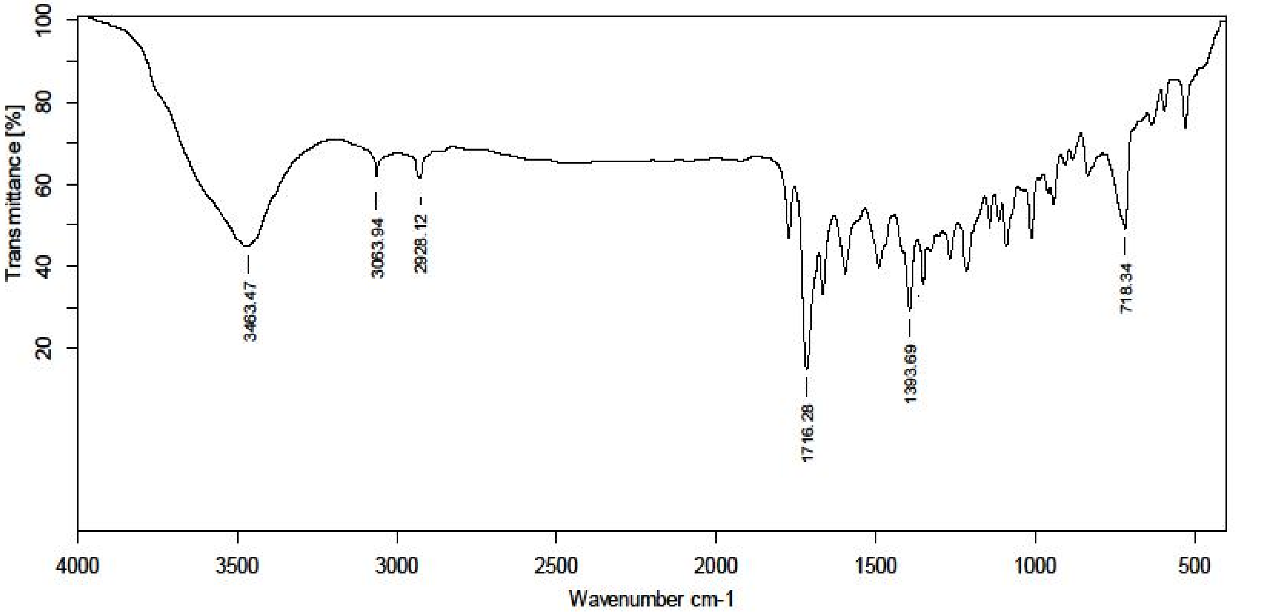

100-TRANS.0 12:25:36 ä 13/04/2020

Figure S20: IR Spectroscopy of Compound **10j**

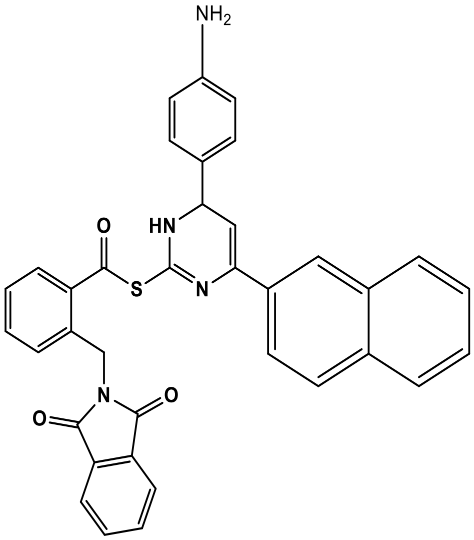

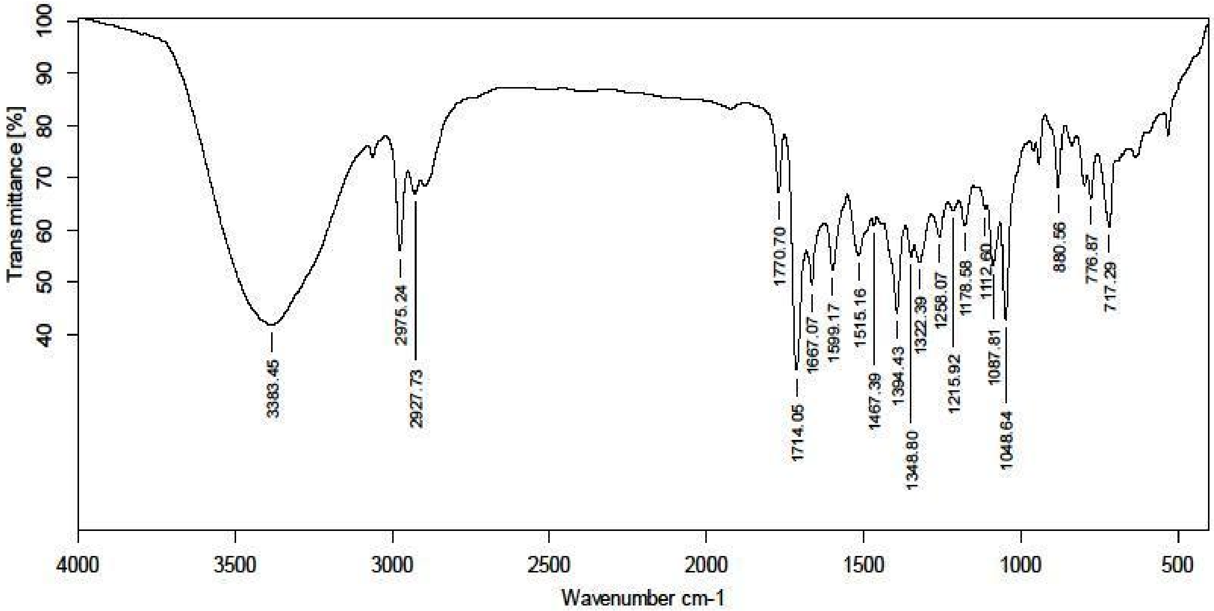

106-TRANS.0 12:12:06 ä 13/04/2020

^{1}H NMR Spectroscopy

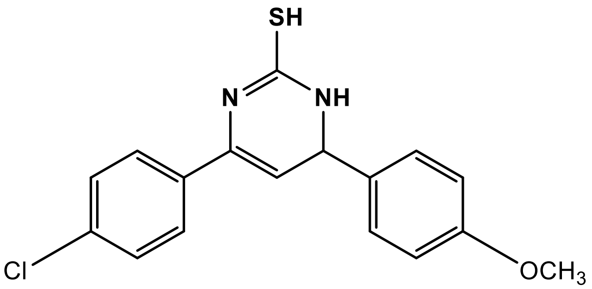

Figure S21: ^{1}H NMR Spectroscopy of Compound **5b**

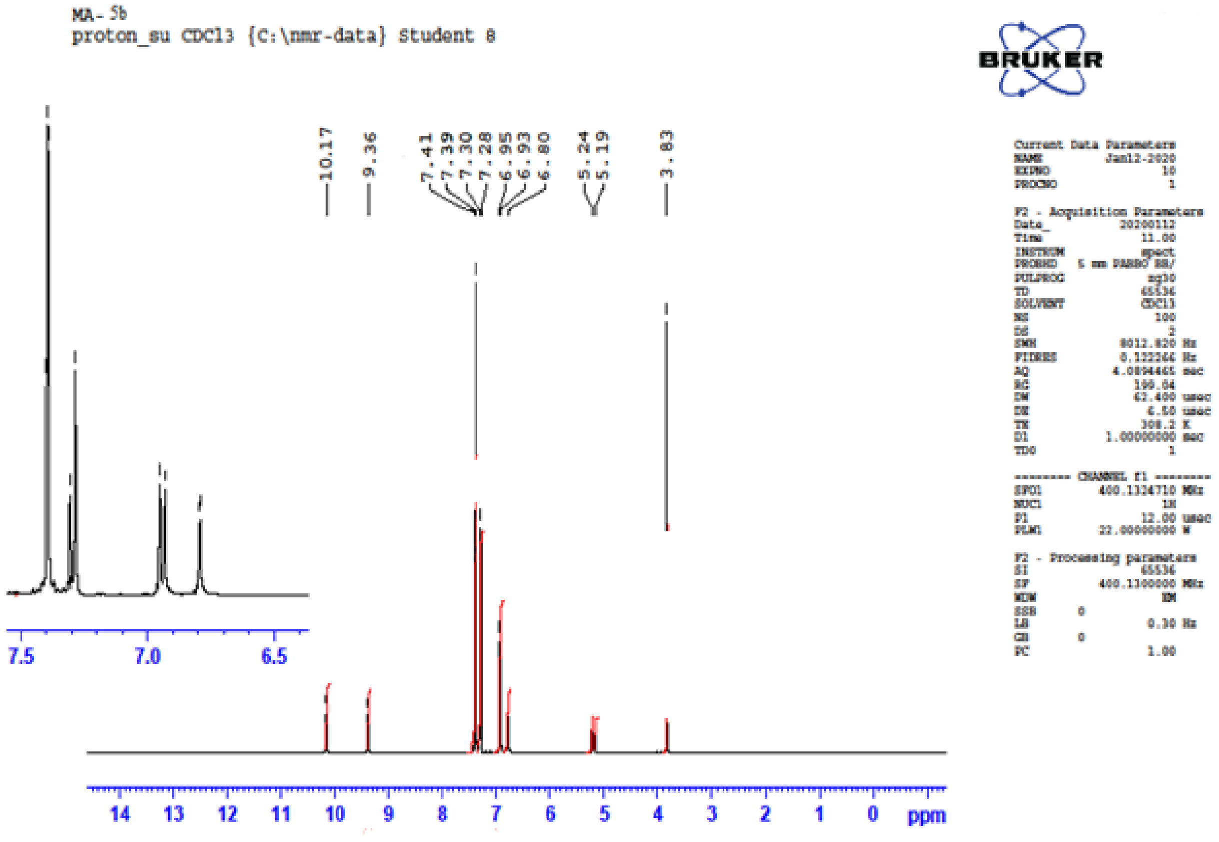

Figure S22: ^{1}H NMR Spectroscopy of Compound **5c**

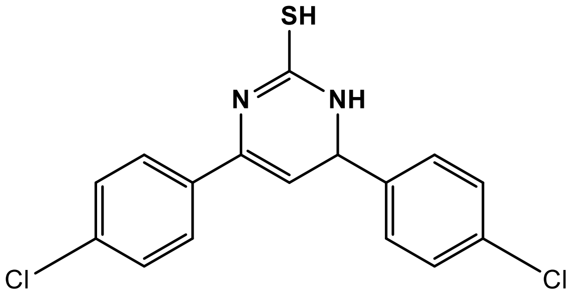

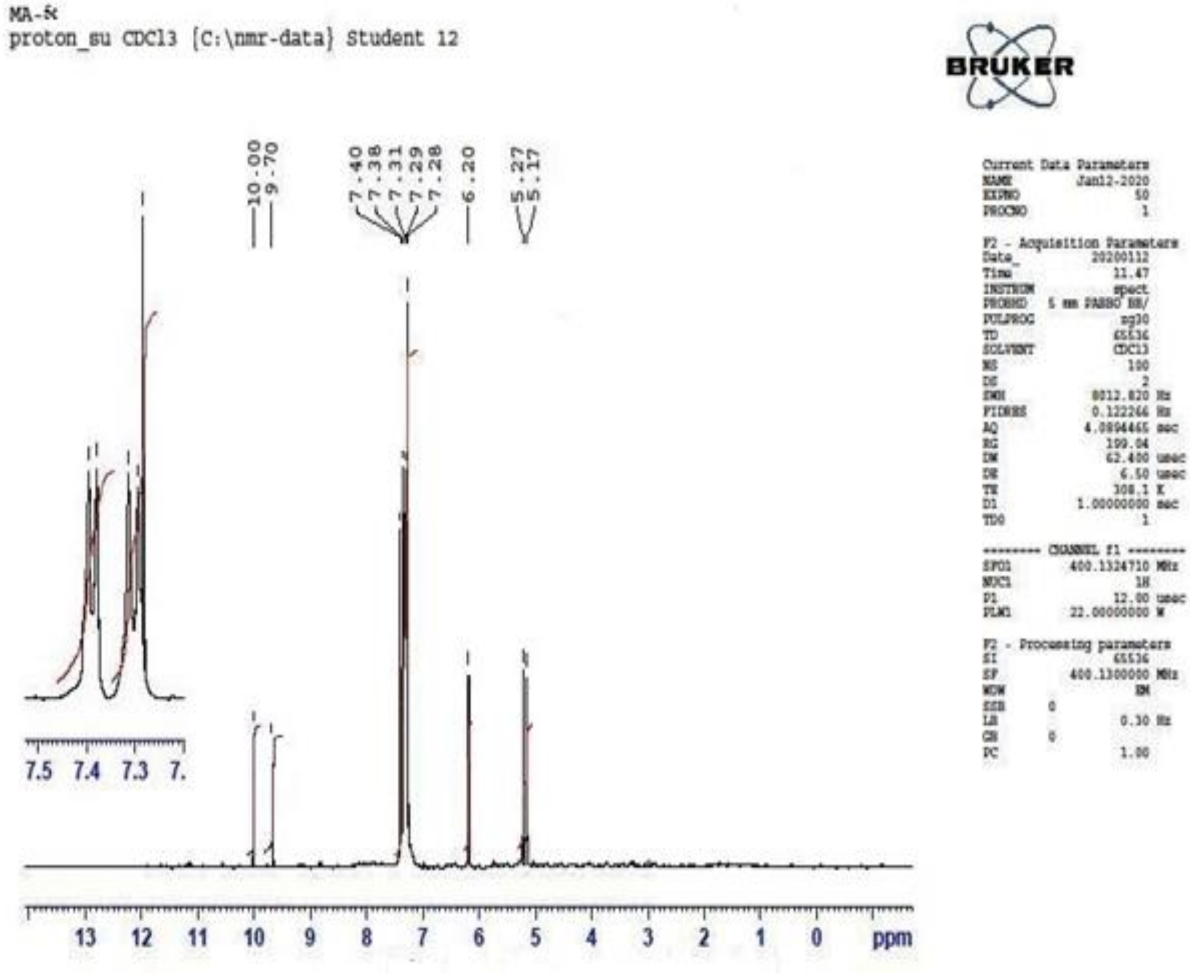

Figure S23: ^{1}H NMR Spectroscopy of Compound **5d**

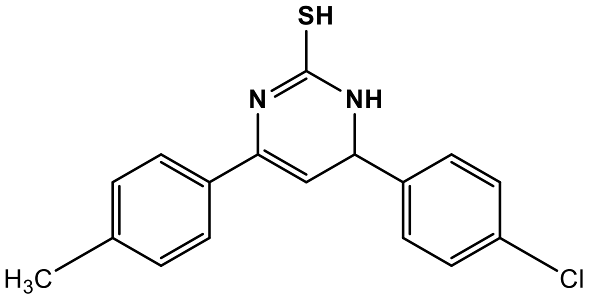

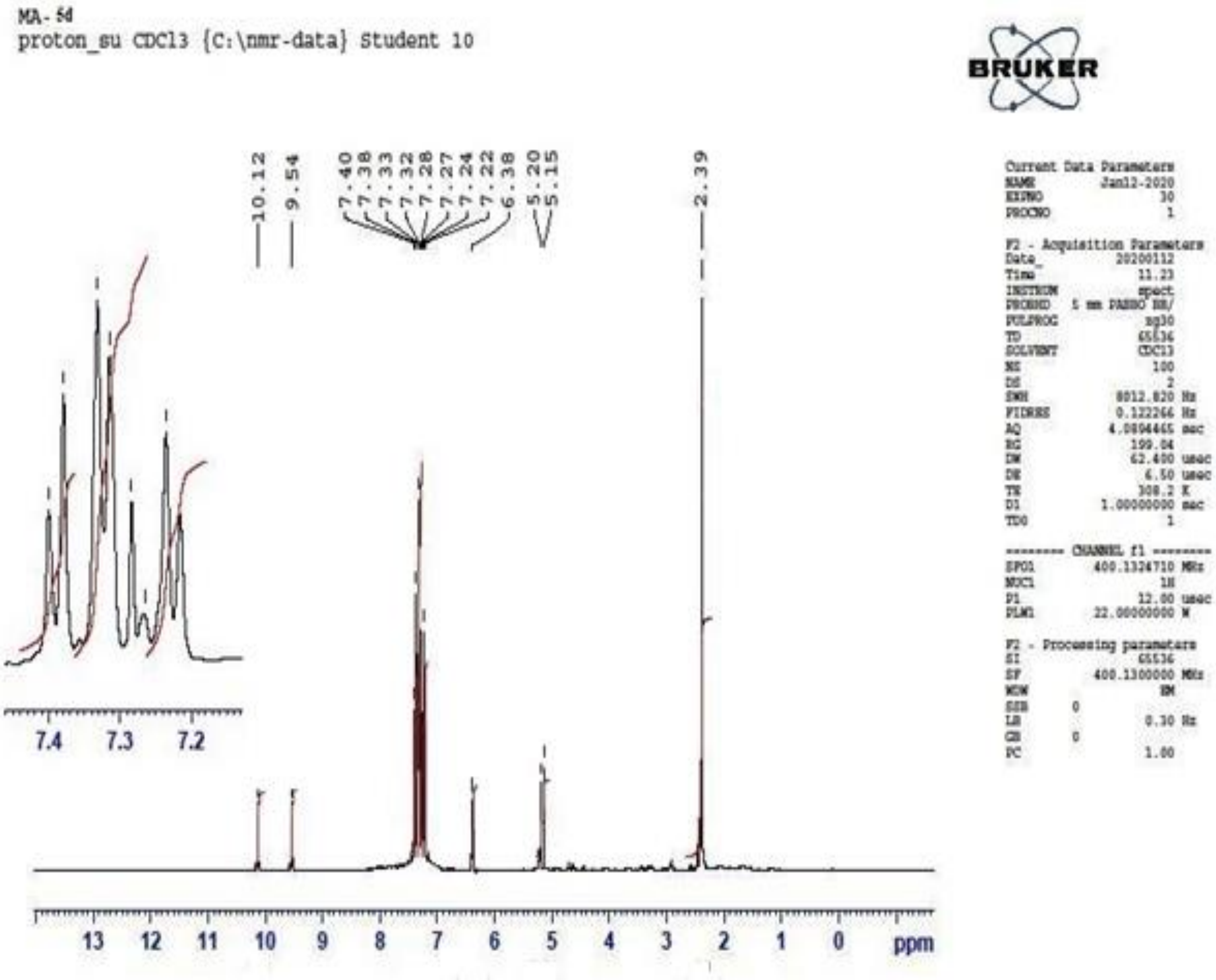

Figure S24: ^{1}H NMR Spectroscopy of Compound **5e**

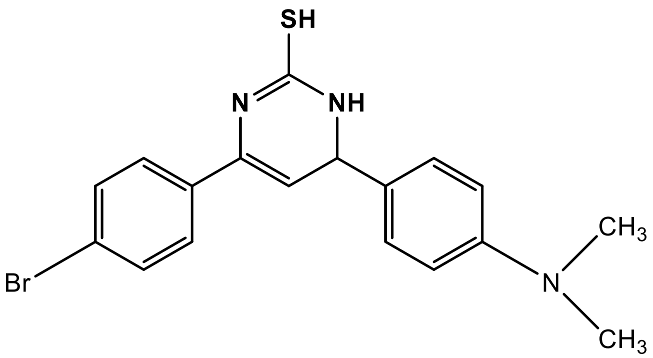

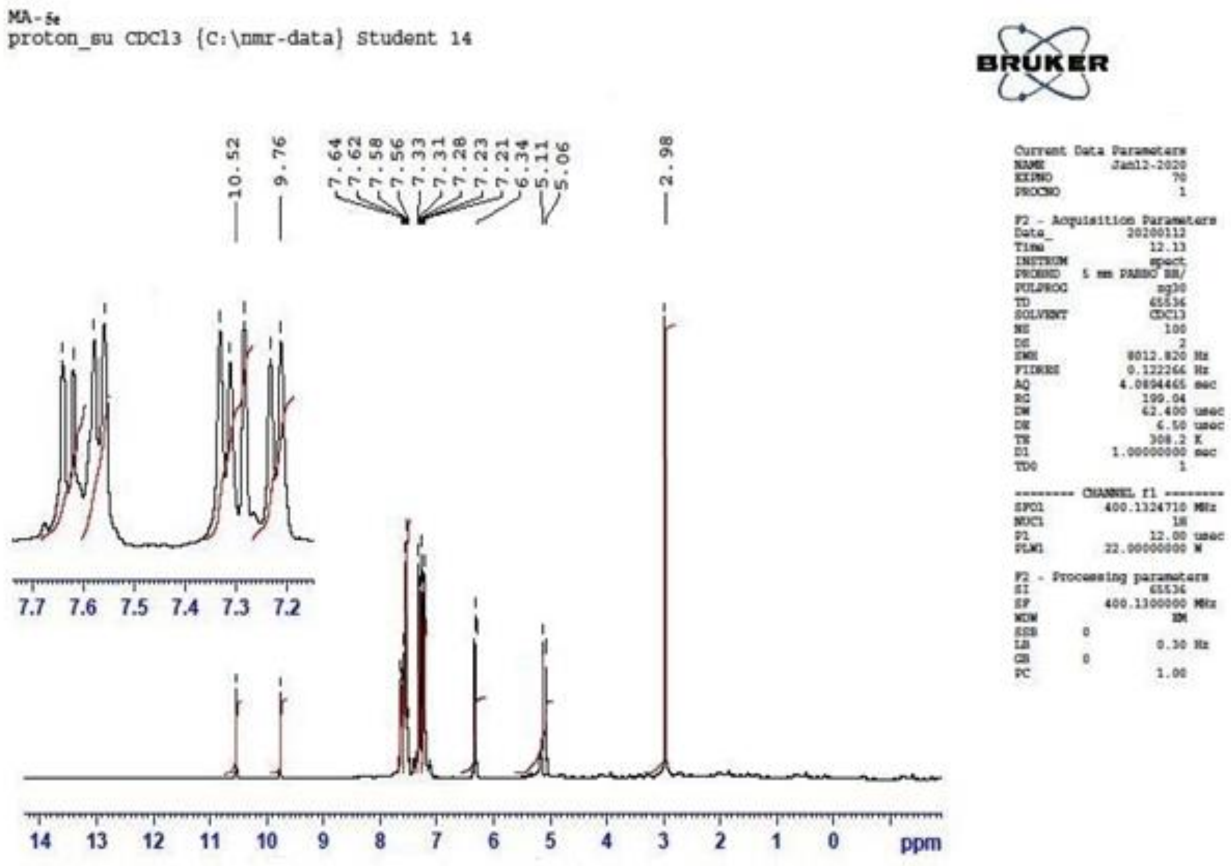

Figure S25: ^{1}H NMR Spectroscopy of Compound **5f**

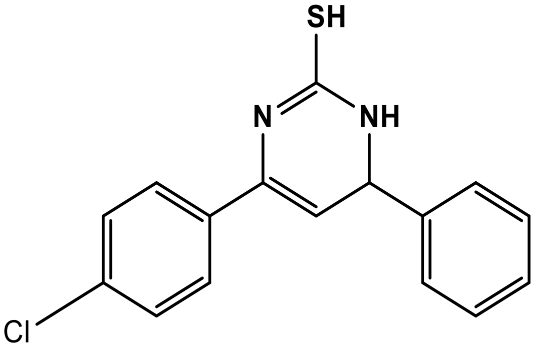

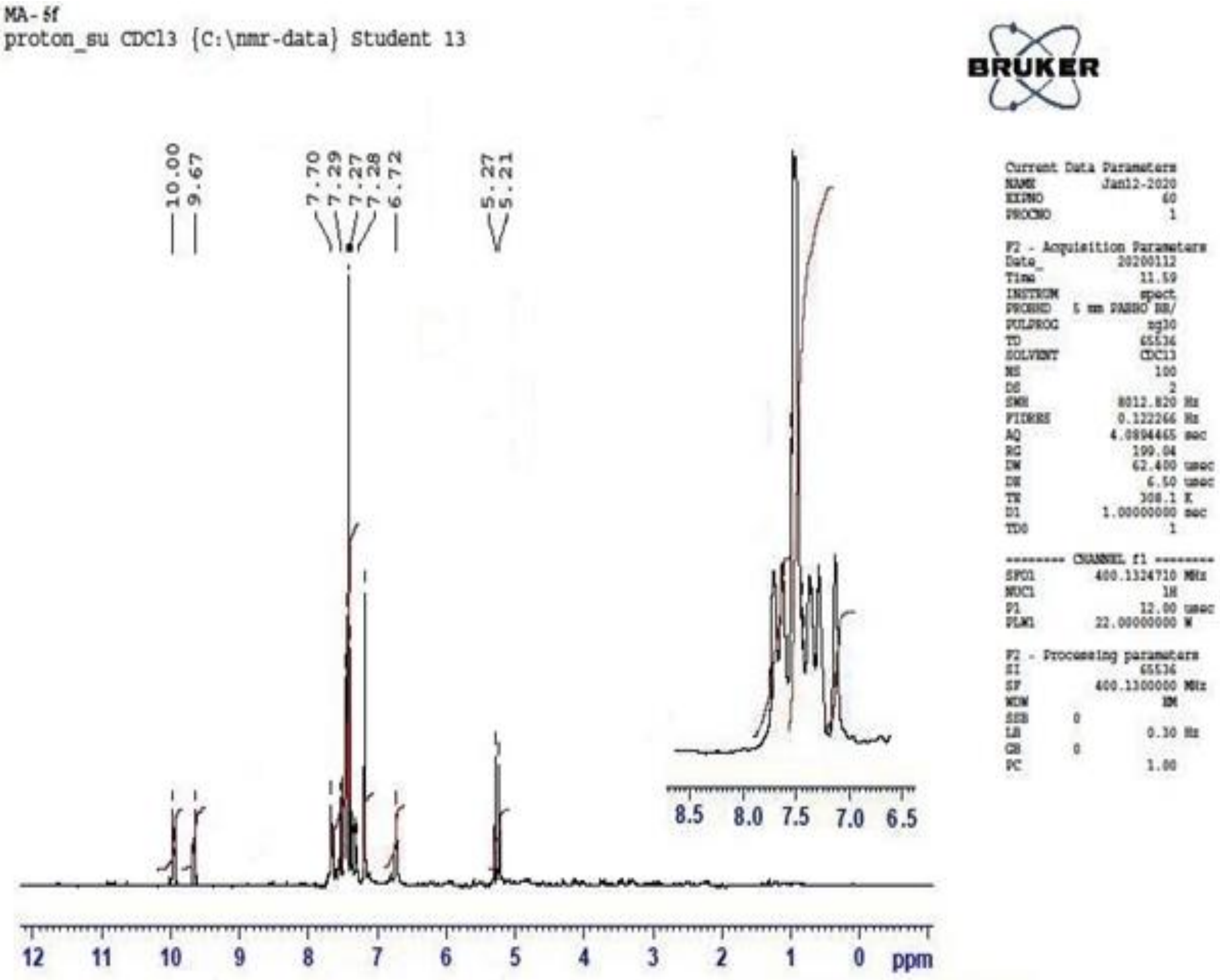

Figure S26: ^{1}H NMR Spectroscopy of Compound **5g**

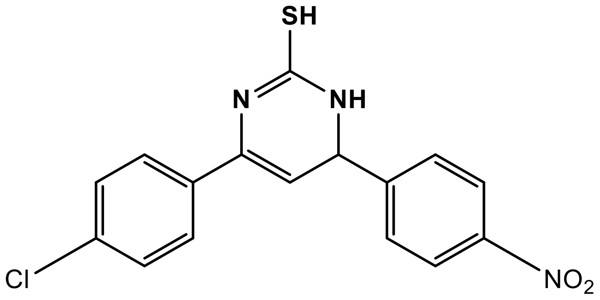

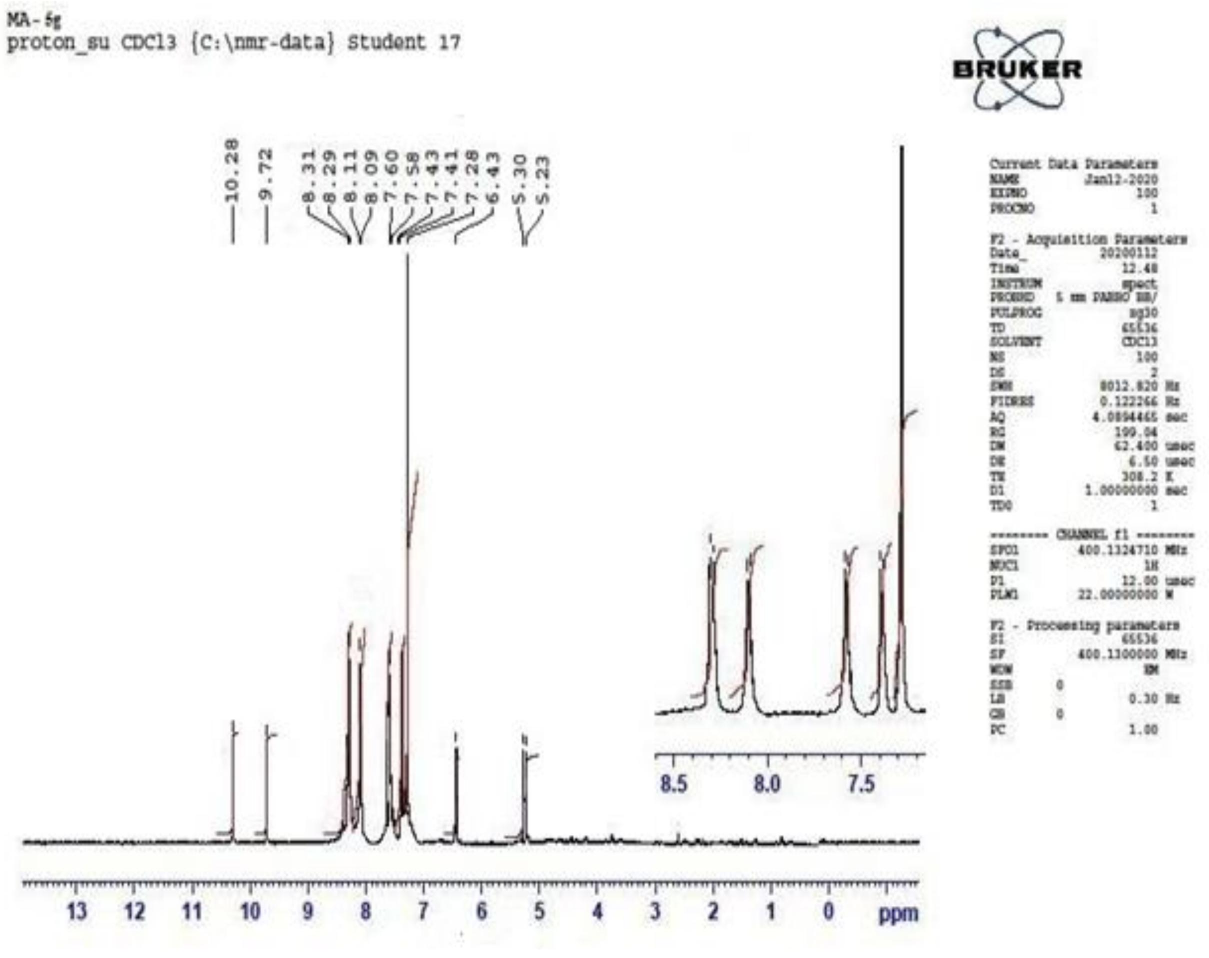

Figure S27: ^{1}H NMR Spectroscopy of Compound **5h**

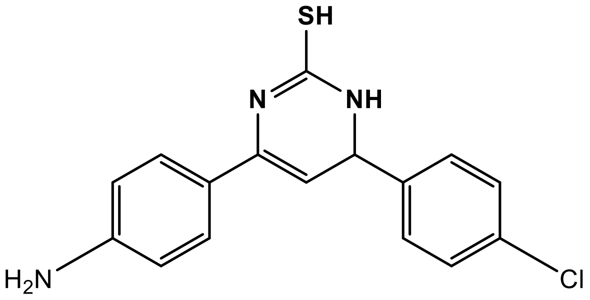

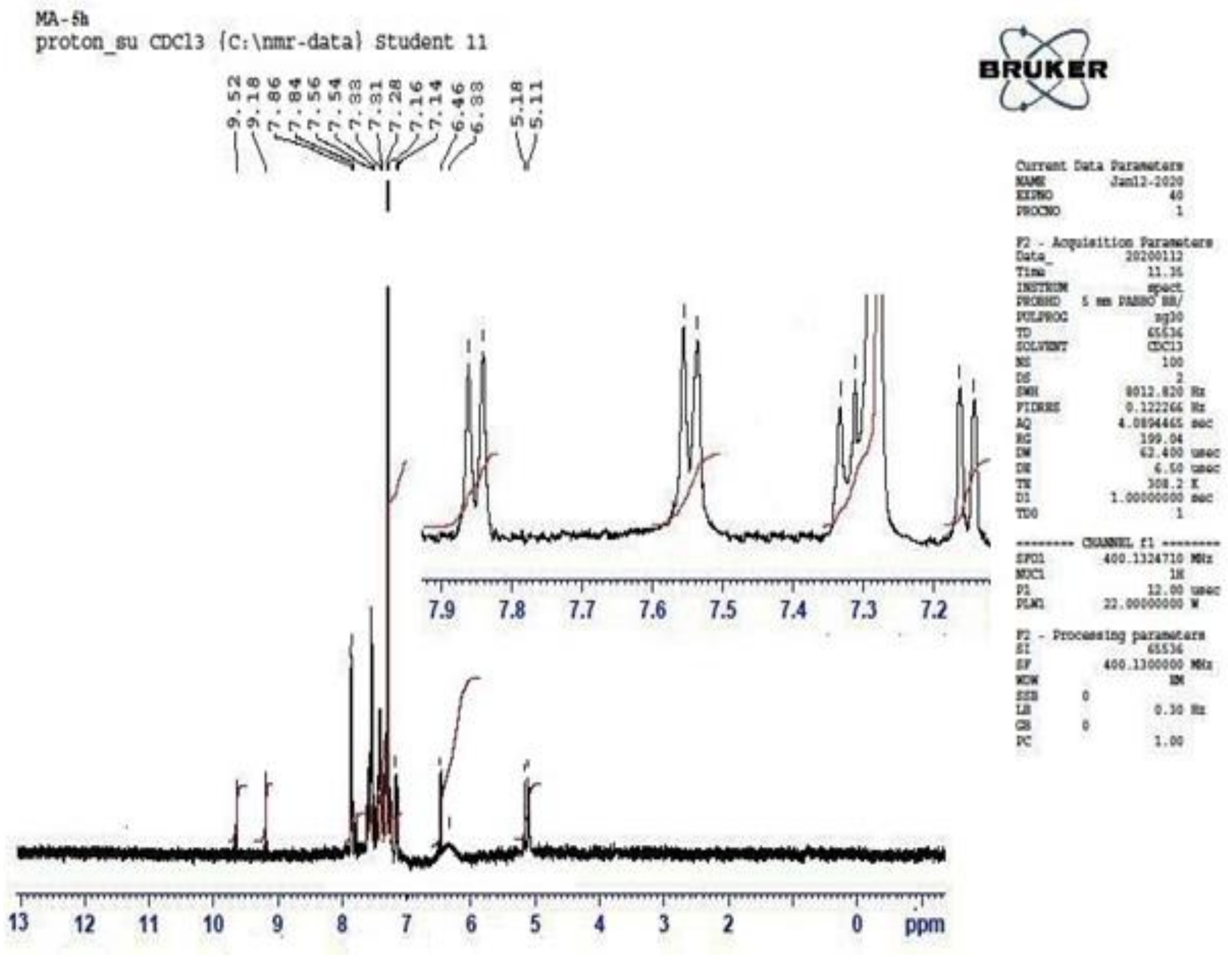

Figure S28: ^{1}H NMR Spectroscopy of Compound **5i**

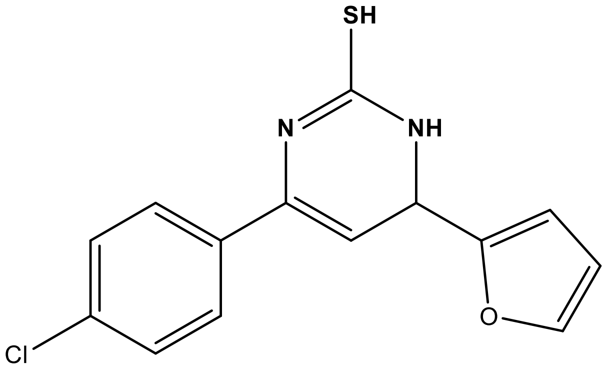

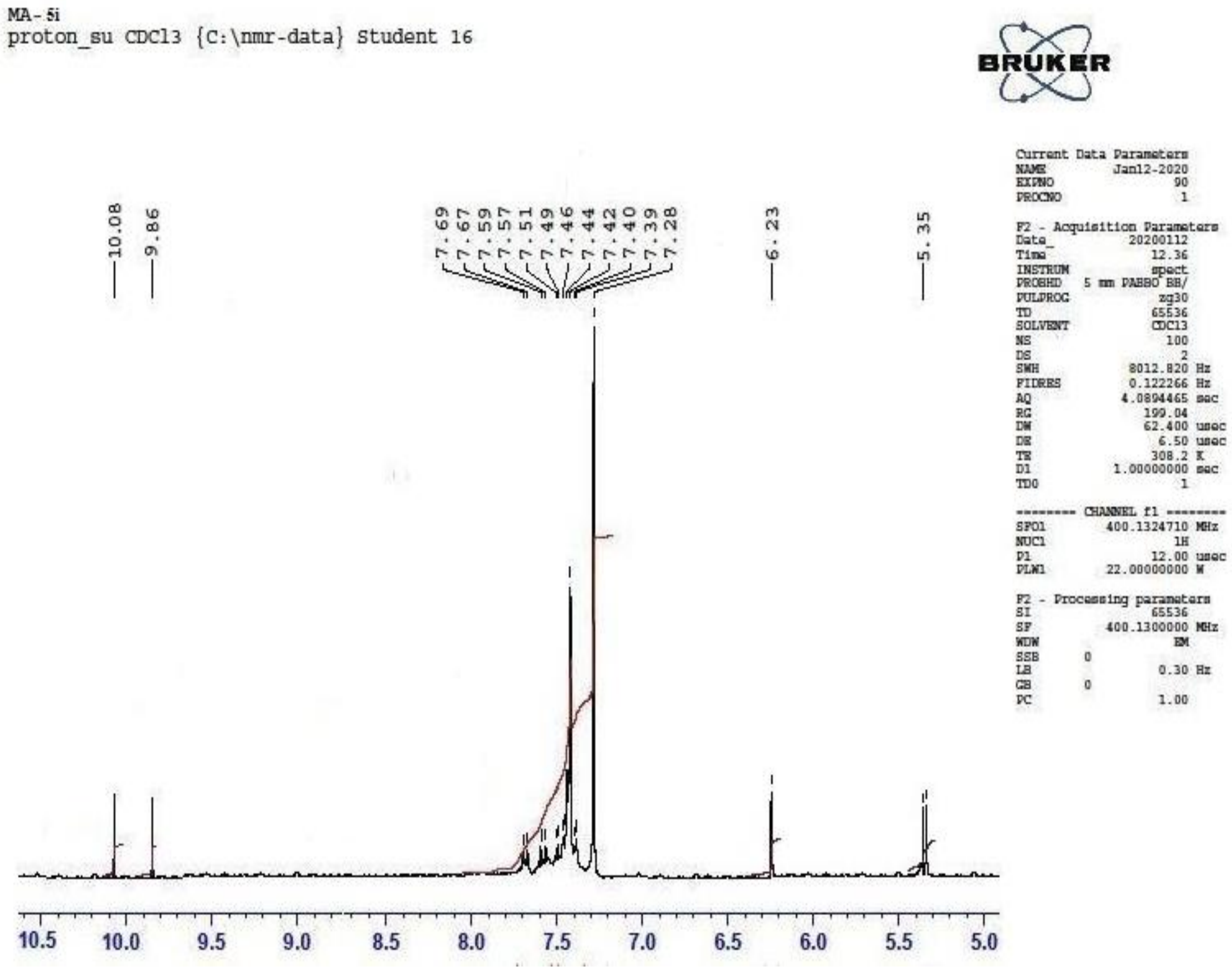

Figure S29: ^{1}H NMR Spectroscopy of Compound **5j**

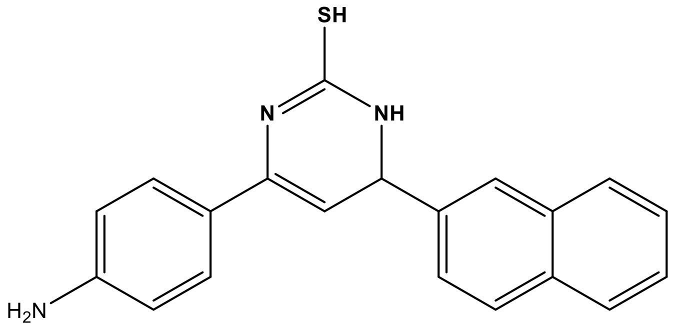

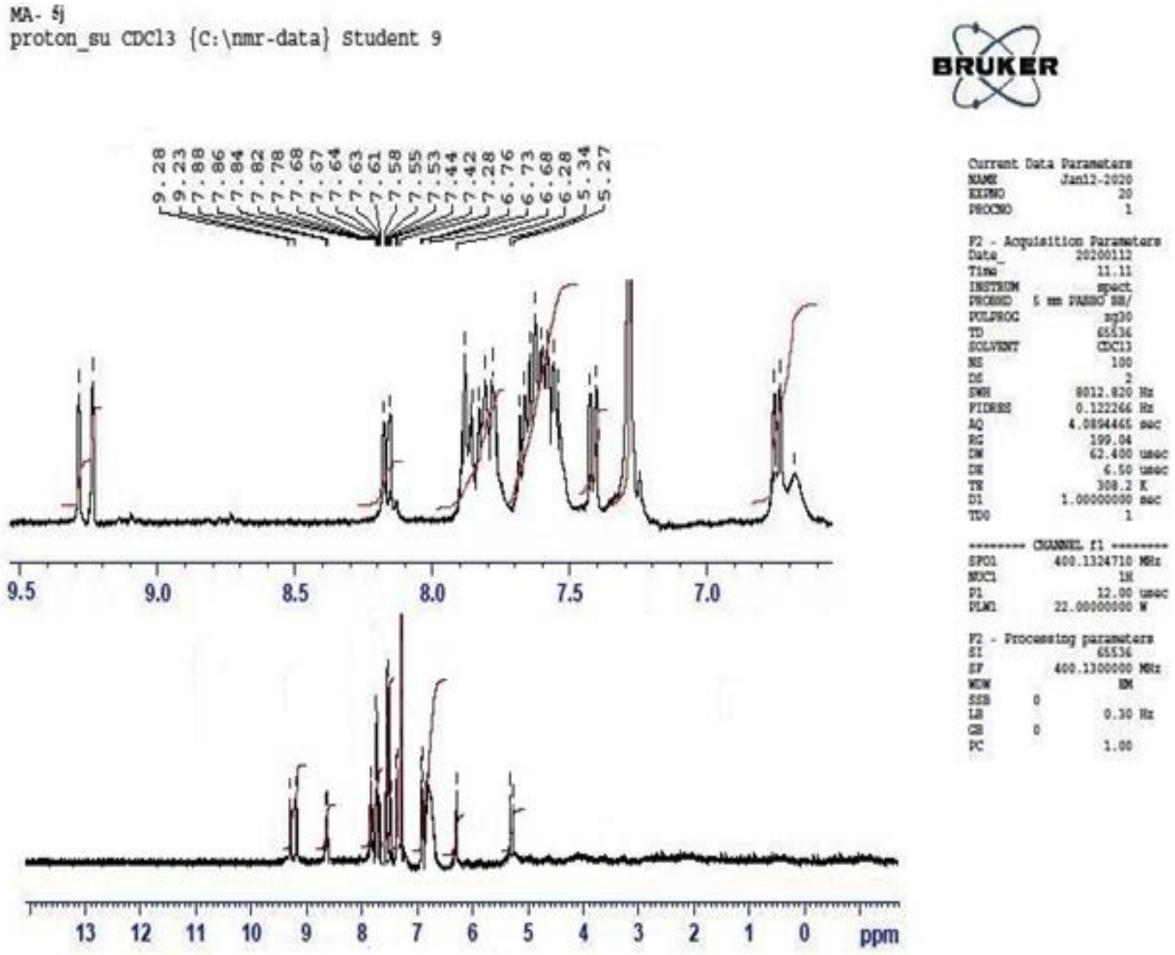

Figure S30: ^{1}H NMR Spectroscopy of Compound **10a**

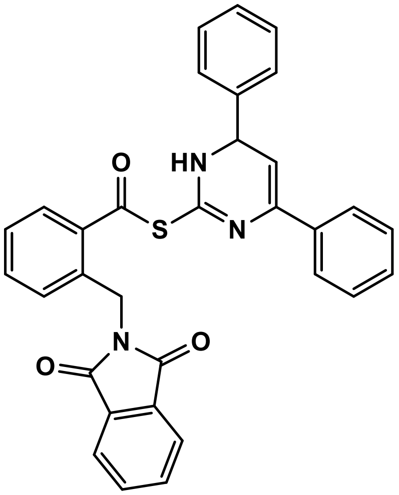

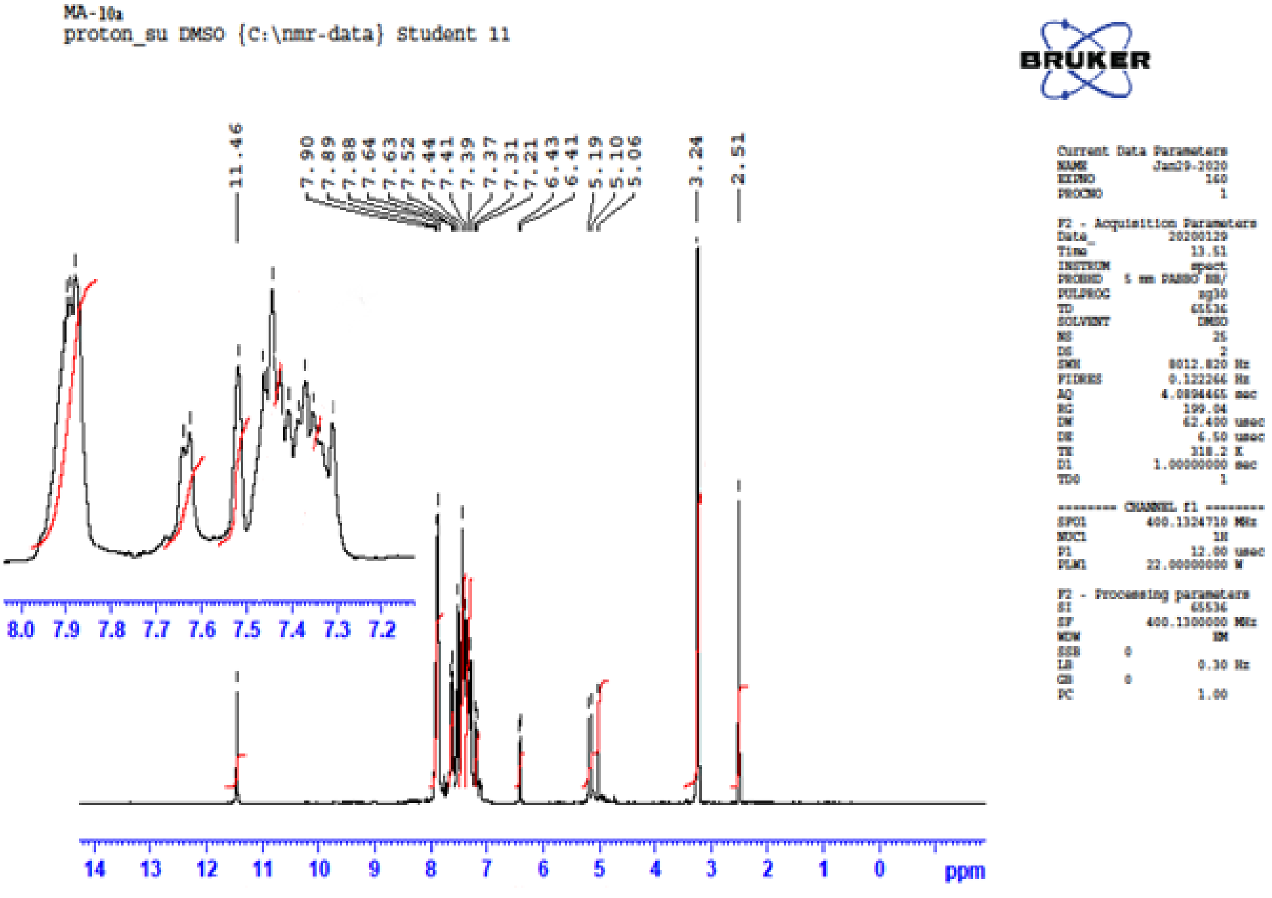

Figure S31: ^{1}H NMR Spectroscopy of Compound **10b**

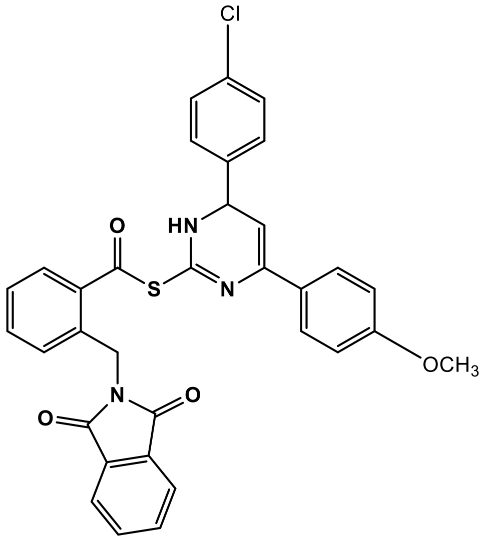

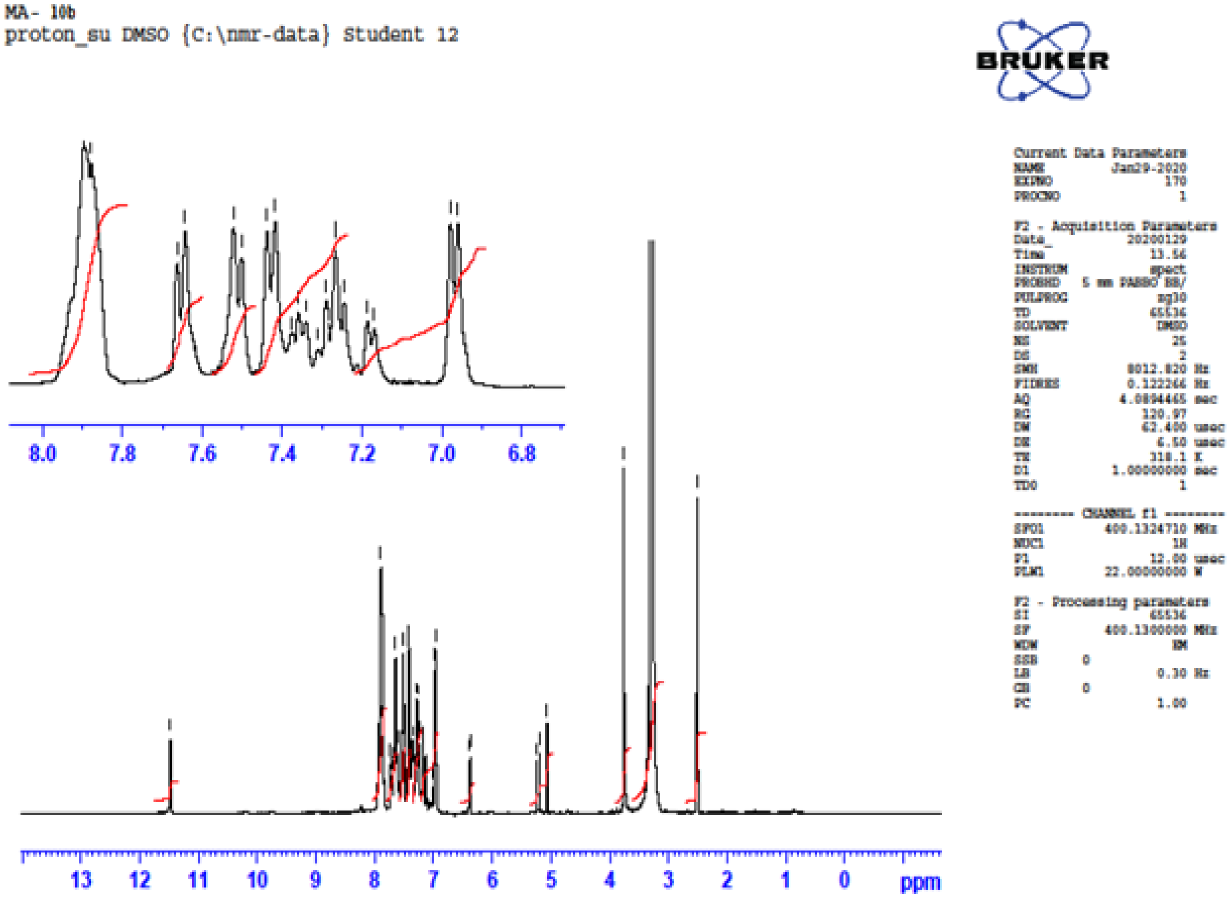

Figure S32: ^{1}H NMR Spectroscopy of Compound **10c**

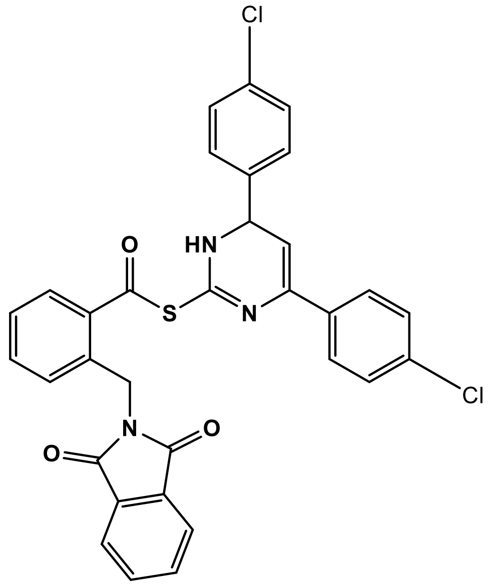

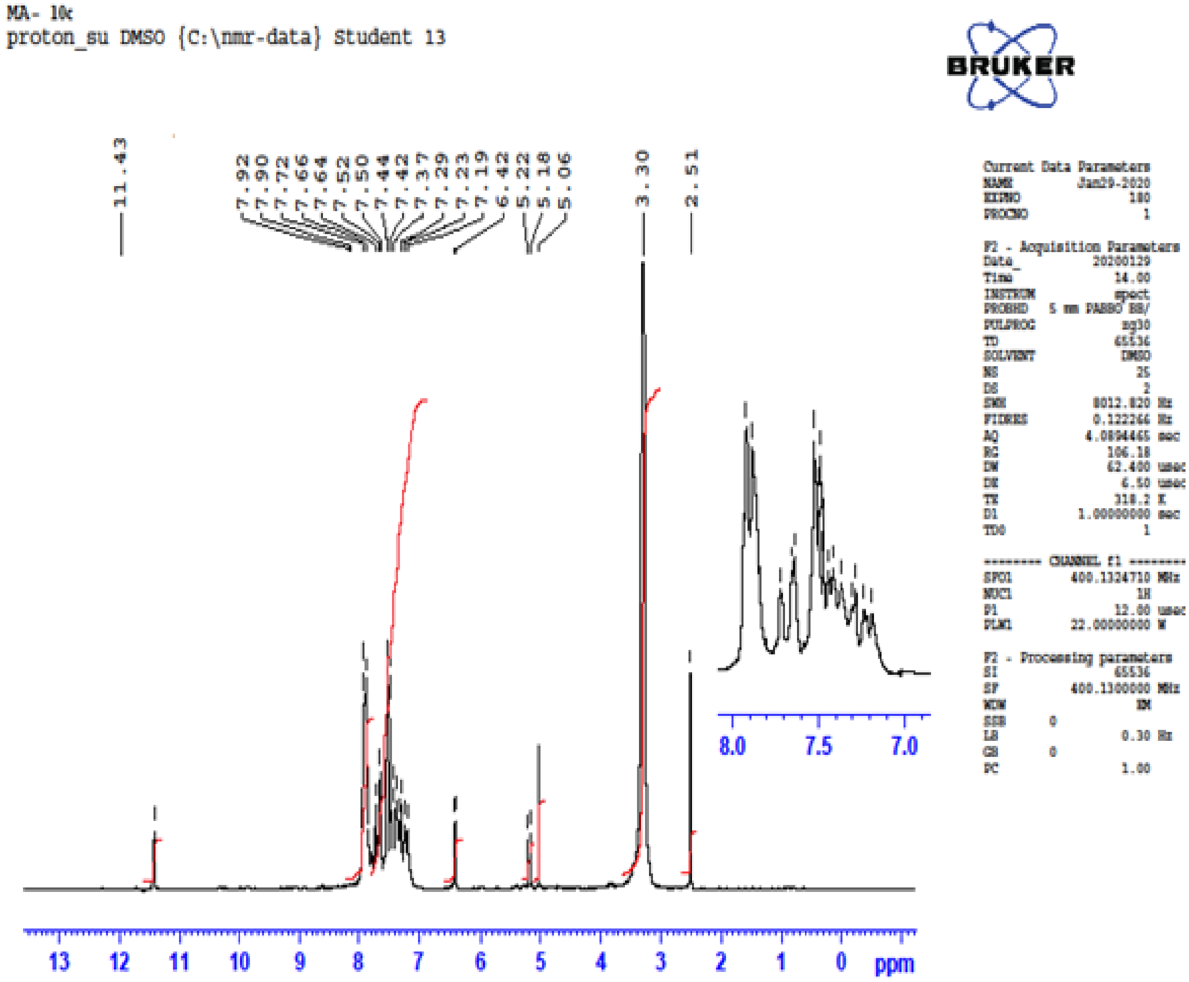

Figure S33: ^{1}H NMR Spectroscopy of Compound **10d**

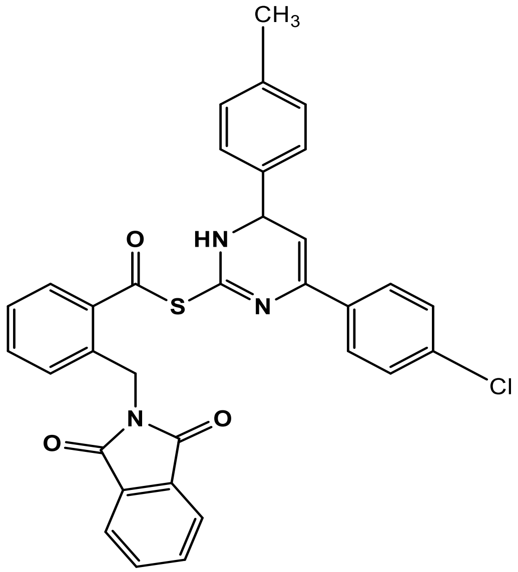

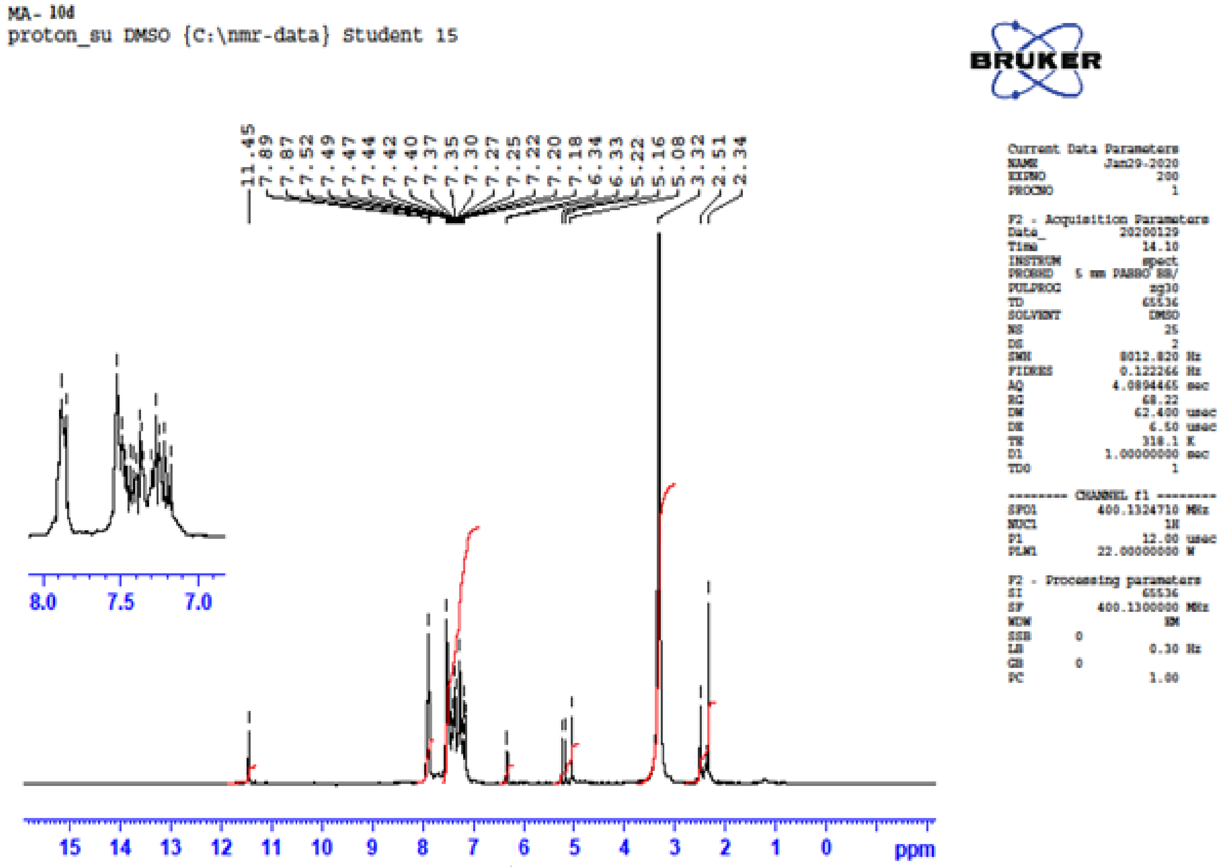

Figure S34: ^{1}H NMR Spectroscopy of Compound **10e**

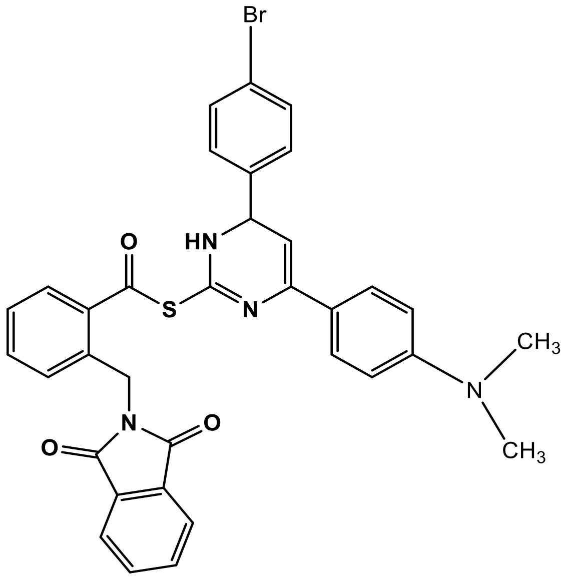

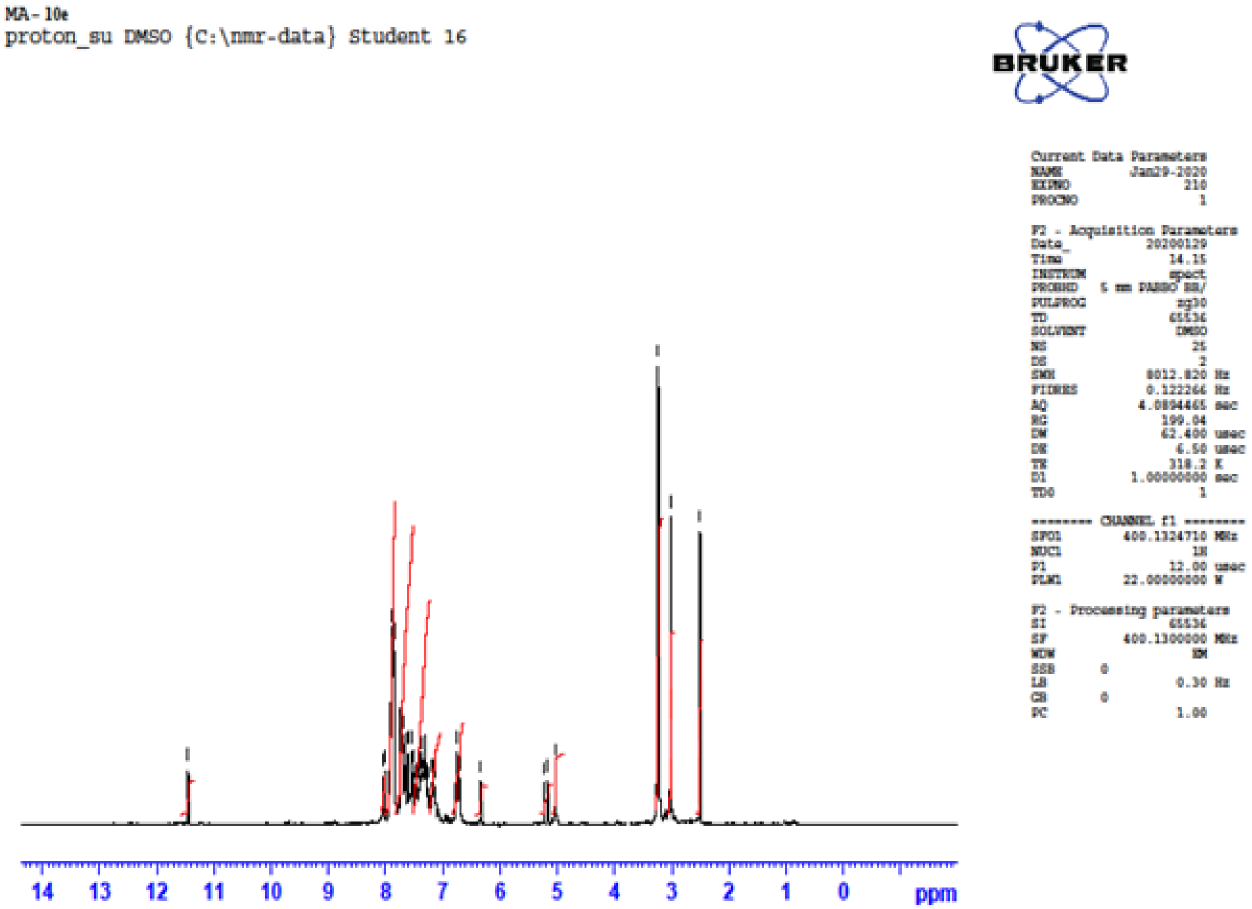

Figure S35: ^{1}H NMR Spectroscopy of Compound **10f**

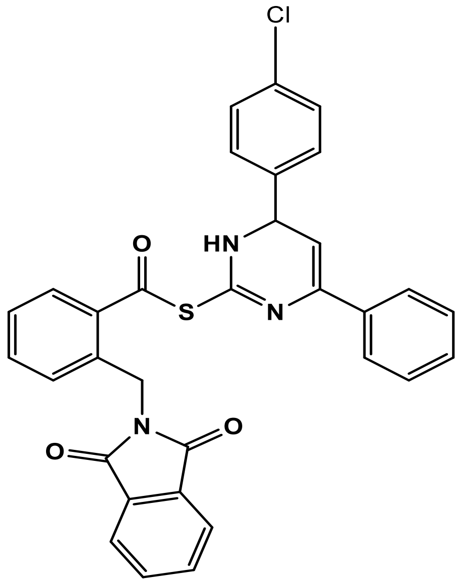

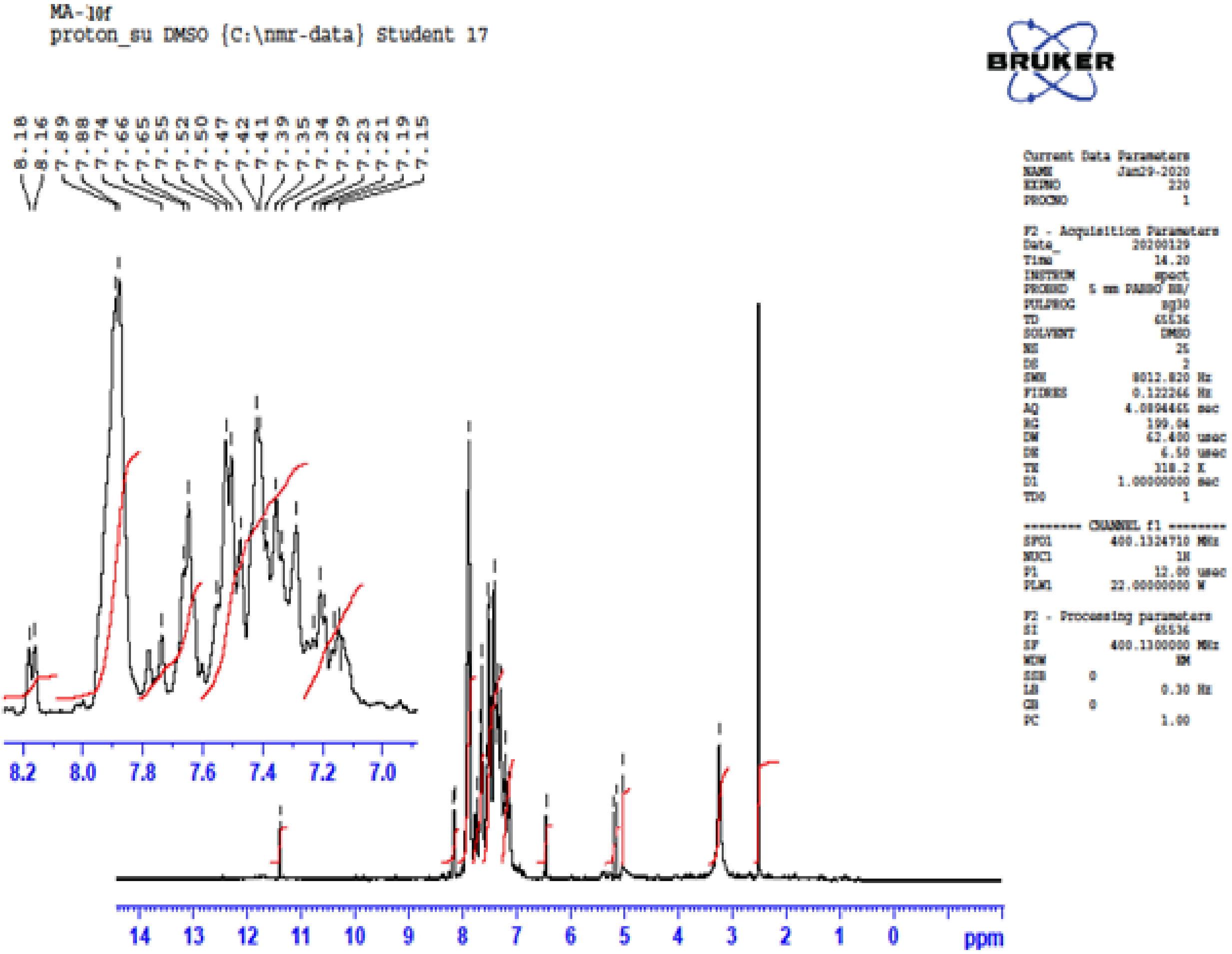

Figure S36: ^{1}H NMR Spectroscopy of Compound **10g**

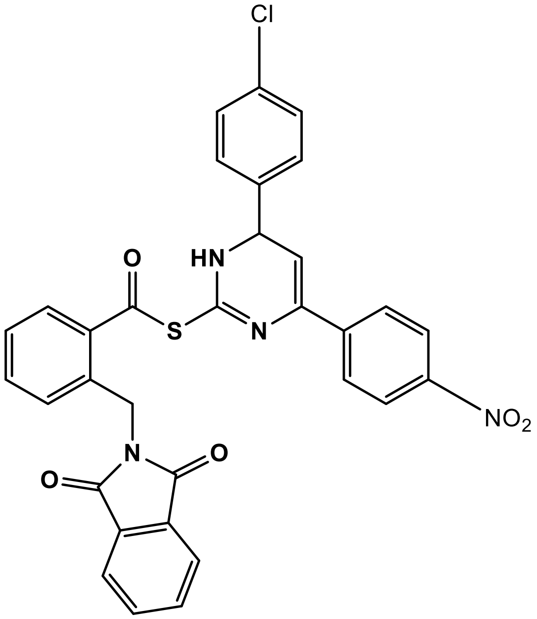

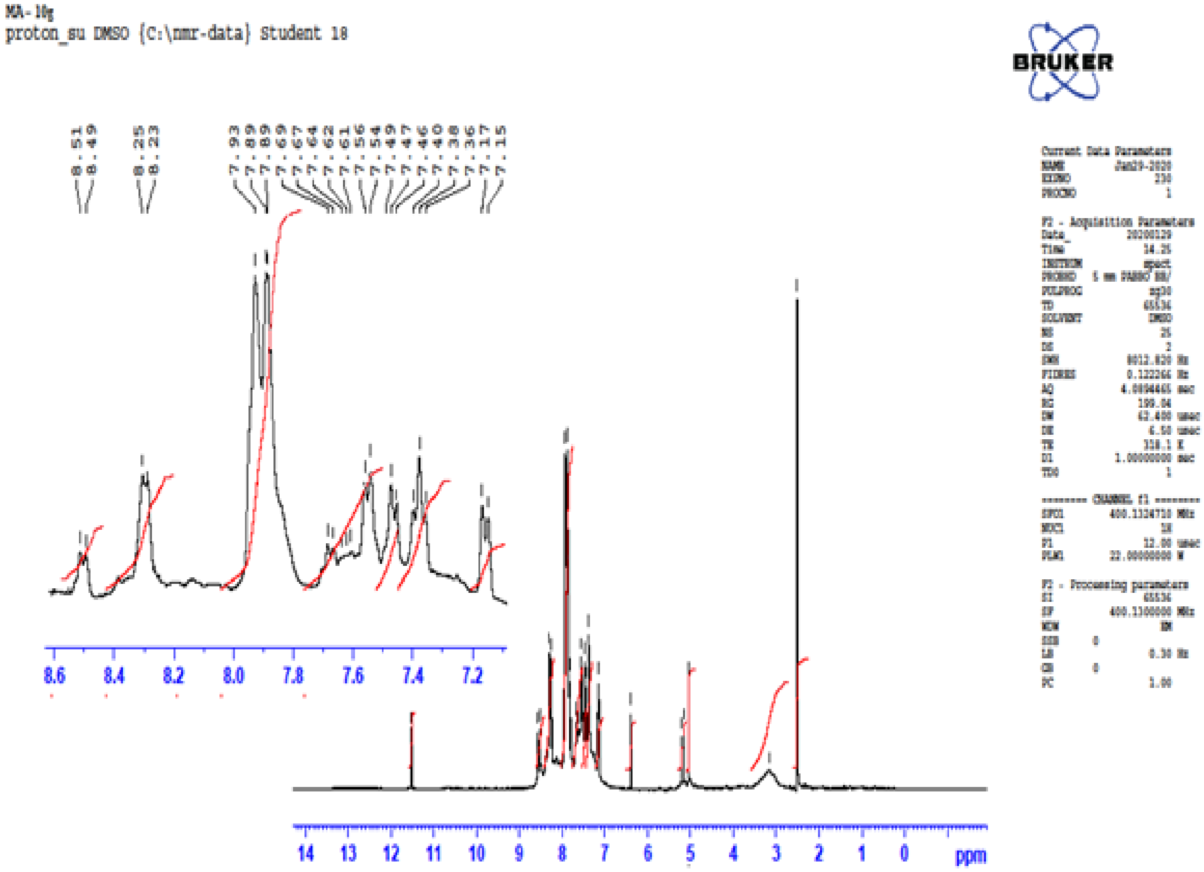

Figure S37: ^{1}H NMR Spectroscopy of Compound **10h**

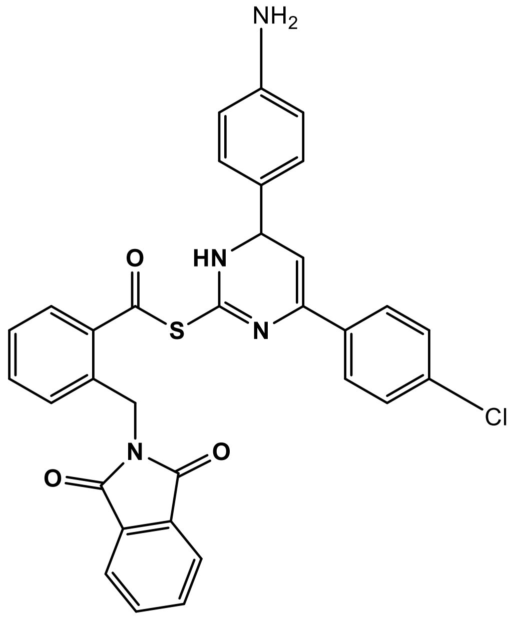

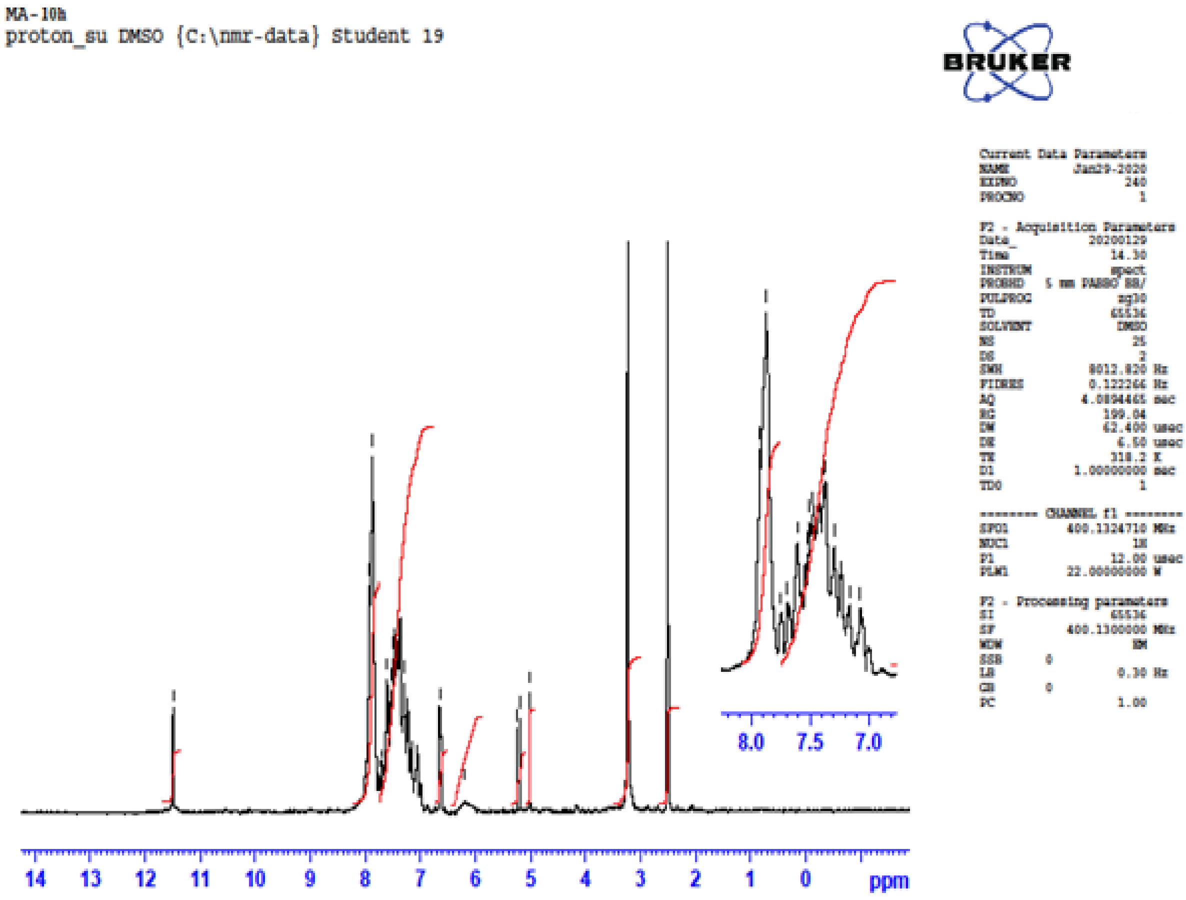

Figure S38: ^{1}H NMR Spectroscopy of Compound **10i**

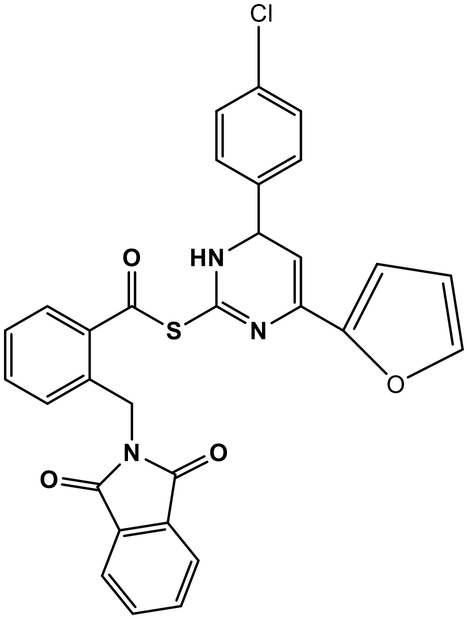

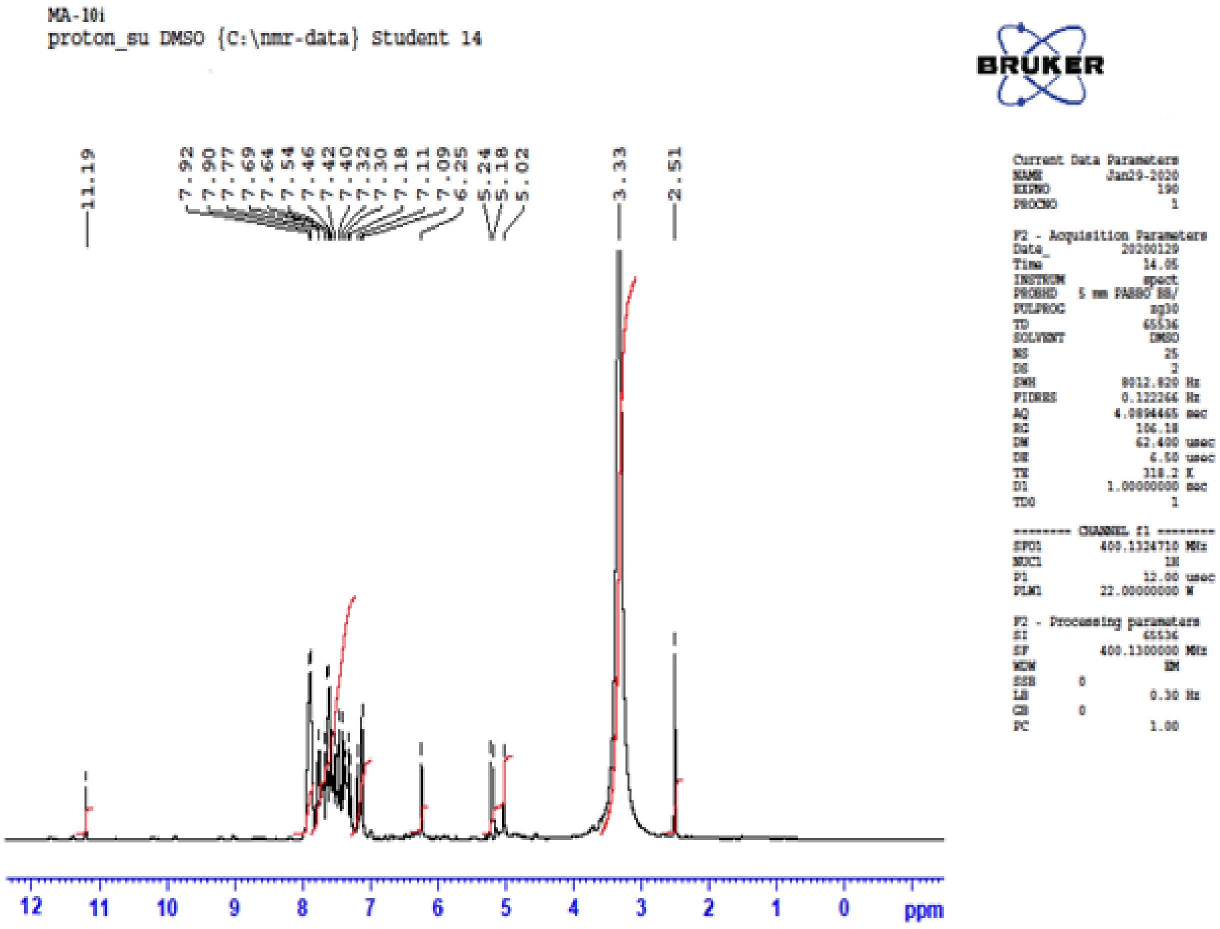

Figure S39: ^{1}H NMR Spectroscopy of Compound **10j**

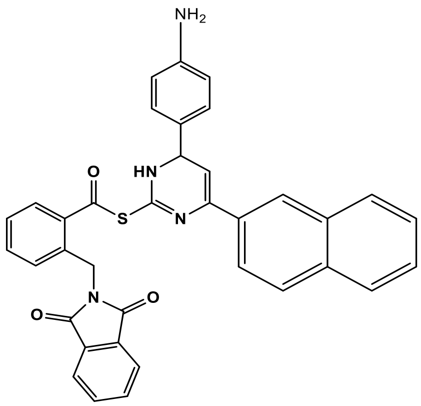

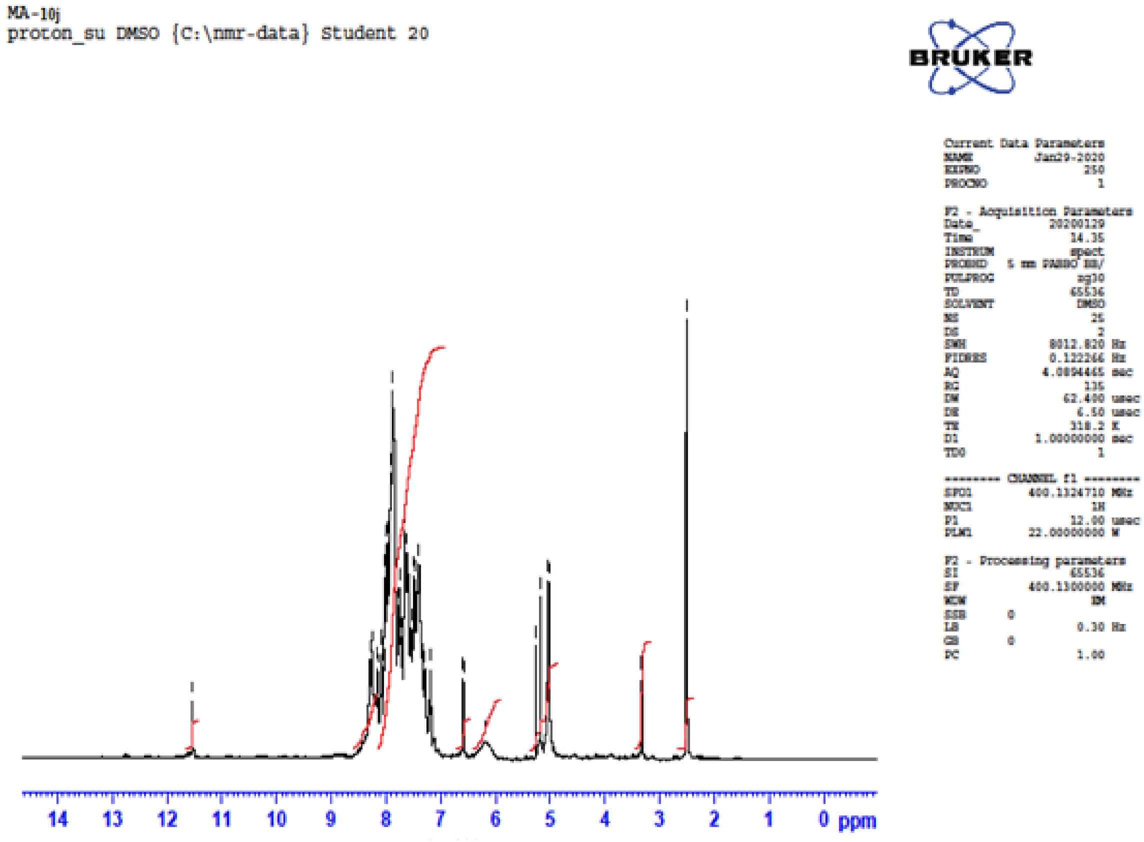

^{13}C NMR Spectroscopy

Figure S40: ^{13}C NMR Spectroscopy of Compound **5a**

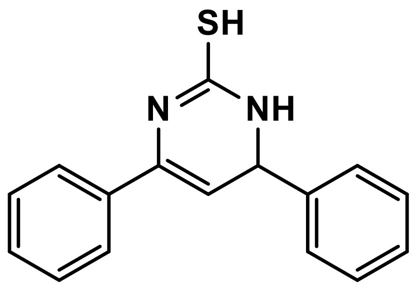

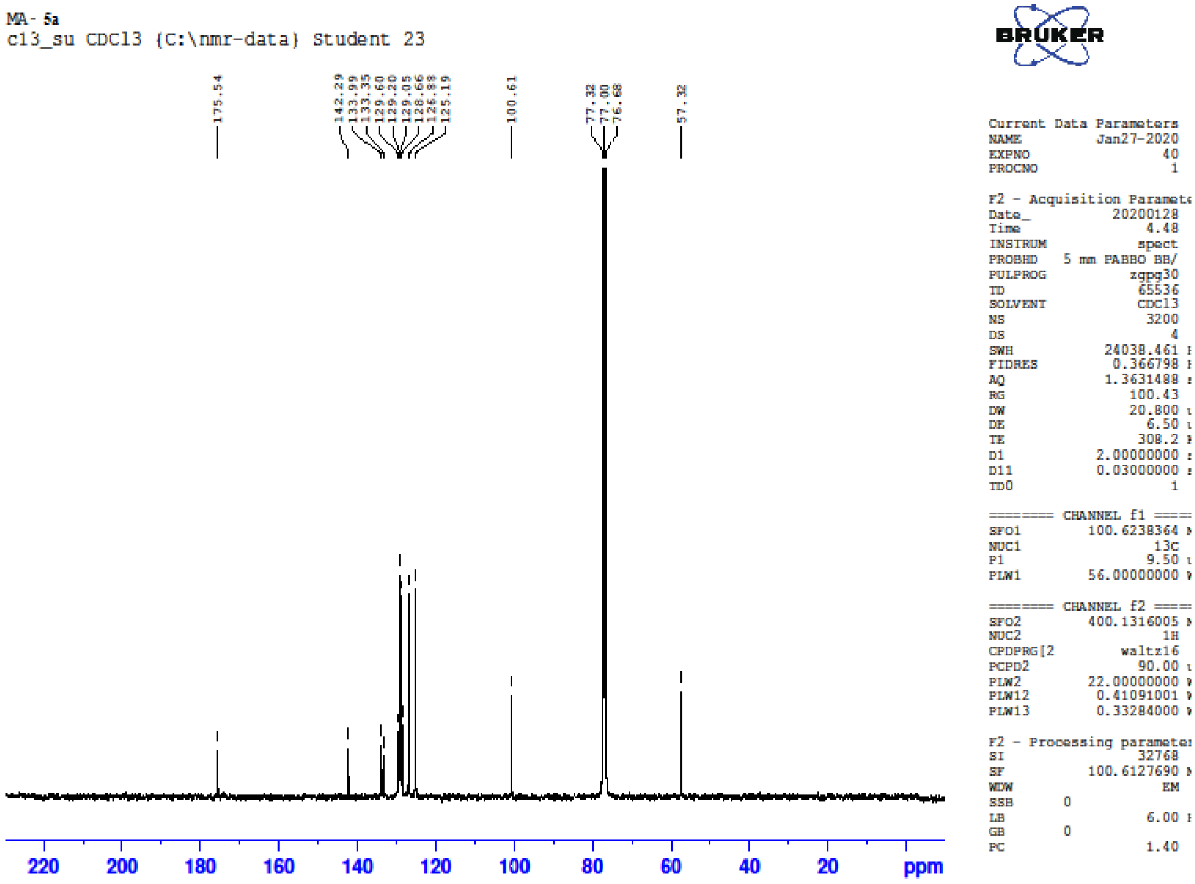

Figure S41: ^{13}C NMR Spectroscopy of Compound **5d**

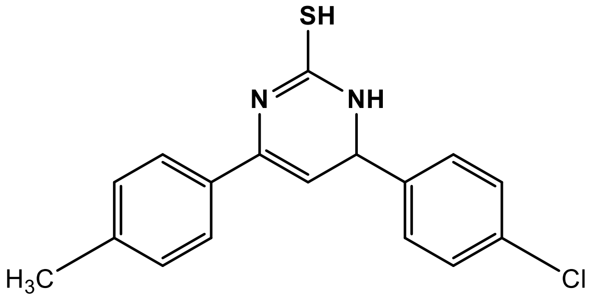

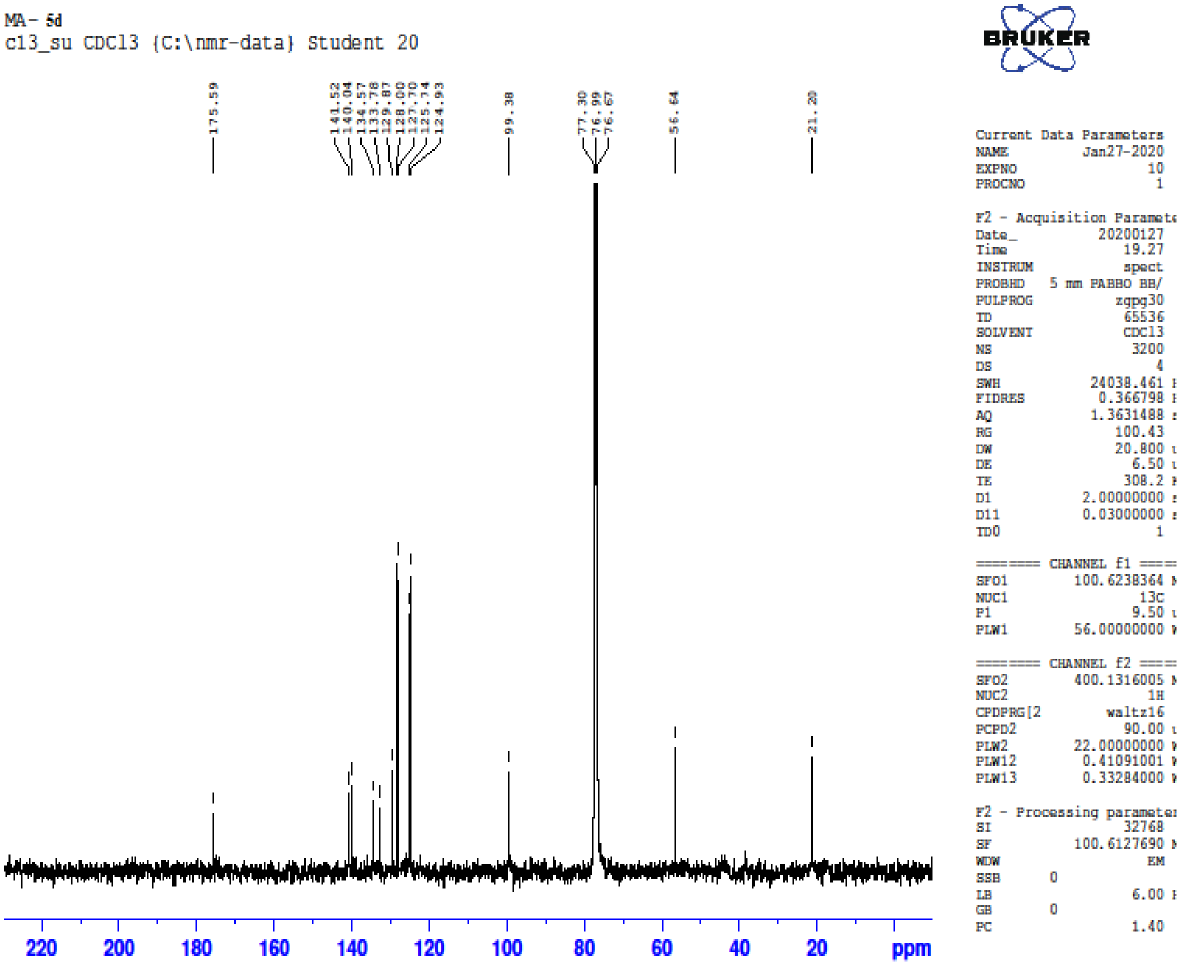

Figure S42: ^{13}C NMR Spectroscopy of Compound **5g**

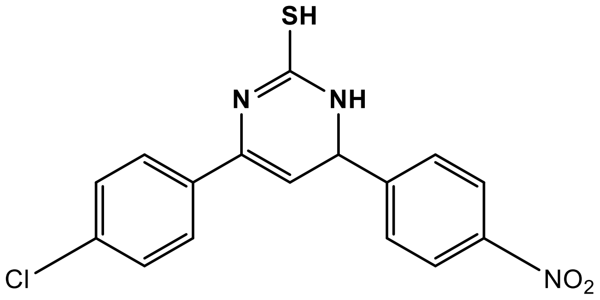

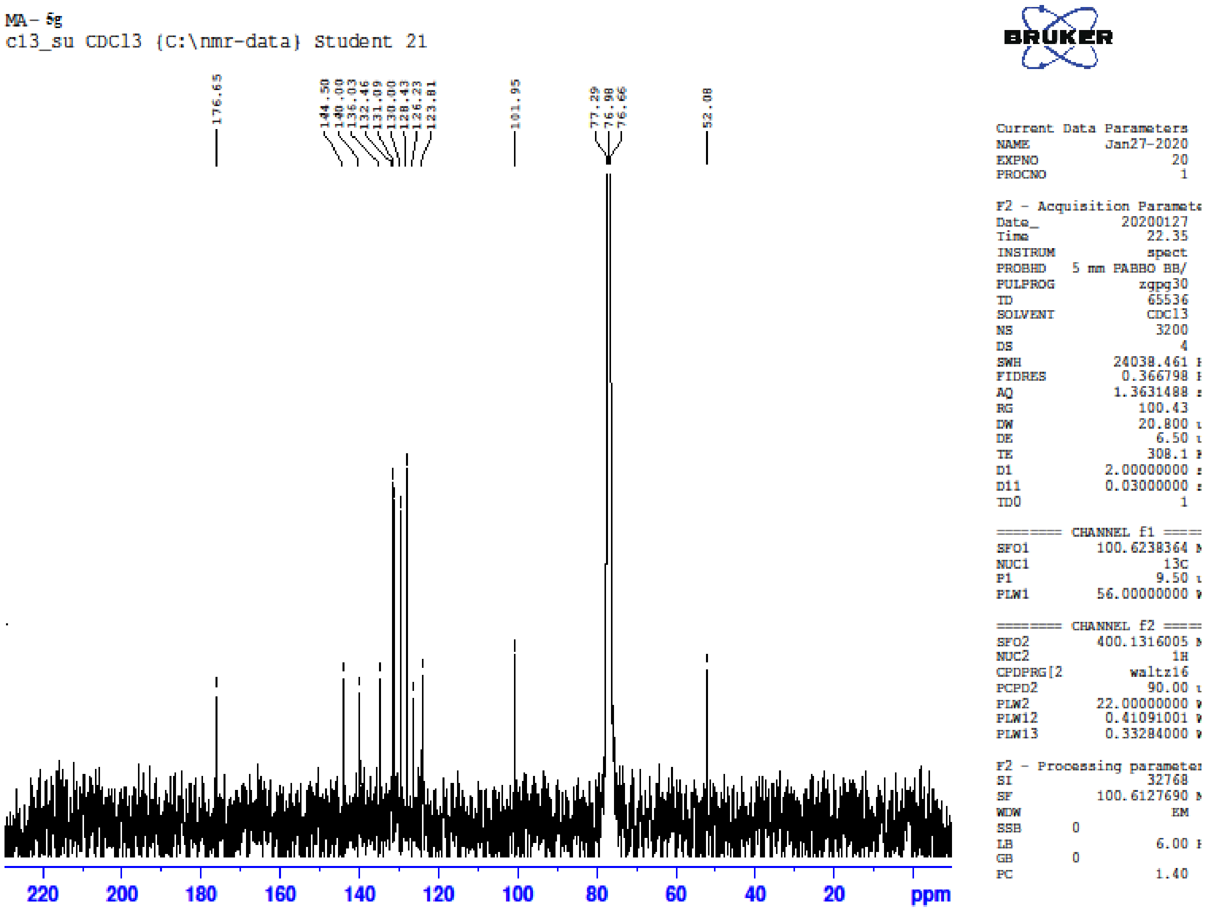

Figure S43: ^{13}C NMR Spectroscopy of Compound **5h**

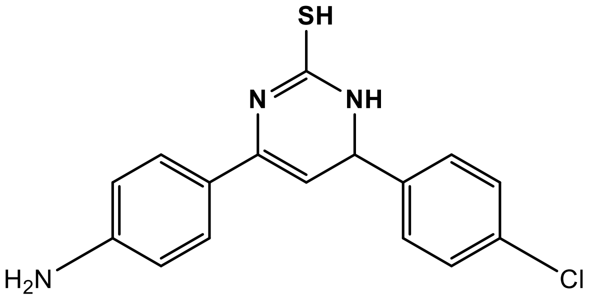

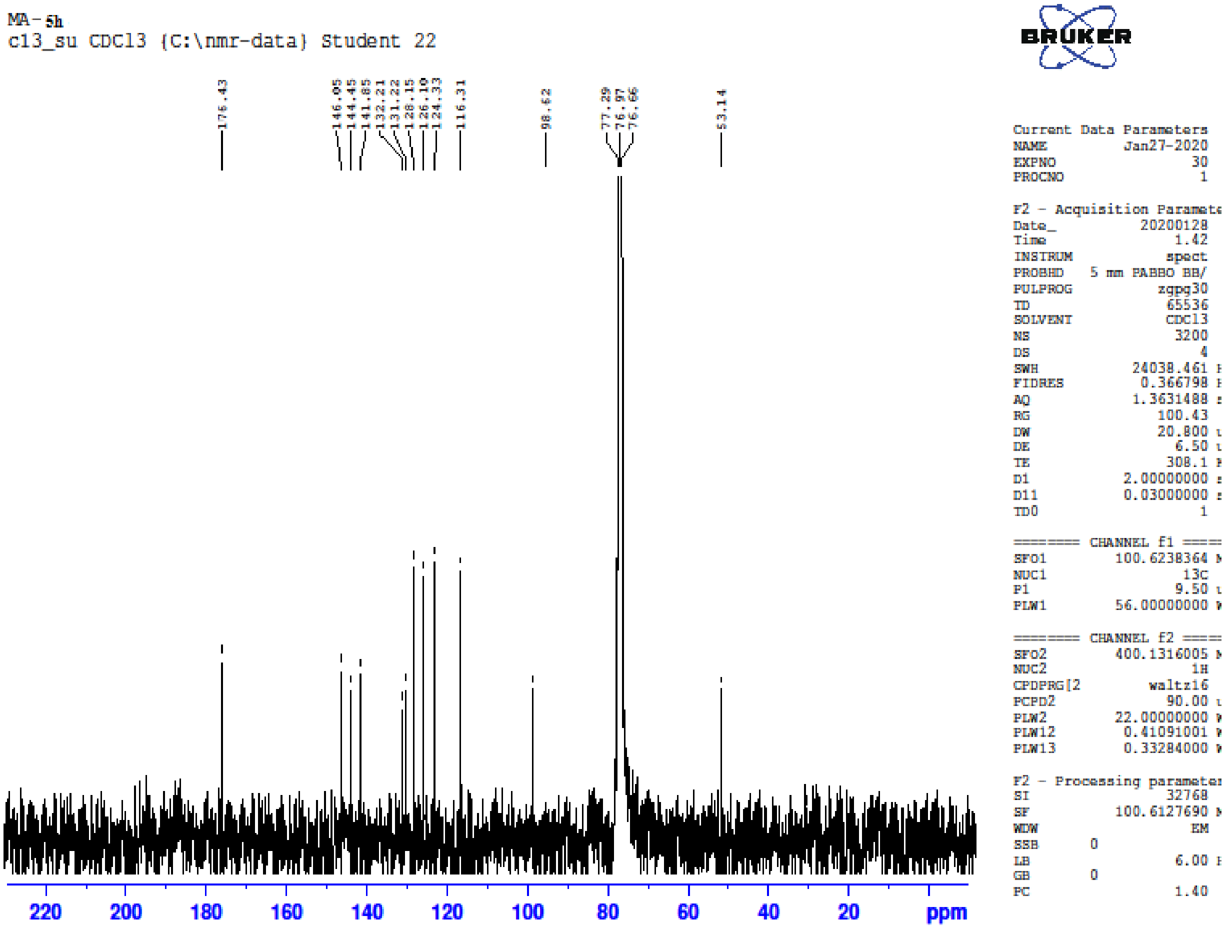

Figure S44: ^{13}C NMR Spectroscopy of Compound **10a**

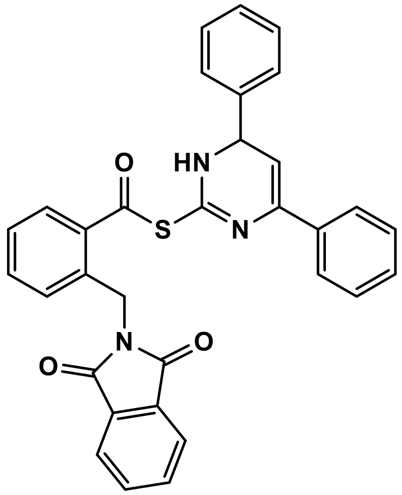

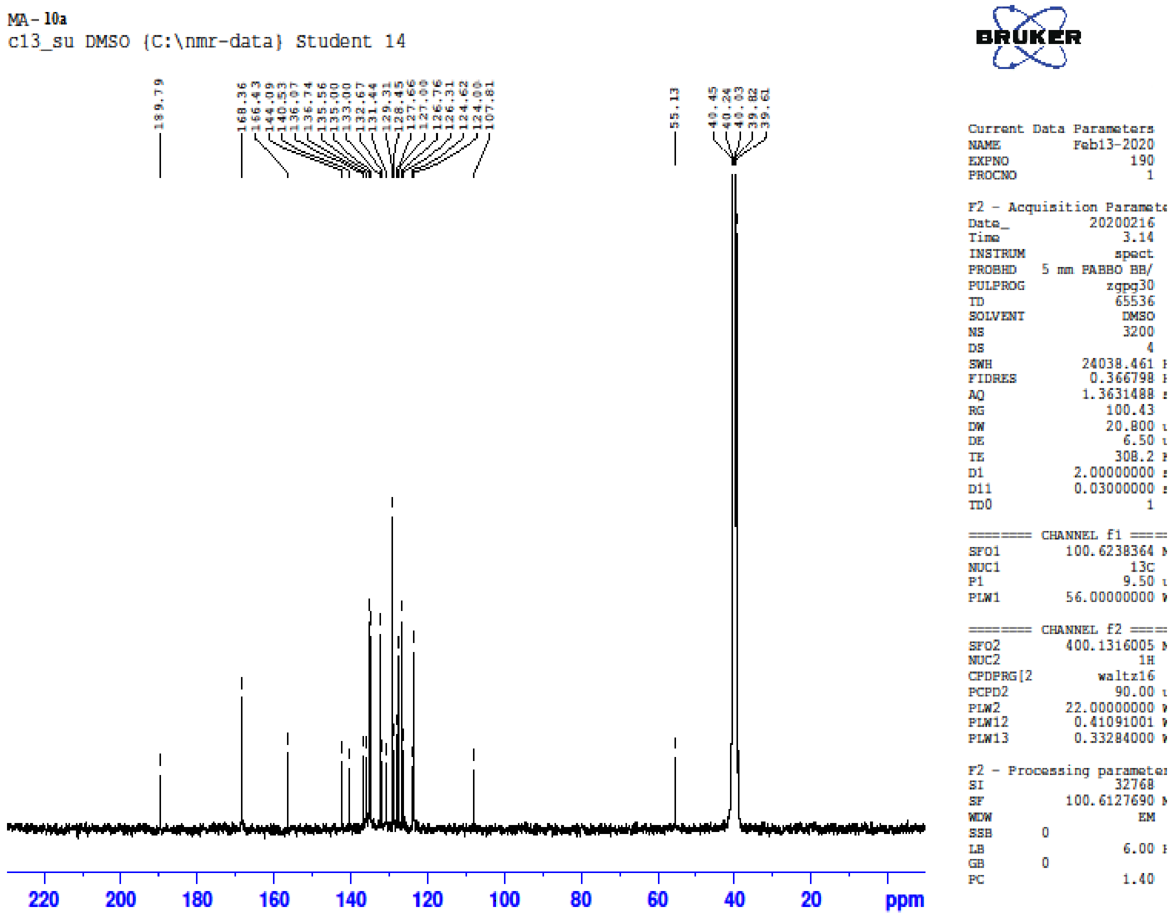

Figure S45: ^{13}C NMR Spectroscopy of Compound **10b**

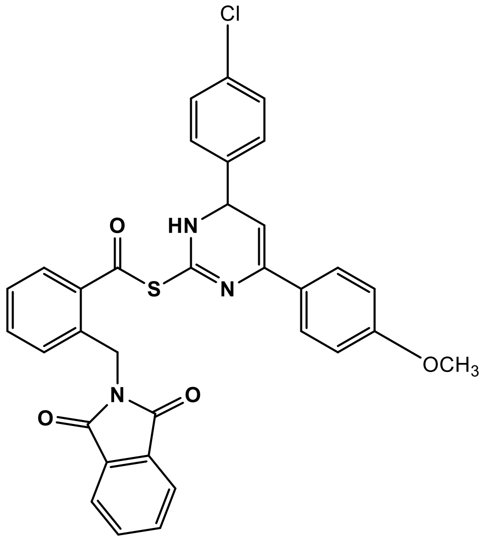

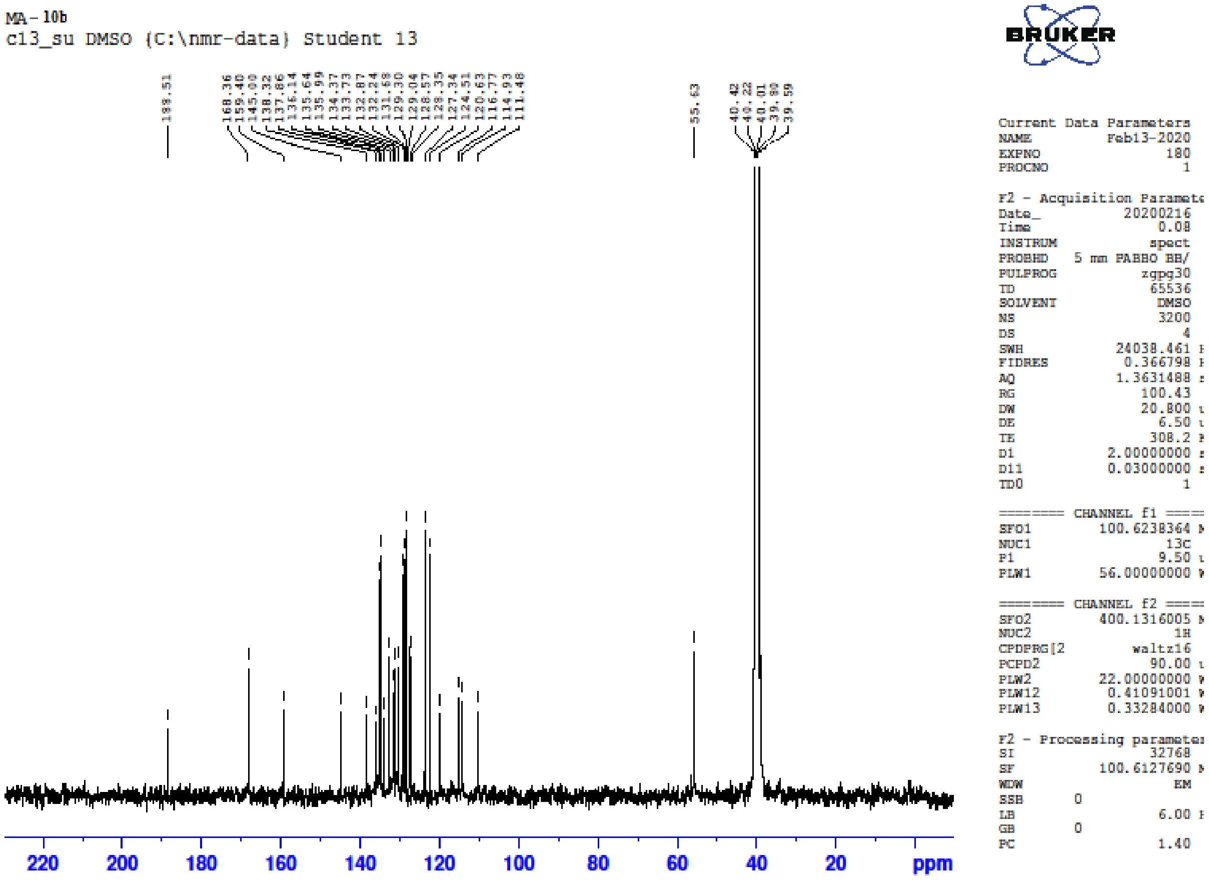

Figure S46: ^{13}C NMR Spectroscopy of Compound **10d**

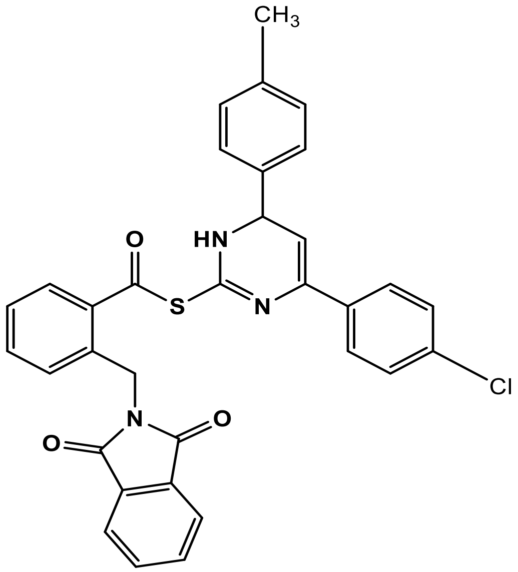

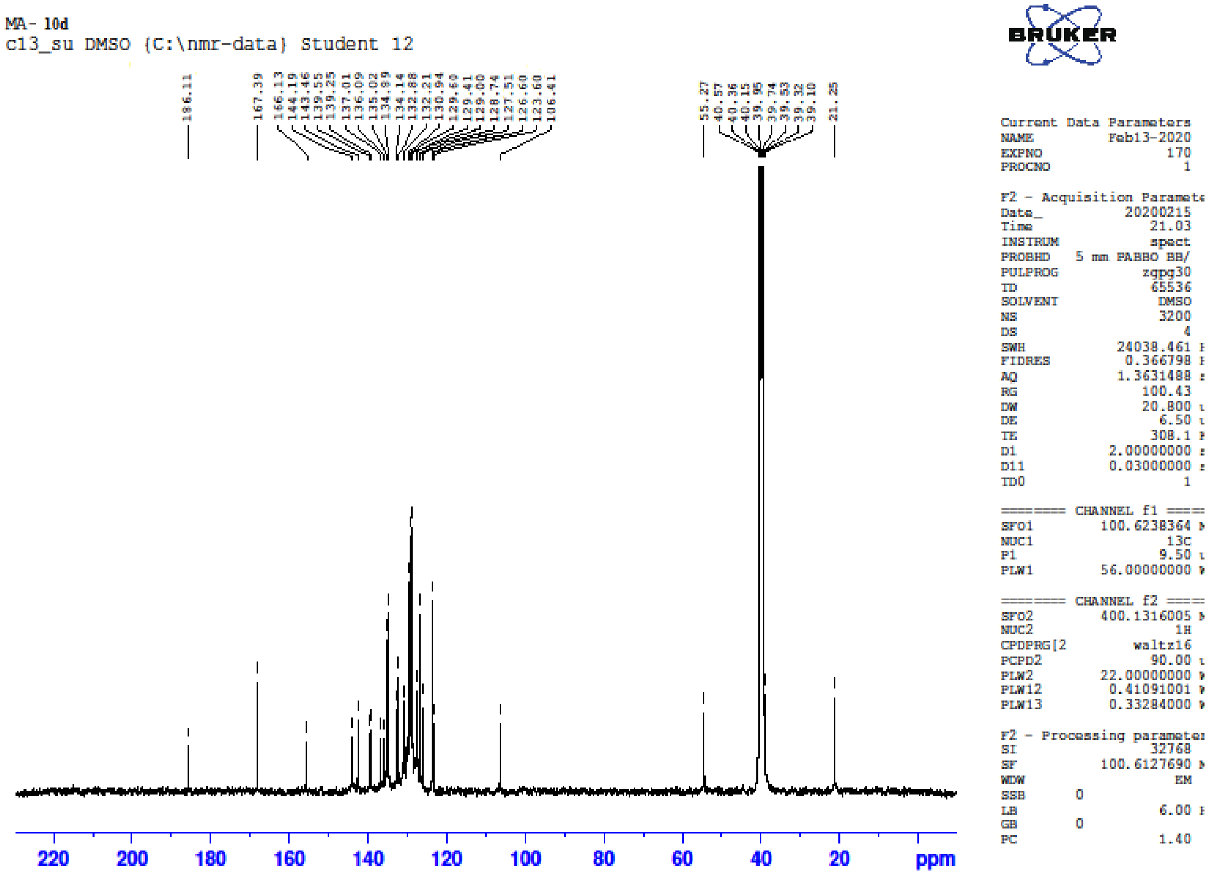

Figure S47: ^{13}C NMR Spectroscopy of Compound **10g**

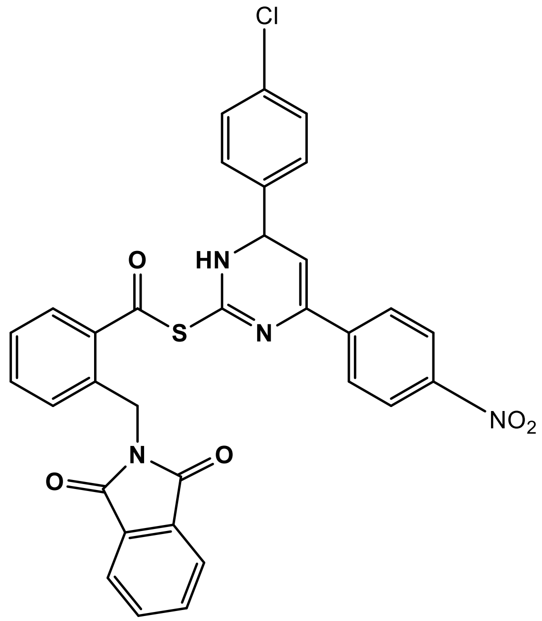

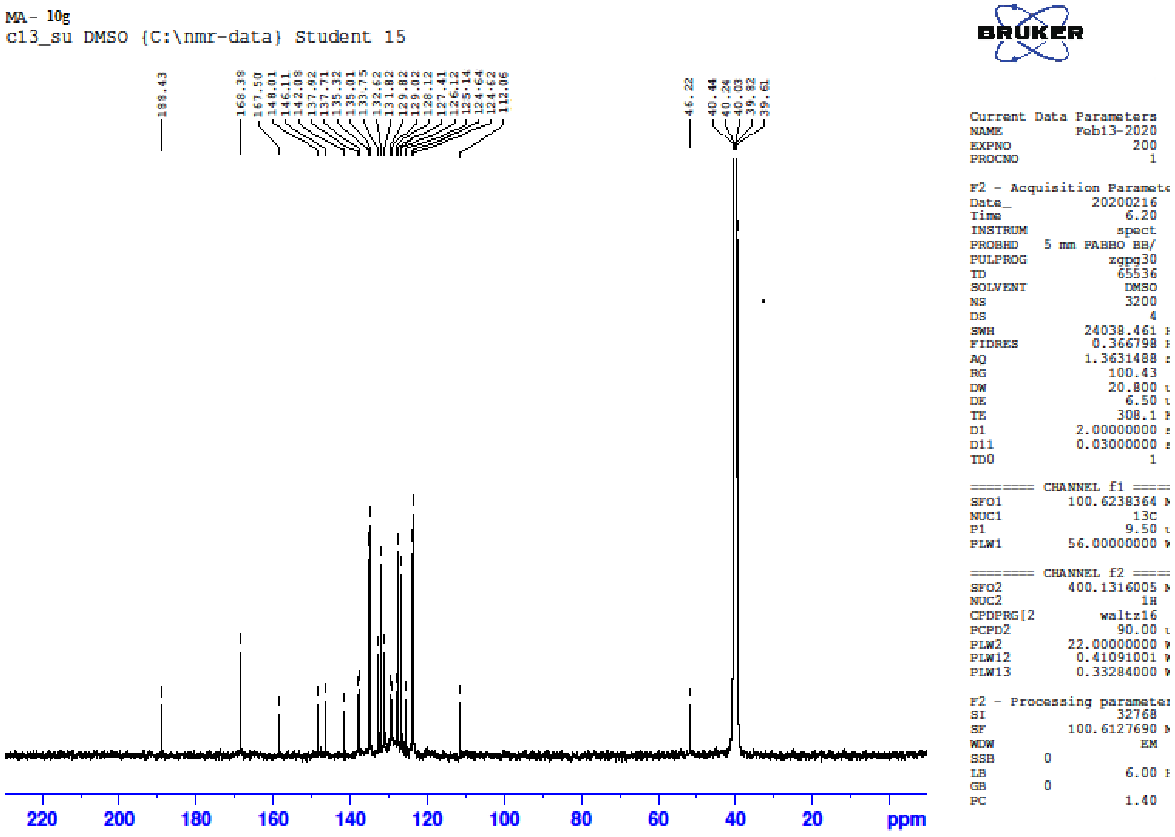

Figure S48: ^{13}C NMR Spectroscopy of Compound **10j**

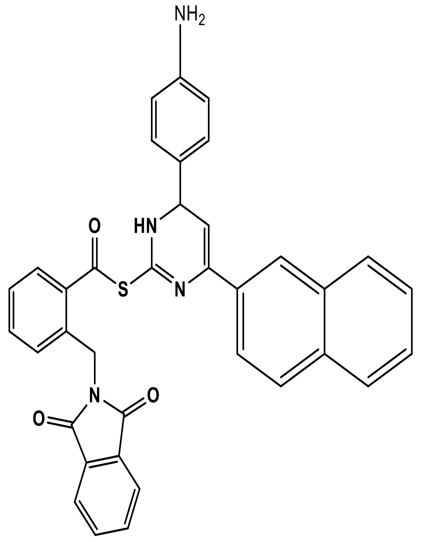

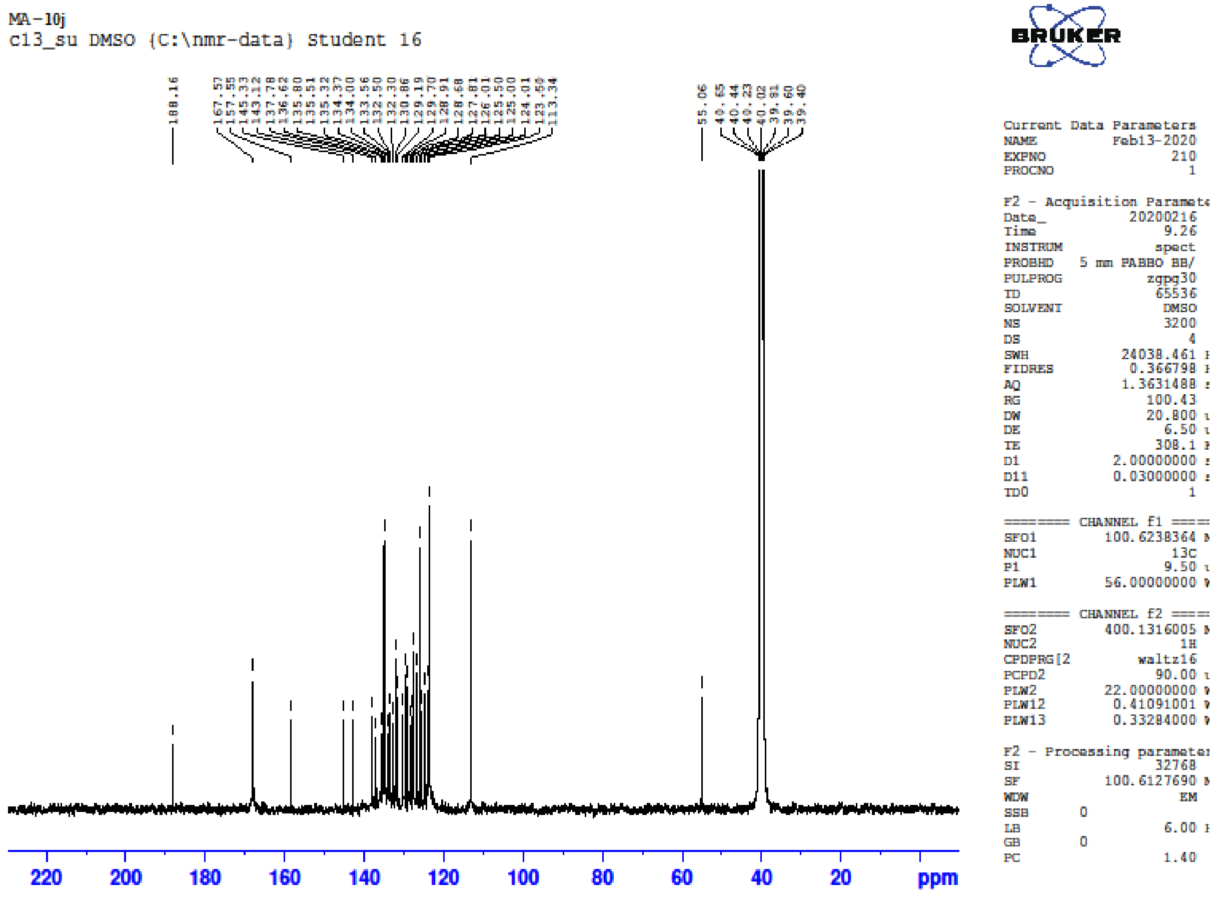

Mass Spectroscopy

Figure S49: Mass Spectroscopy of Compound **5b**

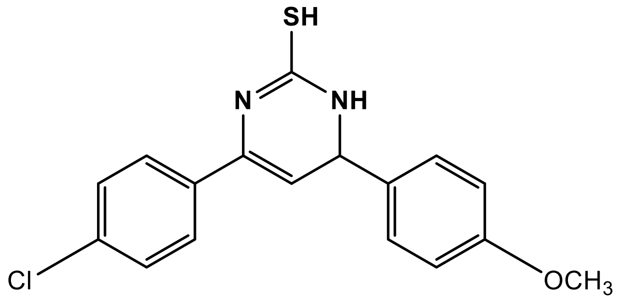

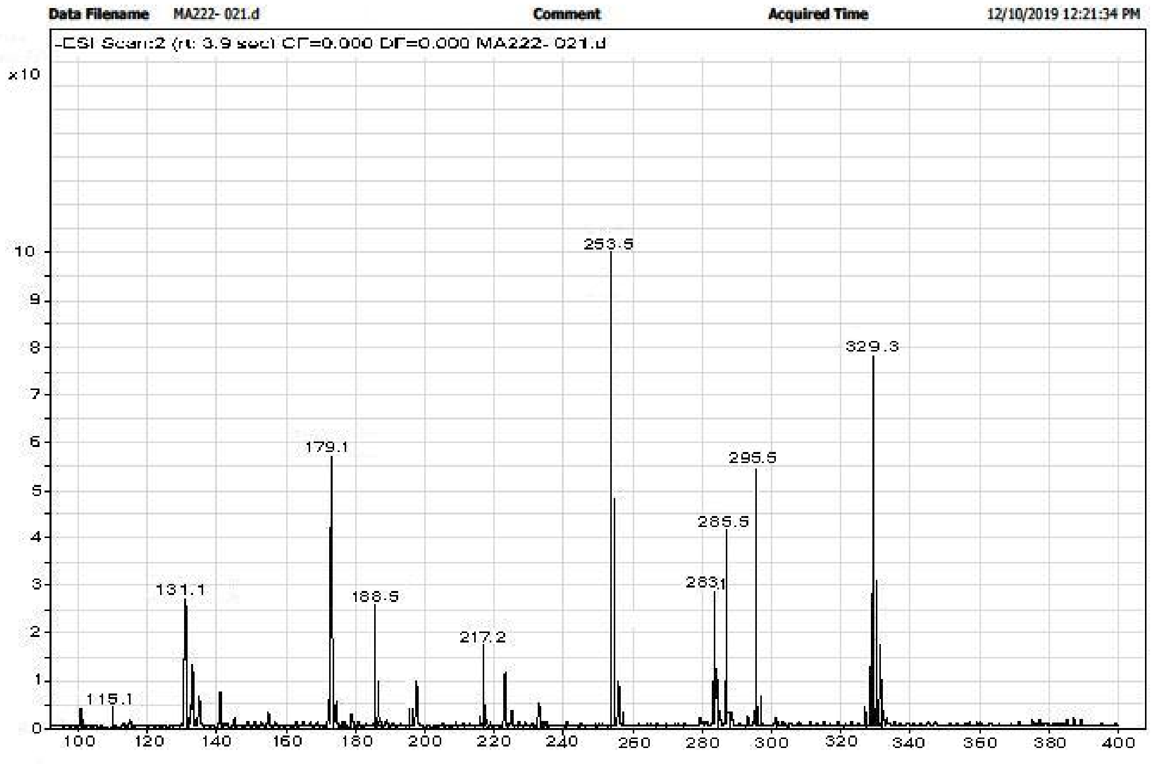

Figure S50: Mass Spectroscopy of Compound **5c**

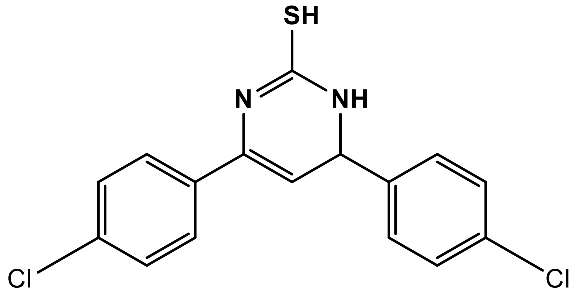

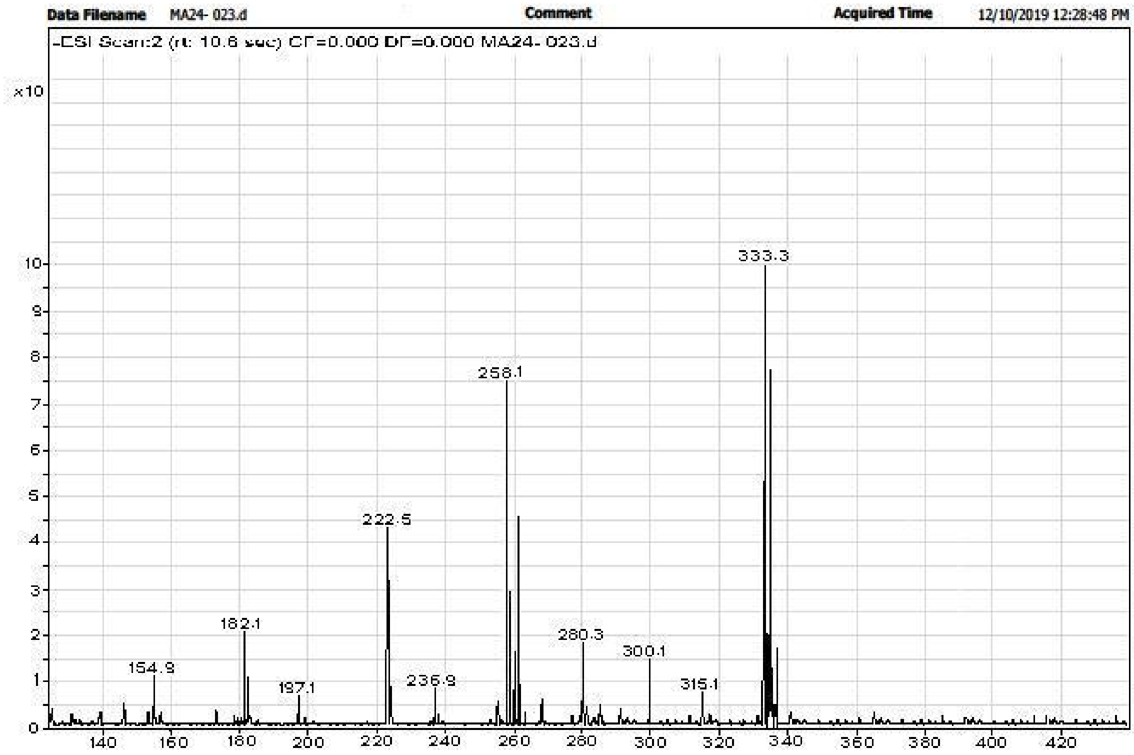

Figure S51: Mass Spectroscopy of Compound **5d**

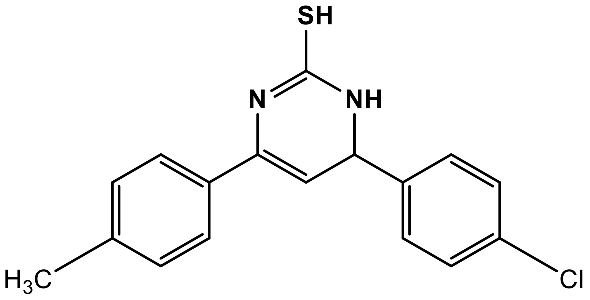

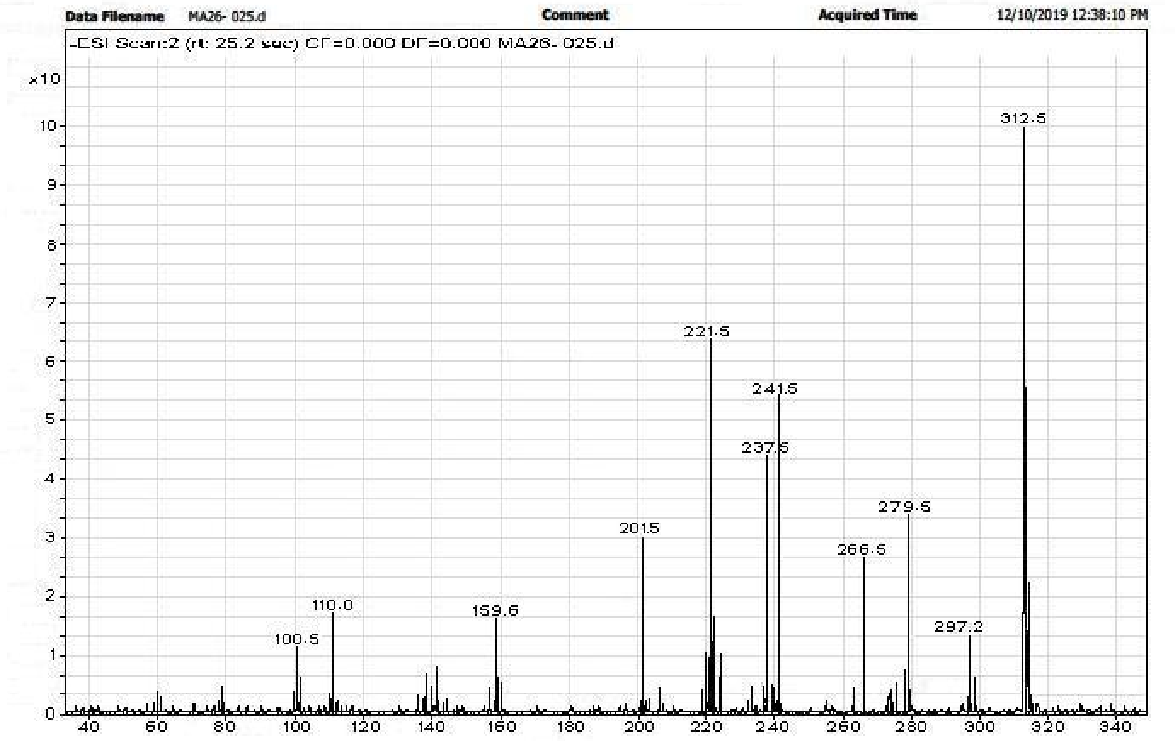

Figure S52: Mass Spectroscopy of Compound **5e**

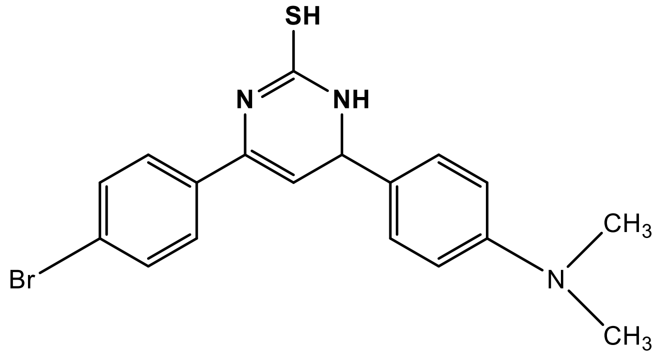

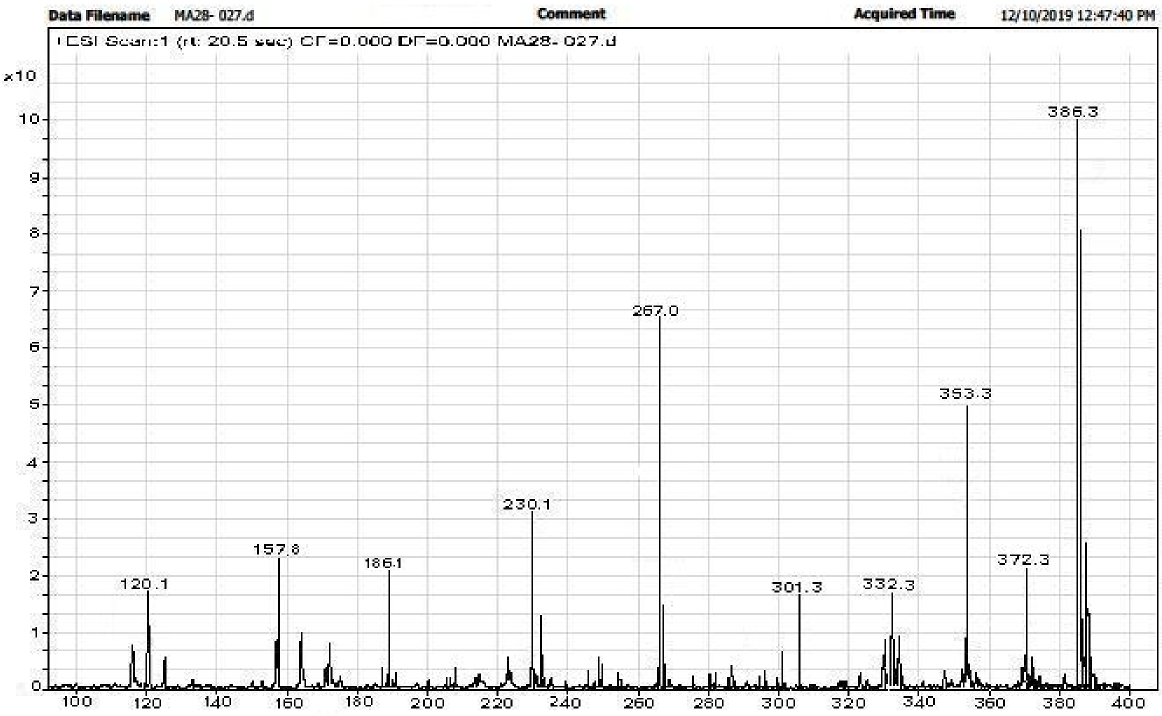

Figure S53: Mass Spectroscopy of Compound **5f**

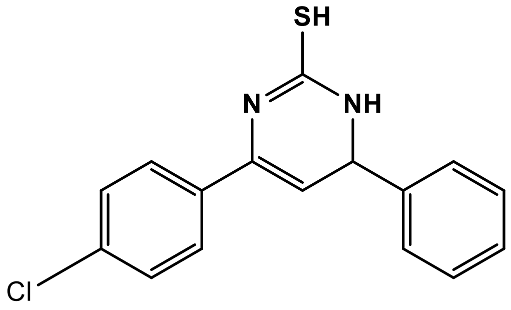

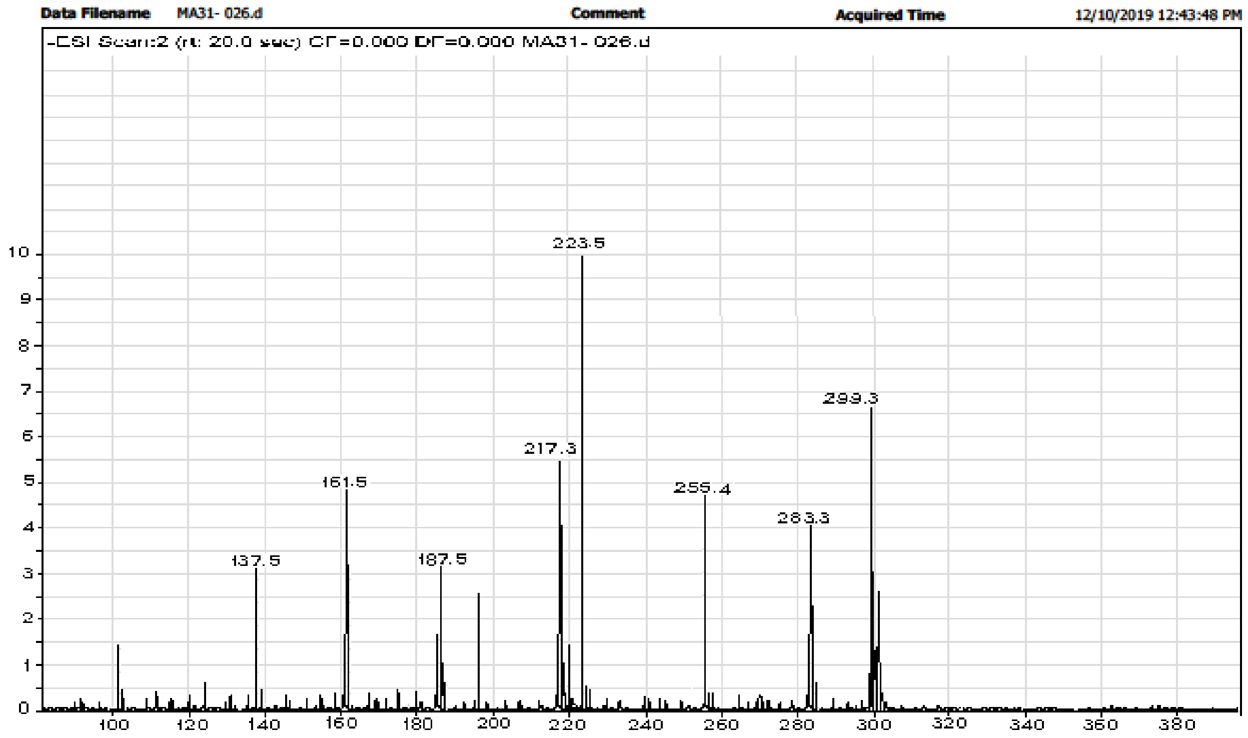

Figure S54: Mass Spectroscopy of Compound **5g**

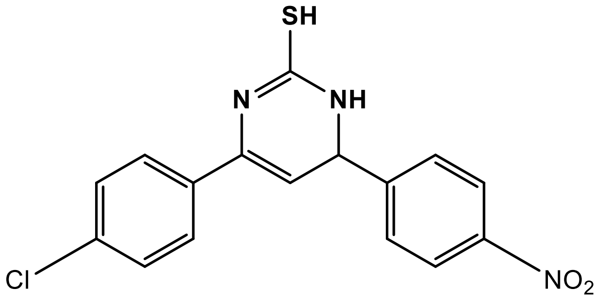

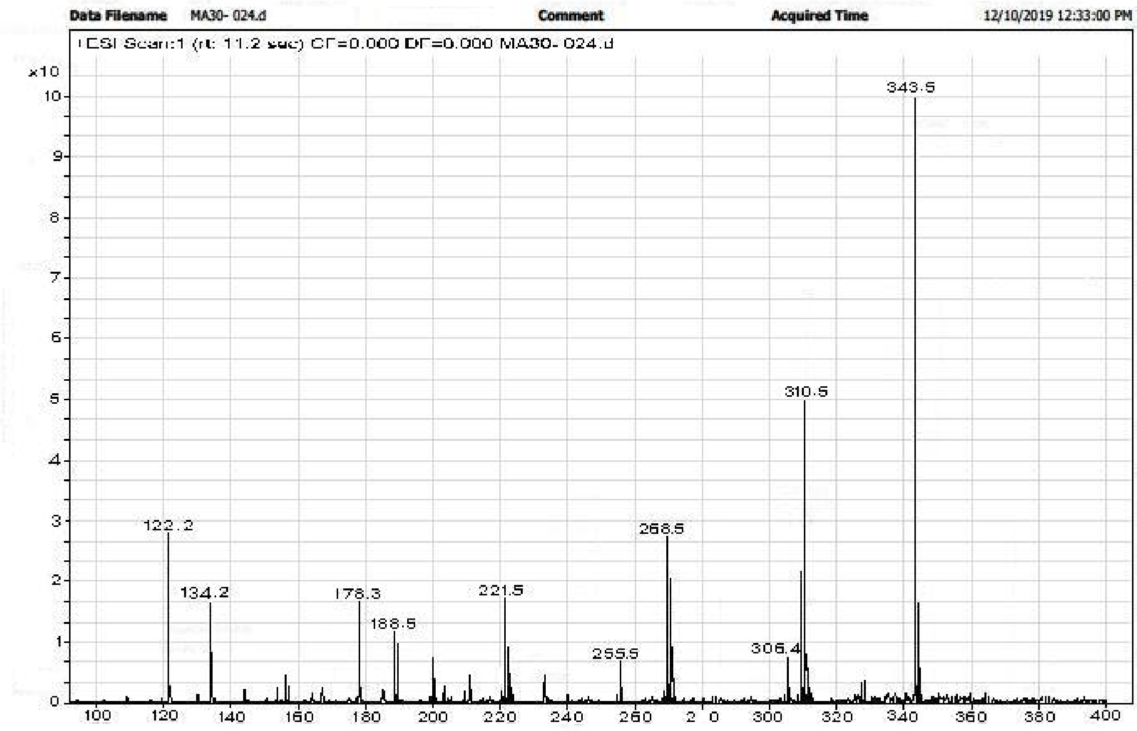

Figure S55: Mass Spectroscopy of Compound **5h**

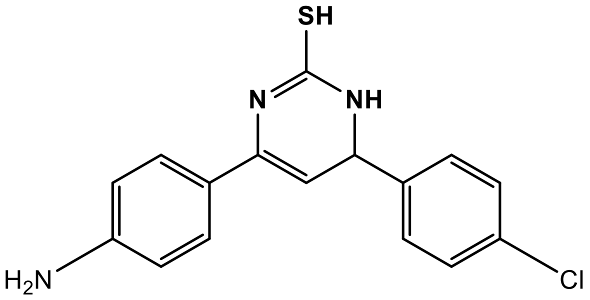

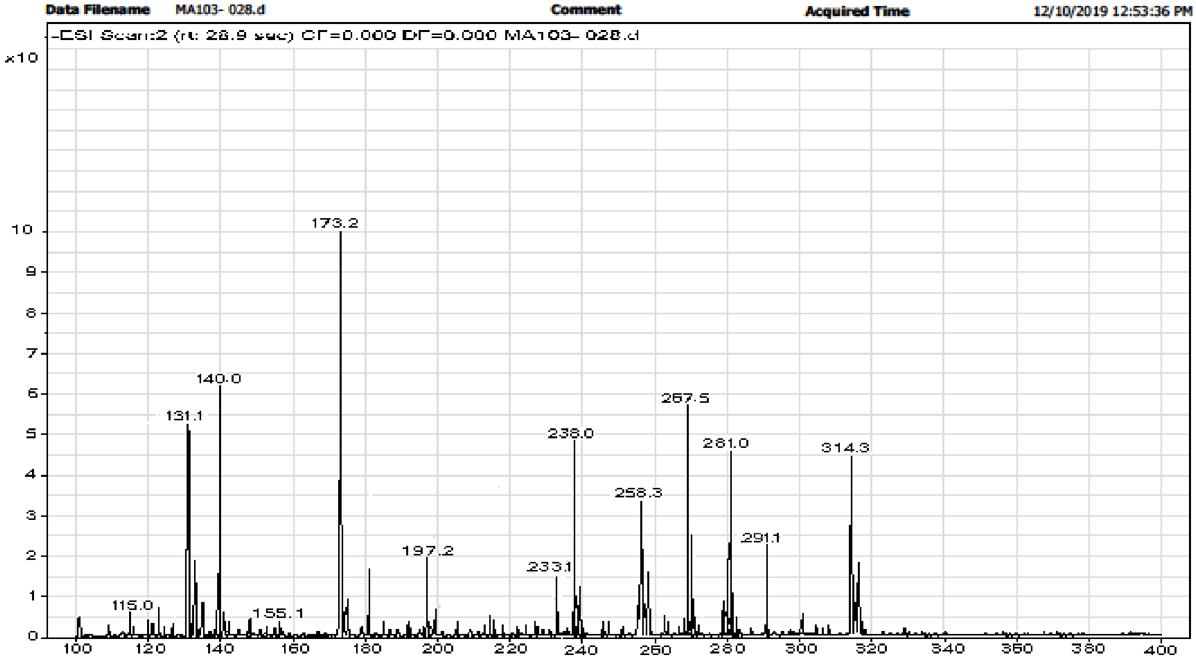

Figure S56: Mass Spectroscopy of Compound **5i**

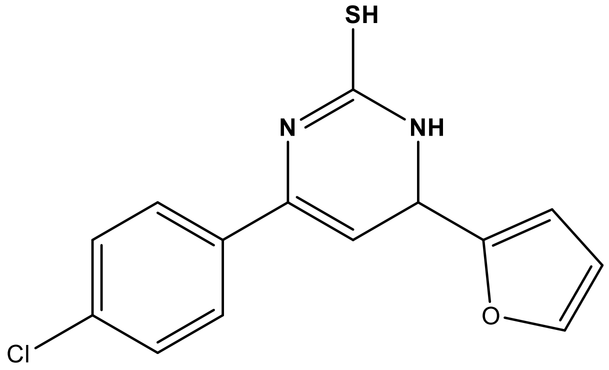

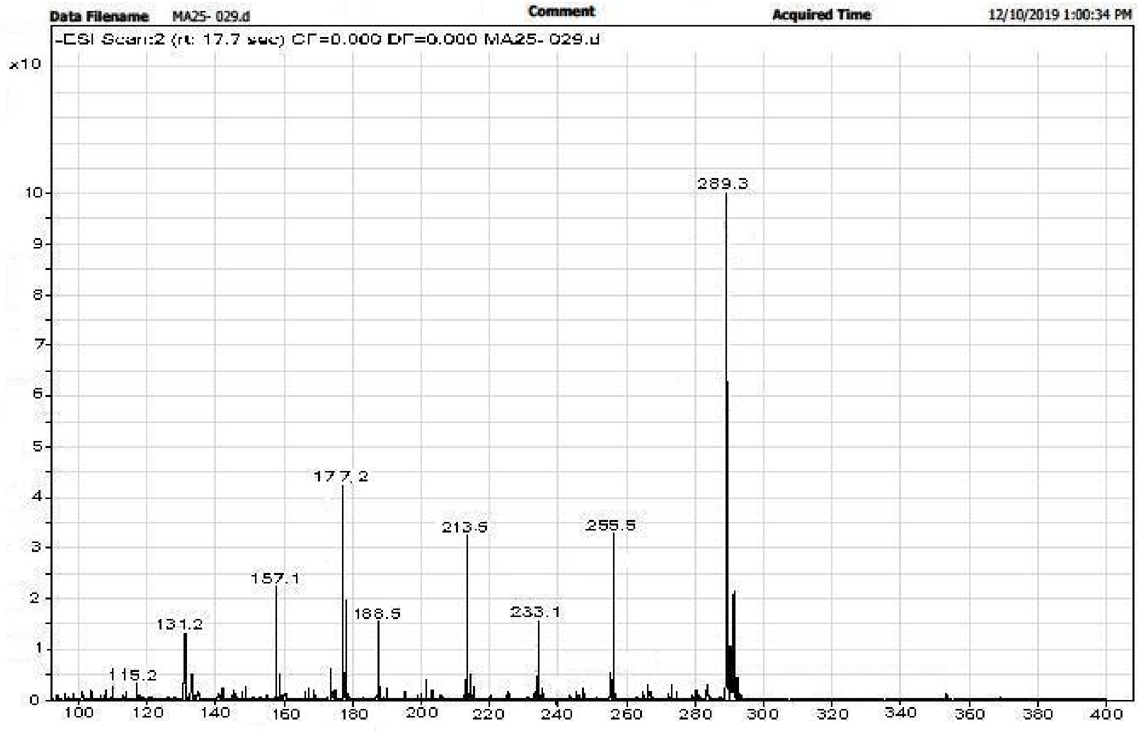

Figure S57: Mass Spectroscopy of Compound **5j**

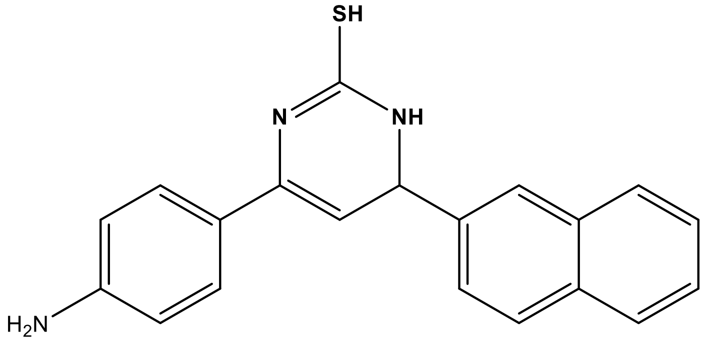

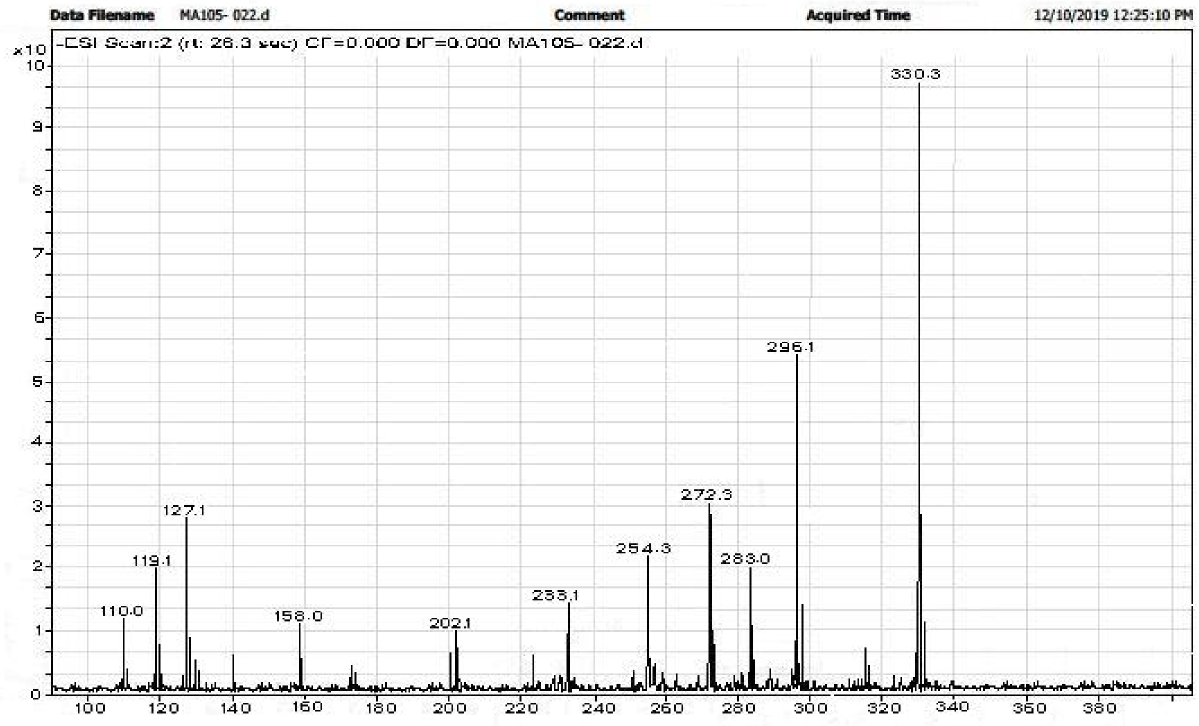

Figure S58: Mass Spectroscopy of Compound **10a**

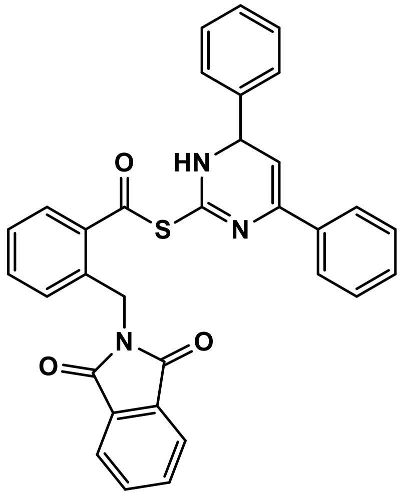

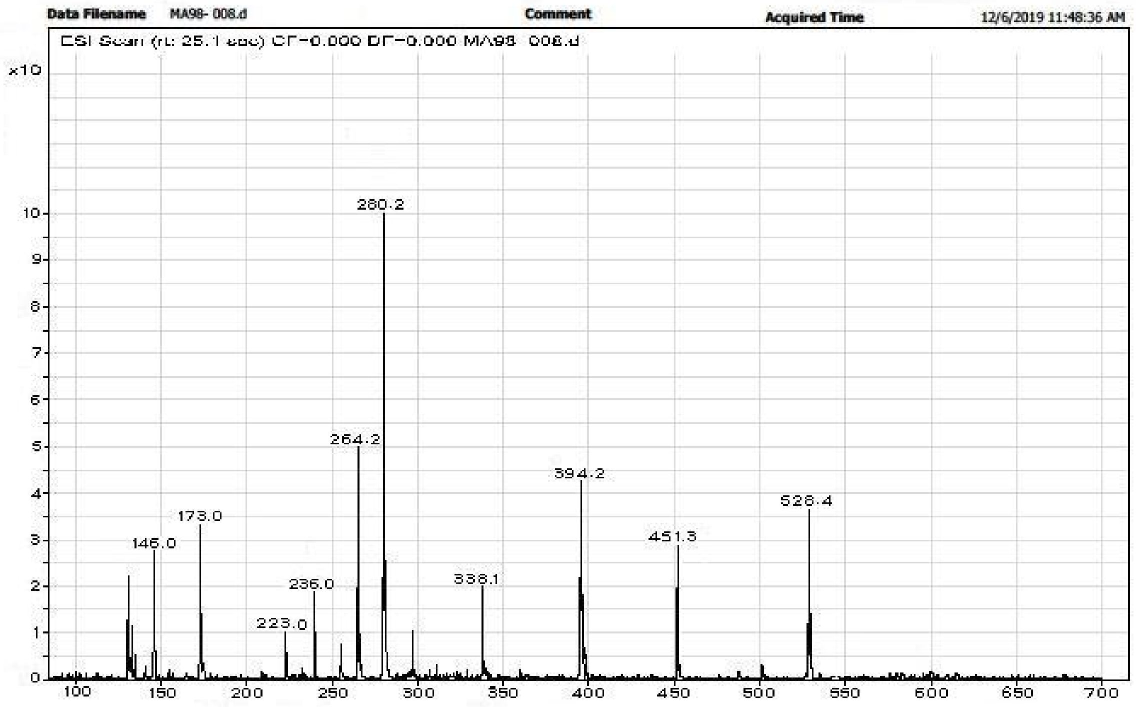

Figure S59: Mass Spectroscopy of Compound **10b**

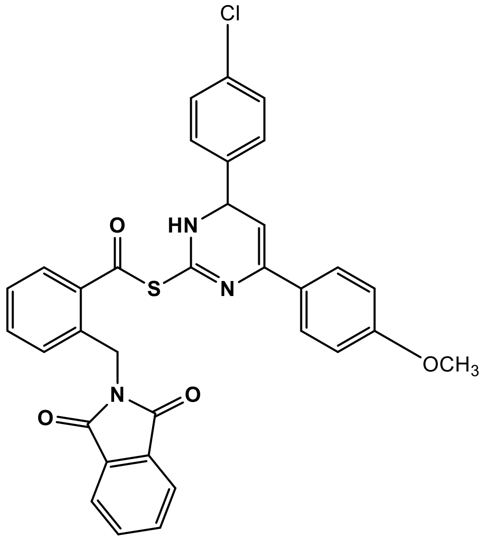

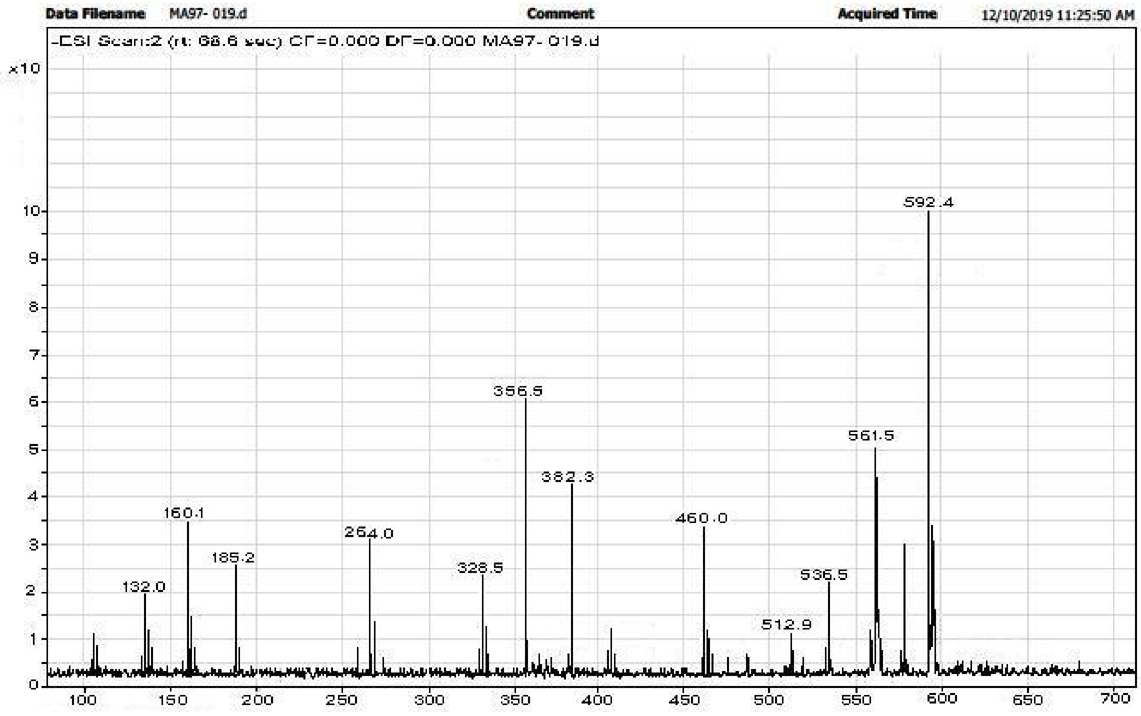

Figure S60: Mass Spectroscopy of Compound **10c**

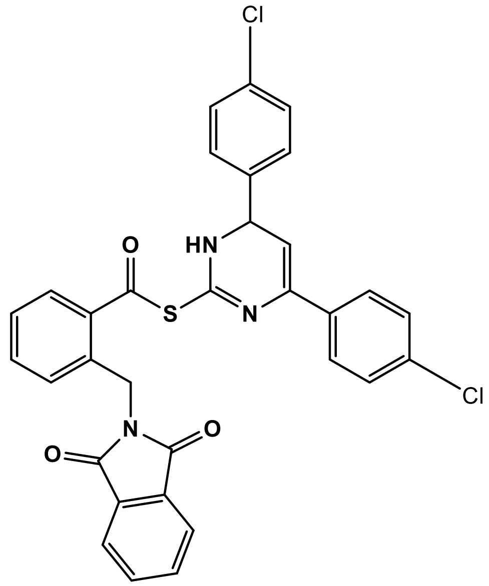

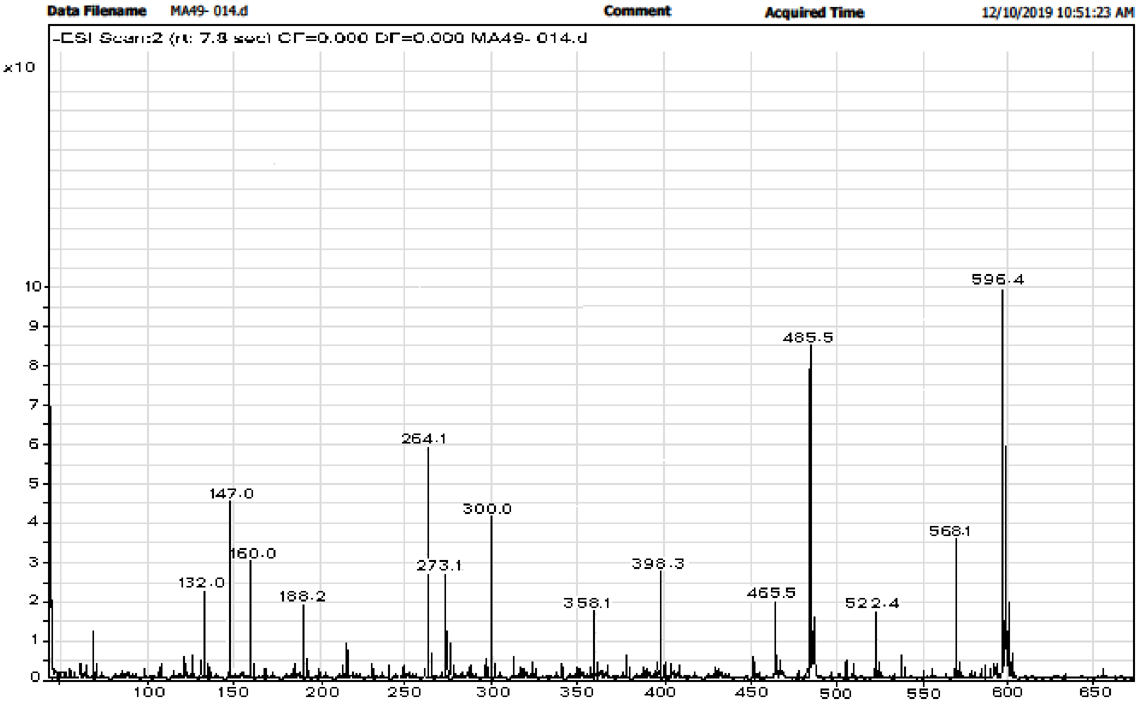

Figure S61: Mass Spectroscopy of Compound **10d**

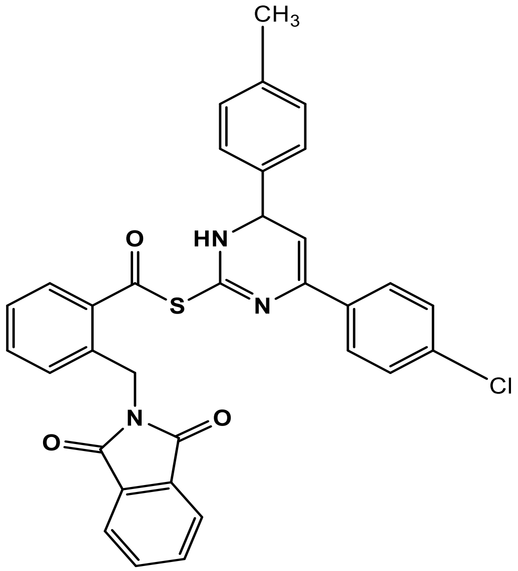

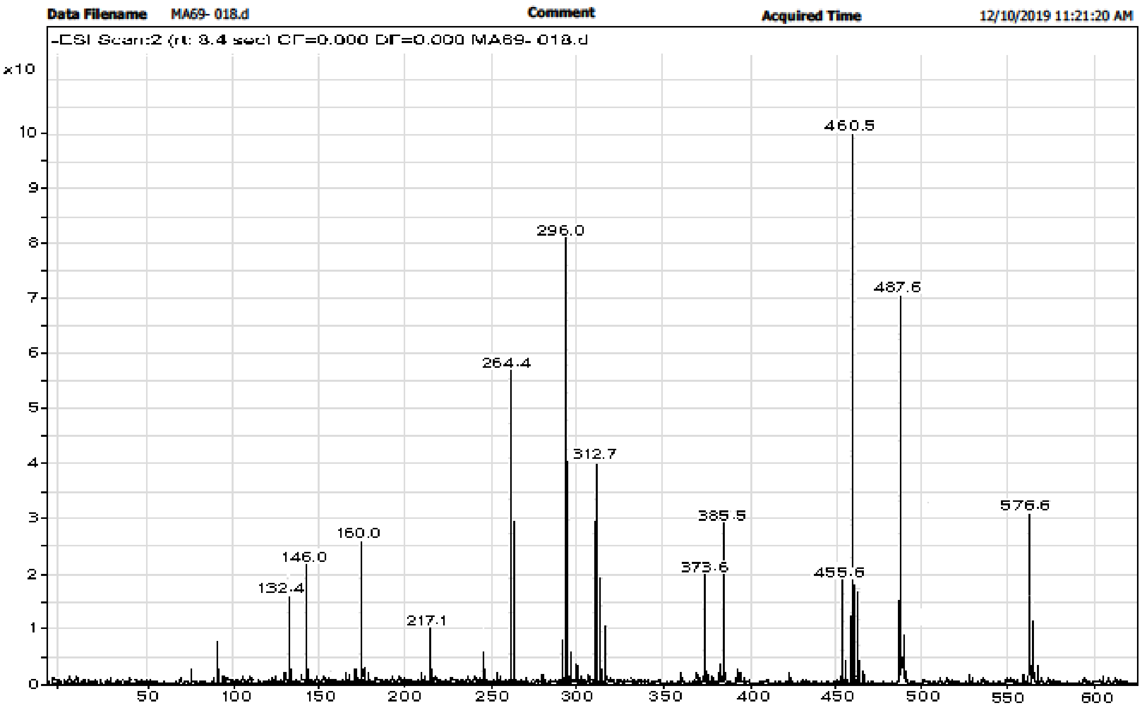

Figure S62: Mass Spectroscopy of Compound **10e**

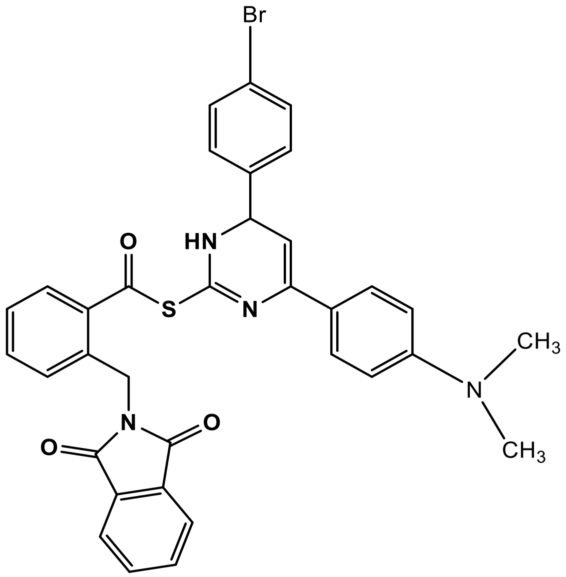

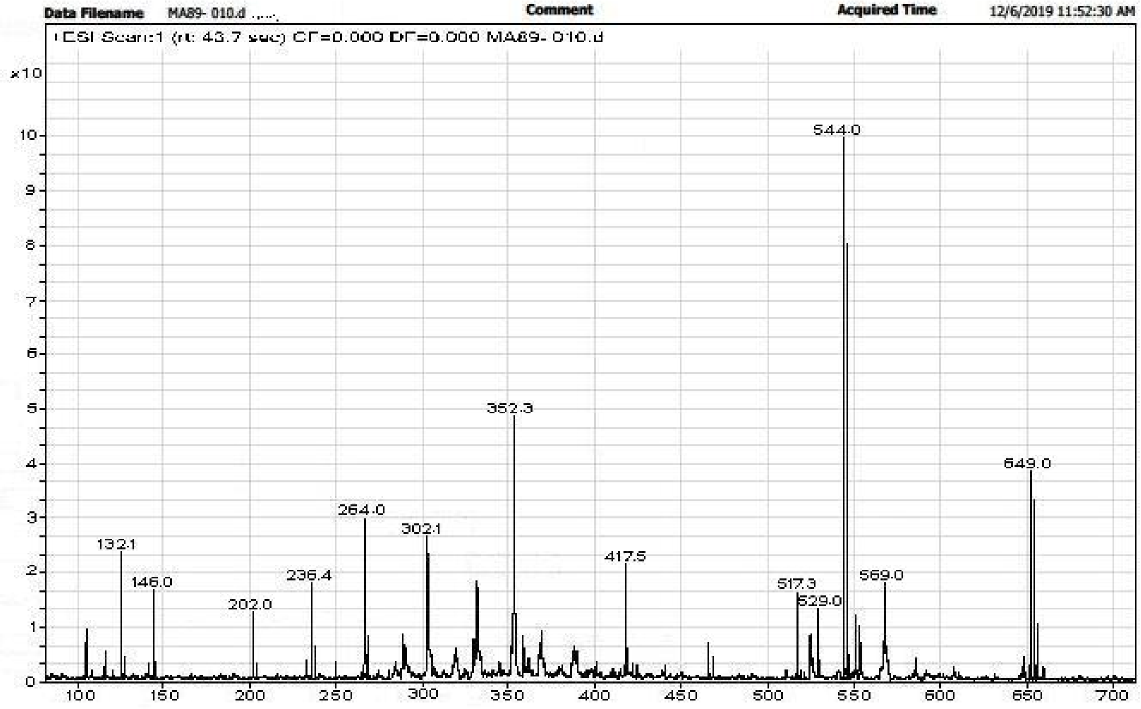

Figure S63: Mass Spectroscopy of Compound **10f**

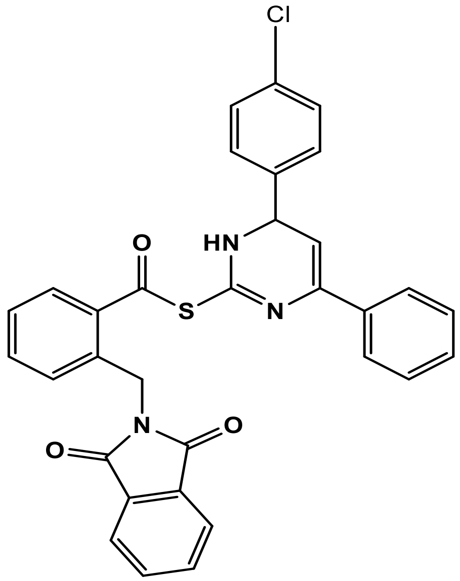

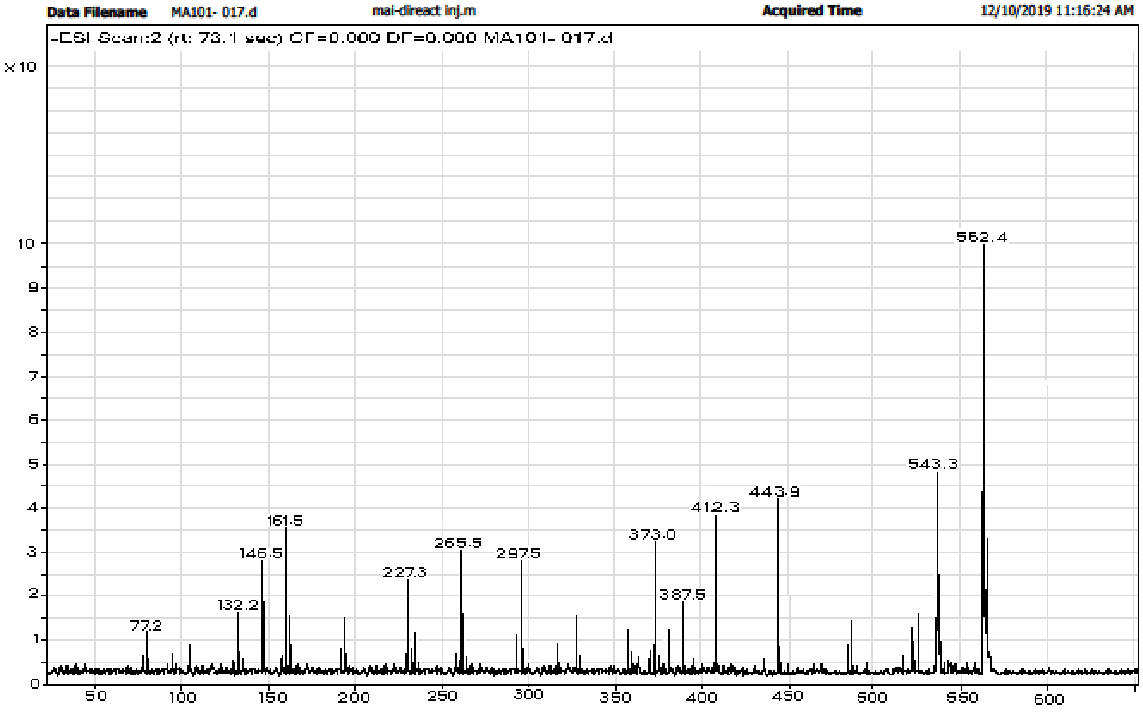

Figure S64: Mass Spectroscopy of Compound **10g**

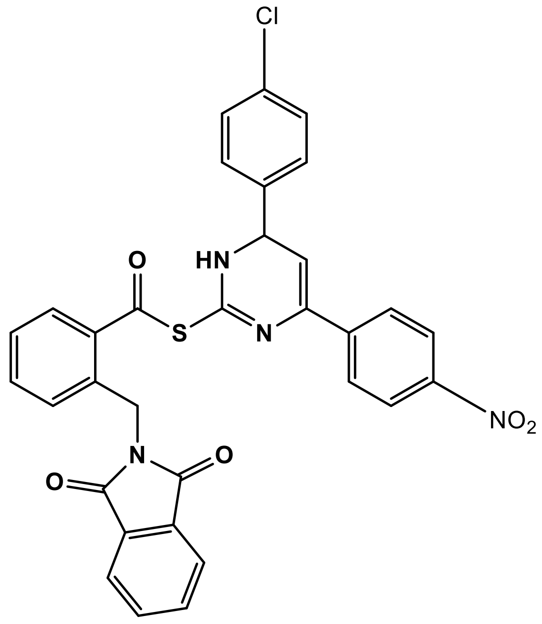

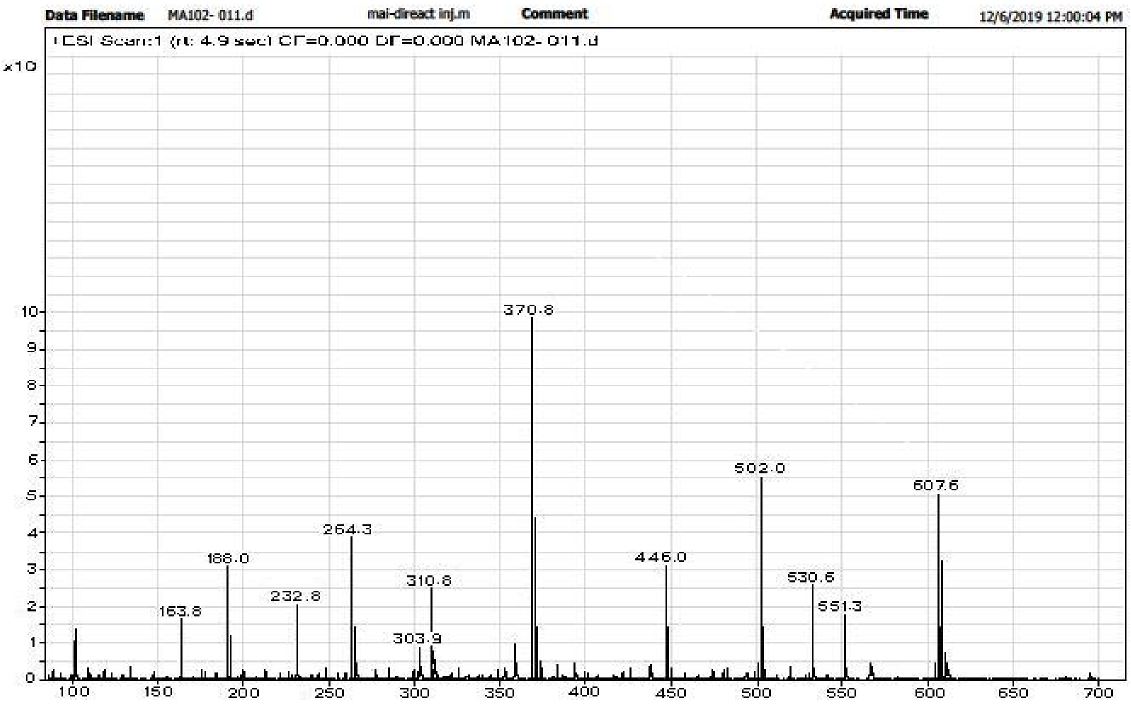

Figure S65: Mass Spectroscopy of Compound **10h**

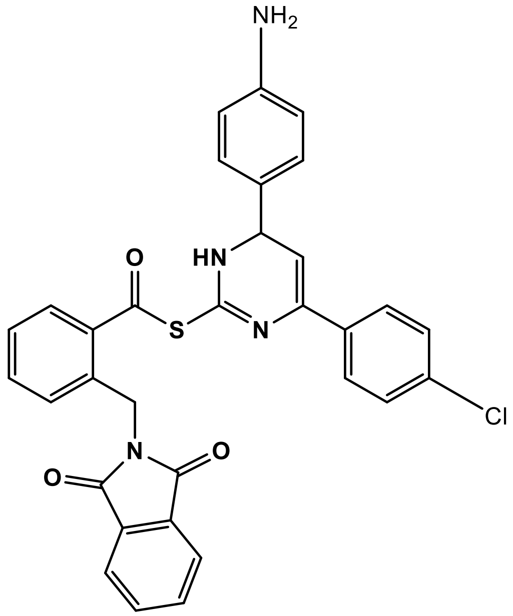

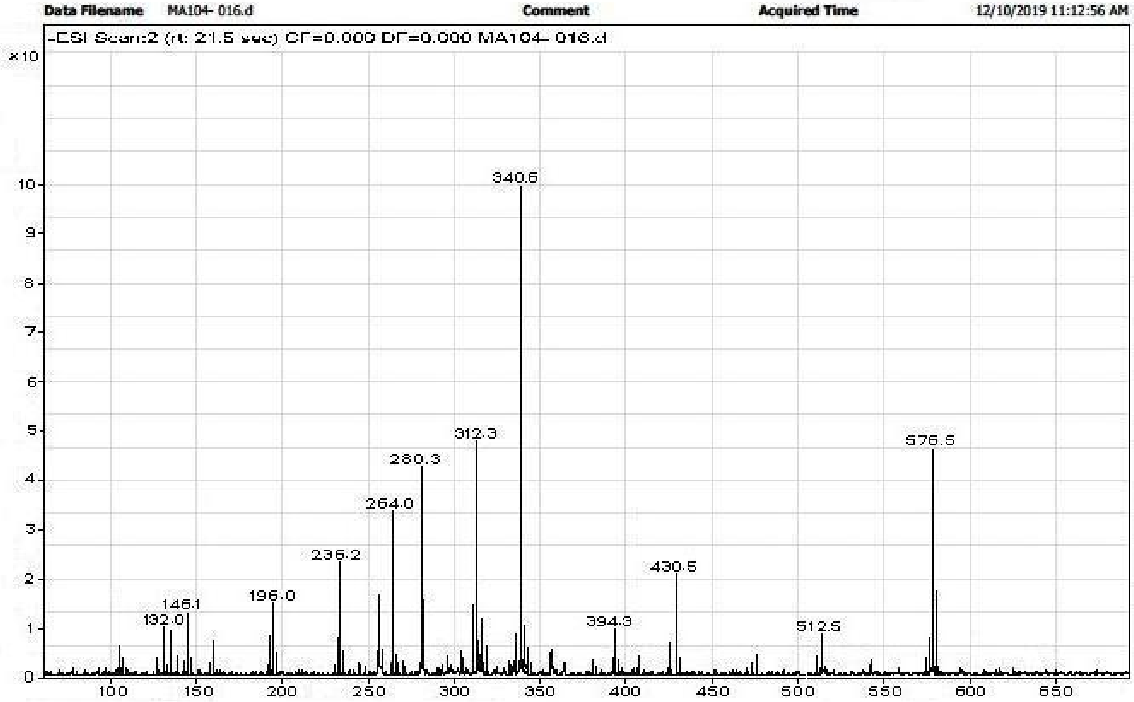

Figure S66: Mass Spectroscopy of Compound **10i**

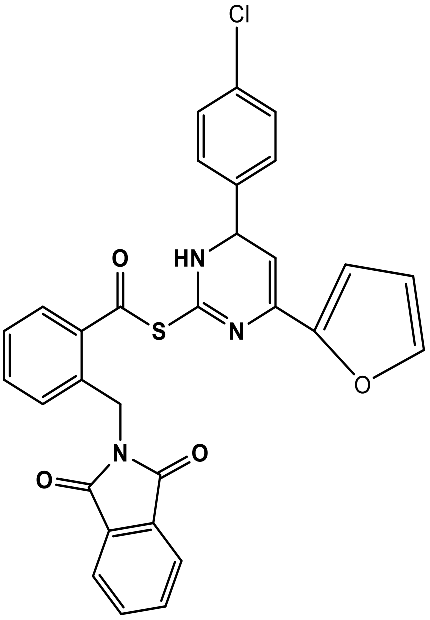

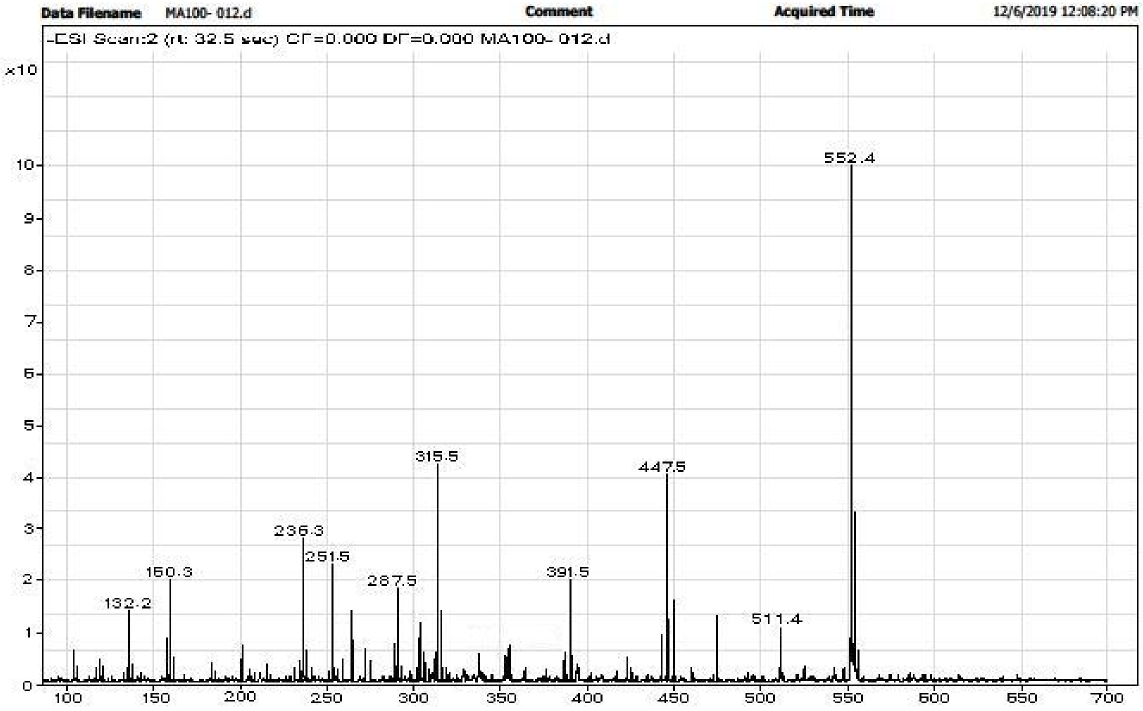

Figure S67: Mass Spectroscopy of Compound **10j**

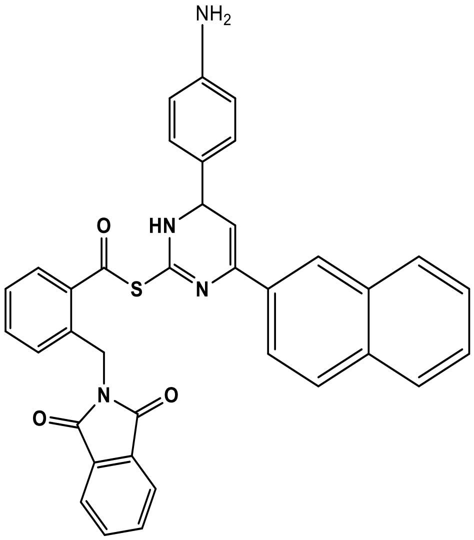

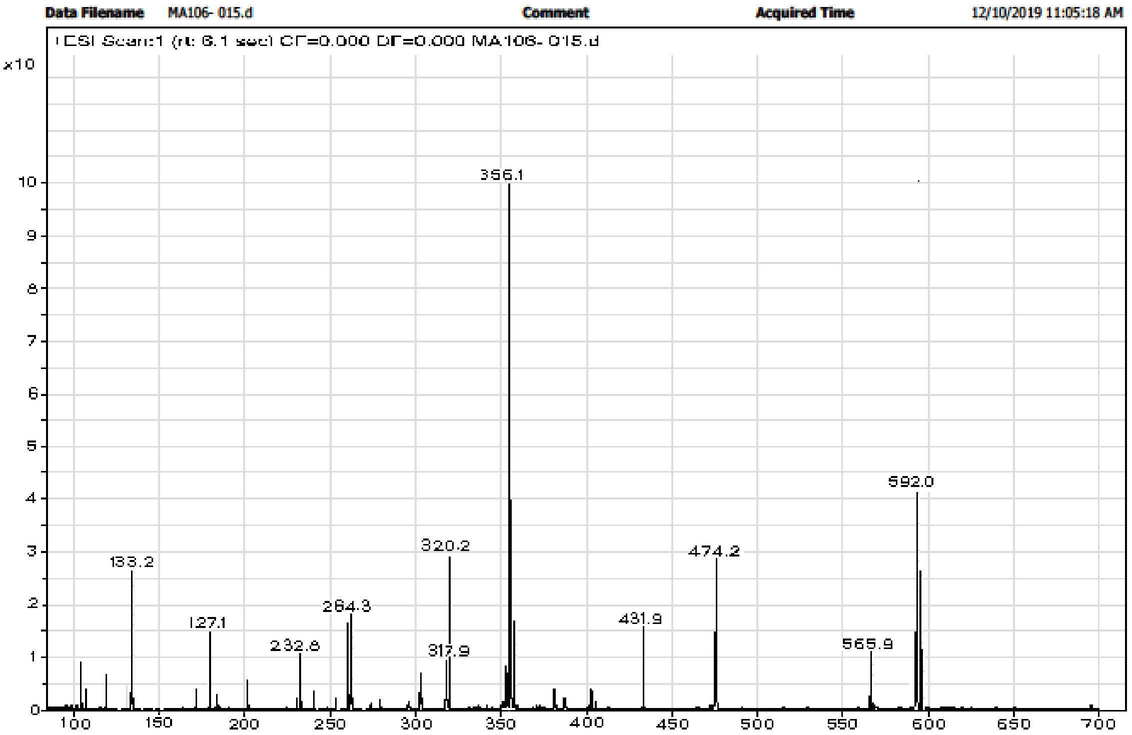

Supplement: Supplementary file 1 [file pharmaceuticals-14-00144-s001.pdf]
